# Age-dependent white matter microstructural disintegrity in autism spectrum disorder

**DOI:** 10.3389/fnins.2022.957018

**Published:** 2022-09-07

**Authors:** Clara F. Weber, Evelyn M. R. Lake, Stefan P. Haider, Ali Mozayan, Pratik Mukherjee, Dustin Scheinost, Nigel S. Bamford, Laura Ment, Todd Constable, Seyedmehdi Payabvash

**Affiliations:** ^1^Department of Radiology and Biomedical Imaging, Yale School of Medicine, New Haven, CT, United States; ^2^Social Neuroscience Lab, Department of Psychiatry and Psychotherapy, Lübeck University, Lübeck, Germany; ^3^Department of Otorhinolaryngology, University Hospital, Ludwig-Maximilians-Universität München, Munich, Germany; ^4^Department of Radiology and Biomedical Imaging, University of California, San Francisco, San Francisco, CA, United States; ^5^Departments of Pediatrics, Neurology, Cellular and Molecular Physiology, Yale University, New Haven, CT, United States

**Keywords:** autism, age, diffusion tensor imaging, connectome, white matter

## Abstract

There has been increasing evidence of White Matter (WM) microstructural disintegrity and connectome disruption in Autism Spectrum Disorder (ASD). We evaluated the effects of age on WM microstructure by examining Diffusion Tensor Imaging (DTI) metrics and connectome Edge Density (ED) in a large dataset of ASD and control patients from different age cohorts. *N* = 583 subjects from four studies from the National Database of Autism Research were included, representing four different age groups: (1) A Longitudinal MRI Study of Infants at Risk of Autism [infants, median age: 7 (interquartile range 1) months, *n* = 155], (2) Biomarkers of Autism at 12 months [toddlers, 32 (11)m, *n* = 102], (3) Multimodal Developmental Neurogenetics of Females with ASD [adolescents, 13.1 (5.3) years, *n* = 230], (4) Atypical Late Neurodevelopment in Autism [young adults, 19.1 (10.7)y, *n* = 96]. For each subject, we created Fractional Anisotropy (FA), Mean- (MD), Radial- (RD), and Axial Diffusivity (AD) maps as well as ED maps. We performed voxel-wise and tract-based analyses to assess the effects of age, ASD diagnosis and sex on DTI metrics and connectome ED. We also optimized, trained, tested, and validated different combinations of machine learning classifiers and dimensionality reduction algorithms for prediction of ASD diagnoses based on tract-based DTI and ED metrics. There is an age-dependent increase in FA and a decline in MD and RD across WM tracts in all four age cohorts, as well as an ED increase in toddlers and adolescents. After correction for age and sex, we found an ASD-related decrease in FA and ED only in adolescents and young adults, but not in infants or toddlers. While DTI abnormalities were mostly limited to the corpus callosum, connectomes showed a more widespread ASD-related decrease in ED. Finally, the best performing machine-leaning classification model achieved an area under the receiver operating curve of 0.70 in an independent validation cohort. Our results suggest that ASD-related WM microstructural disintegrity becomes evident in adolescents and young adults—but not in infants and toddlers. The ASD-related decrease in ED demonstrates a more widespread involvement of the connectome than DTI metrics, with the most striking differences being localized in the corpus callosum.

## Introduction

Autism Spectrum Disorder (ASD) is a neuropsychiatric condition characterized by impairments in communication and social interaction, repetitive behaviors and stereotypical interests (American Psychiatric Association [APA], 2013). ASD prevalence is estimated at 1 in 54 among 8-year-old children in the United States (Maenner et al., 2020). Difficulty of early diagnoses in children, and evidence of incurred benefit due to early and tailored treatment strategies highlight the need for improving diagnostic algorithms and treatment planning. Many etiological and pathophysiological theories of ASD involve genetic and environmental factors (Autism Genome Project Consortium et al., 2007; Chaste and Leboyer, 2012), as well as morphological correlates in the central nervous system. There has been increasing evidence of White Matter (WM) microstructural disintegrity in ASD (Alexander et al., 2007; Aoki et al., 2017; Payabvash et al., 2019b). Previous studies of WM microstructure in children with ASD vary in cohort size (*n* = 58—*n* = 213), mostly focus on specific age groups and are therefore limited in their ability to make assumptions of WM changes across the lifespan (Walsh et al., 2021).

In accordance with models emphasizing the abnormal interhemispheric interactions in ASD (Travers et al., 2012), many studies have identified the corpus callosum as the primary location for WM disintegrity in ASD. However, results diverge regarding the particular section within the corpus callosum (Alexander et al., 2007). Findings range from alterations in the whole corpus callosum (Shukla et al., 2010, 2011; Jou et al., 2011) to isolated changes in the splenium or anterior body (Brito et al., 2009; Kumar et al., 2010; Cheon et al., 2011). In addition, many studies have reported more pervasive WM microstructural disintegrity in the frontal and temporal lobes or dominant tracts (Barnea-Goraly et al., 2004; Ameis and Catani, 2015). These inconsistencies could be in part due to age range differences of participants across studies. Moreover, previous evidence is limited as only few studies investigate Diffusion Tensor Imaging (DTI)—the majority of neuroimaging studies have focused on functional and structural MRI and show notable variations in cohort composition, which could mask sex-, age- and ASD-related effects (Walsh et al., 2021).

A more detailed knowledge of age-adjusted microstructural correlates of ASD may improve diagnostic algorithms, facilitate early therapeutic intervention, and provide potential objective biomarkers to monitor treatment response. To draw more robust and meaningful conclusions about age- and ASD-related alterations of WM microstructure, we analyzed diffusivity and tractography among subjects from four different age-group study cohorts in the National Database of Autism Research (NDAR). DTI and T_1_-weighted images were used to assess voxel-wise and tract-based differences between ASD patients and typically developing controls (TDC). In addition to conventional DTI-driven measurements, we analyzed edge density (ED) (Owen et al., 2015) as a representation of white matter connectivity. Tract-based metrics were analyzed using both conventional statistical methods as well as combinations of several feature selection algorithms and machine learning classifiers in a multimodal approach.

## Materials and methods

### Study cohorts

**FIGURE 1:**
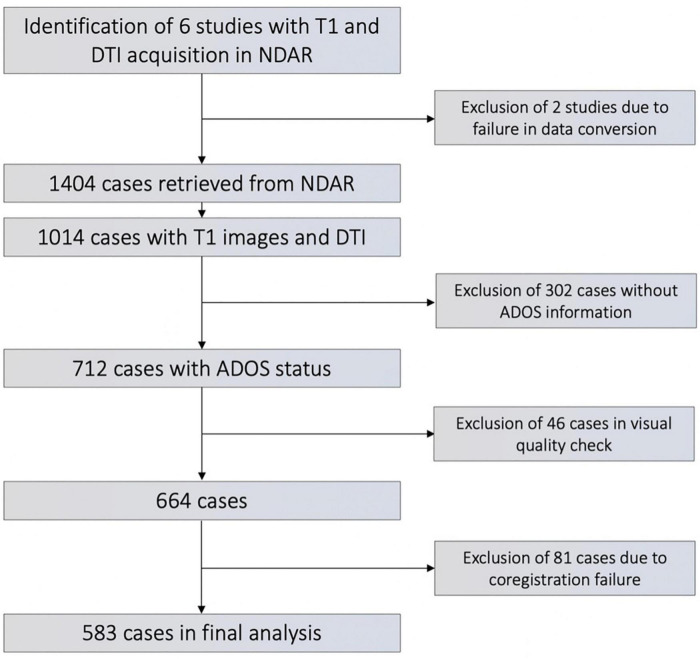
Flowchart of subject’s inclusion/exclusion.

We retrieved all datasets from the National Database of Autism Research (NDAR) that had DTI- and T_1_-weighted imaging data available. Subjects from four study cohorts were included, each representing a different age group: (1) A Longitudinal MRI Study of Infants at Risk for Autism (infants) (Piven, 2017); (2) Biomarkers of Autism at 12 months (toddlers) (Courchesne, 2012); (3) Multimodal Developmental Neurogenetics of Females with ASD (adolescents) (Pelphrey, 2017); and (4) Atypical Late Neurodevelopment in Autism: A Longitudinal MRI and DTI Study (young adults) (Lainhart, 2012). We excluded subjects with genetic comorbidities such as fragile X syndrome, insufficient clinical information, evident artefacts on brain scans, and those with failures in image processing, such as coregistration failure. Figure 1 depicts all inclusion and exclusion criteria in a flowchart. Subjects were allocated to ASD versus typically developing controls (TDC) groups based on the Autism diagnostic schedule (ADOS) diagnosis, which was assessed by age- and development-adjusted algorithms in the original studies. Detailed information about the age and sex composition of each study cohort is listed in Table 1.

**TABLE 1:** Study cohort demographics.

Study	Median age (IQR)	ASD/TDC	Male (%)
Longitudinal MRI study of infants at risk of autism (*n* = 155)	7 (6–7) months	34/121	65.8%
Biomarkers of autism at 12 months (*n* = 102)	32 (25–36) months	57/45	73.5%
Multimodal developmental neurogenetics of females with autism (*n* = 230)	13.1 (5.3) years	106/124	50.9%
Atypical late neurodevelopment in autism (*n* = 96)	19.1 (10.7) years	67/29	99.0%

ASD, autism spectrum disorder; TDC, typically developing controls. Age is represented as median (interquartile range).

### Image acquisition protocols and preprocessing

The acquisition protocols differed across studies. In the infant cohort, T_1_-weighted imaging was conducted with a repetition time (TR) of 2400 ms, time to echo (TE) of 3.16 ms, field of view (FOV) of 256, matrix size 224 × 256, and slice thickness 1 mm, diffusion weighted images were acquired in 26 variable *b*-values between 50 and 1000 s/mm^2^ increasing by 200 s/mm^2^ at each scan (25 gradient directions and one non-weighted image with *b* = 0 s/mm^2^) image on 3T Siemens Tim Trio, with TR = 12,800–13,300 ms, TE = 102 ms, FOV 190, matrix size 190 × 190, and slice thickness of 2 mm. Toddlers’ T_1_-weighted imaging was acquired with TR = 6500 ms, TE = 2.8 ms, FOV = 240, matrix size 96 × 96, slice thickness 1.2 mm, DTI included 51 images with *b* = 1000 s/mm^2^ and one non-weighted *b* = 0 s/mm^2^ image acquired on 1.5 T GE Signa HDxt, TR = 13200 ms, TE = 80.6 ms, FOV 240, matrix size 96 × 96, and slice thickness 2.5 mm. Adolescents’ T_1_-weighted imaging was acquired with TR = 5300 ms, TE = 3.3 ms, FOV 350, matrix size 192 × 192, slice thickness = 1 mm, DTI included 46 images with *b* = 1000 s/mm^2^ and one non-weighted *b* = 0 s/mm^2^ image acquired on 3T Siemens Magnetom TrioTim, TR = 13,000 ms, TE = 93 ms, FOV 250, matrix size 192 × 192, and slice thickness 2.5 mm. Adults’ T_1_-weighted imaging was acquired with TR = 1800, TE = 1.93, FOV 256, matrix size 256 × 240, slice thickness 1 mm, DTI included 4 repetitions of 12 images with *b* = 1000 s/mm^2^ and followed by an image with *b* = 0 s/mm^2^ acquired on 3T Siemens Magnetom TrioTim, with TR = 7000 ms, TE = 91 ms, FOV = 256, matrix size 128 × 128, and slice thickness 2.5 mm.

All images in DICOM (Digital Imaging and Communication in Medicine) format were converted to Nifti format using *dcm2nii* (Maenner et al., 2020) tool, with extraction of diffusion gradient directions. Images in Medical Imaging NetCDF (MINC) format were converted using *mnc2nii* tool in the FreeSurfer software package (Fischl, 2012), and diffusion gradient direction was extracted from header information.

### Diffusion tensor imaging processing pipeline

We preprocessed all DTI images using FSL eddy current correction and brain extraction tool (Smith, 2002; Smith et al., 2004), and subsequently generated fractional anisotropy (FA), mean- (MD), and axial Diffusivity (AD) maps using FSL’s diffusion tensor fitting program (DTIFIT). FSLmaths was used to derive radial diffusivity (RD) as the average of the second and third eigenvalues. In order to obtain ED maps, we first applied FSL Bayesian estimation of diffusion parameters obtained using sampling techniques (BEDPOSTX) on FA maps (Behrens et al., 2007), which can overcome limitations of tensor-based representations of diffusivity by identifying crossing fibers. BEDPOSTX results were then fed into probabilistic tractography using FSL PROBTRACKX (Behrens et al., 2003), which was then used for generation of ED maps. We specified seed and waypoint masks as 48 cortical and 7 subcortical nodes per hemisphere as defined in the Harvard-Oxford cortical and subcortical atlas (Frazier et al., 2005; Desikan et al., 2006; Makris et al., 2006; Goldstein et al., 2007) (list provided in Supplementary material; 2.2 Harvard-Oxford Cortical Atlas as well as 2.3 Harvard-Oxford subcortical atlas). These seed and waypoint masks were registered to each individual’s native FA space using FSL’s linear coregistration tool FLIRT (Smith et al., 2004). The probabilistic tractography-derived ED maps reflect the density of connectome edges (links) between nodes representing the landmark anatomical structures of cerebral gray matter (Owen et al., 2015).

### Voxel-wise analysis using tract-based spatial statistics

Voxel-wise analysis of diffusivity metrics was carried out using FSL tract-based spatial statistics (TBSS) (Smith et al., 2006). As described previously (Payabvash et al., 2019b), we coregistered all FA maps to a common space by the standard FSL TBSS pipeline, where all images were non-linearly coregistered to a standard template, in this case the most typical subject in each cohort (-n option). Then, we created a mean skeleton of the highest FAs that represents the center of WM tracts. All FA maps, as well as other diffusivity metrics in respective analyses, were then non-linearly coregistered onto the mean FA skeleton using the FSL non-linear registration tool before performing cross-subject statistics. General linear models (GLM) were used to assess the influence of age, sex and ASD diagnosis. To minimize the effects of data heterogeneity, we conducted analyses for each site separately as acquisition parameters differed between study cohorts. For non-parametric voxel-wise statistics, we applied FSL “randomize” (Winkler et al., 2014) with 5000 permutations and family wise error (FEW) correction of *p*-values followed by threshold-free cluster enhancement (TFCE) (Smith and Nichols, 2009).

### Tract-based analysis

To confirm the results of voxel-wise analysis, we also evaluated the relationship of the averaged diffusion metrics and ED in WM tracts with the age, ASD diagnosis and sex in different study cohorts. We extracted FA, MD, RD, and AD metrics of each of the 48 white matter tracts specified in the John Hopkins University (JHU) white matter tracts labels atlas (Wakana et al., 2007; O’Donnell et al., 2009) by determining the non-zero mean of each individual’s image within the respective tract. In order to provide similar analysis to voxel-wise method, for tract-based analysis, measurements were performed in a standard space of MNI-152. A list of all tracts considered is given in Supplementary material (2.1 John Hopkins University White Matter Label Atlas). Given that the brainstem was set as termination mask in fiber tracking for generation of ED maps (Owen et al., 2015), the averaged ED of corticospinal tracts, medial lemnisci and pontine crossing fibers were excluded from tract-based analysis. Tract-based metrics were evaluated for the influence of ASD diagnosis, age, and sex using multiple regression analyses followed by *p*-value correction using false discovery rate (FDR) in R software (version 4.0.2) (R Core Team, 2020). We conducted analyses for behavioral measures for a subset of adolescents in which ADOS (Lord et al., 2000) scores (*n* = 86) were available, as well as in a subset of the adult cohort in which Wechsler Intelligence Quotient (IQ) (Saklofske and Schoenberg, 2011) for 71 individuals (50 ASD, 21 TDC) as well as Social Responsiveness Scale (SRS) (Constantino, 2013) scores for 50 individuals (33 ASD, 17 TDC) were measures. Utilizing a voxel-wise GLM, the influence of aforementioned scores on diffusivity metrics were tested after adjustment for age.

### Machine learning

To evaluate the feasibility of machine learning algorithms for the prediction of an ASD diagnosis based on diffusion and connectome-based metrics. Given the results of voxel-wise and tract-based analysis, we included data from the adolescent (*n* = 176) and adult (*n* = 74) cohorts (Table 2). We applied combinations of six different classifiers and five feature selection algorithms using FA, MD, RD, AD, and ED of white matter tracts separately and combined as input. The diffusion metrics from all WM tracts were included in corresponding analysis pipeline—e.g., the averaged FA from 48 WM tracts were included as input for FA based analysis, and all diffusion metrics were included in combination analyses. The respective feature selection algorithms and classifiers are further detailed in Supplementary material(3. Machine Learning). Machine learning analysis was based on a framework previously described by Haider et al. (2020). Subjects were randomly split into a training/cross-validation set (*n* = 250) and an independent test set (*n* = 76) which was completely isolated from training process, with similar ASD-to-TDC-ratio as study cohort distribution. For each combination of classifier and feature selection algorithm, we created a framework of 20 repeats of five-fold cross validation, stratified based on ASD diagnosis, to perform hyperparameter optimization and identify the best performing models. Using Bayesian optimization, the hyperparameters of each machine learning model as well as the number of features included in the model were fine tuned. Upper and lower bounds of each hyperparameter (which was optimized), and the number of tuning repetitions are included in Supplementary Table 2. Subsequently, each model’s cross validation framework was applied with tuned hyperparameters to evaluate each model’s performance based on the mean area under the curve (AUC) of receiver operating characteristics (ROC) across validation folds, which reflects on both the true-positive and false-positive rate and therefore eliminates biases by original case-control distribution. Finally, we trained the optimal model on the whole training/cross-validation cohort (*n* = 250) with optimized hyperparameters and evaluated the performance in the independent test set (*n* = 76). We also determined sensitivity and specificity at balanced prediction probability cutoff, using a confusion matrix. All analyses were performed using R (version 4.0.2) (R Core Team, 2020).

**TABLE 2:** Distribution of subjects from each cohort among the training/cross-validation versus independent test set, using only data from studies where we found significant ASD-related alterations.

Study	Training			Independent validation		
	No	Age	ASD/TDC	No	Age	ASD/TDC
Multimodal developmental neurogenetics	176	155 (124–182)	82/94	54	162 (119–194)	24/30
Atypical late neurodevelopment in autism	74	242 (194–320)	52/22	22	211 (182–256)	15/7

ASD, autism spectrum disorder; TDC, typically developing children. Age is represented as median (interquartile range) in months.

## Results

### Age-dependent alterations of white matter microstructure and connectome edge density imaging

**FIGURE 2:**
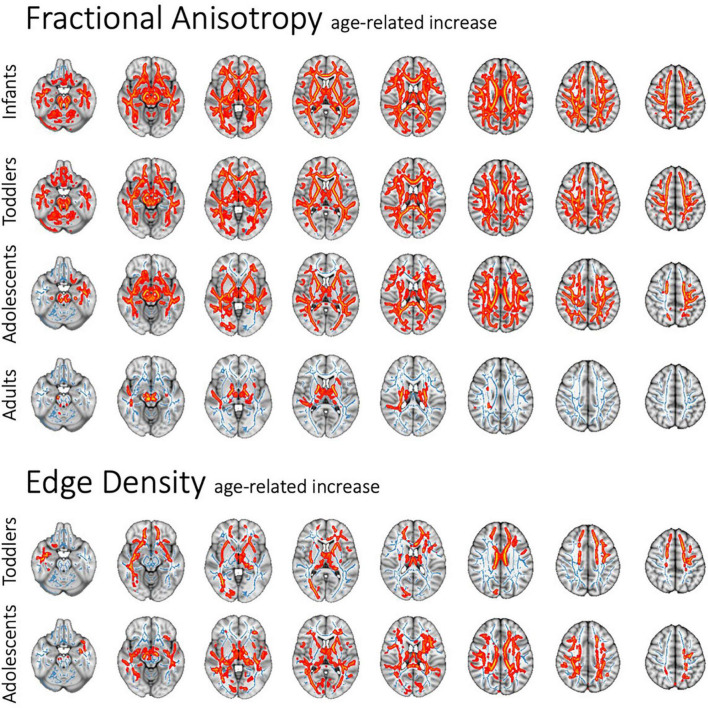
Age-related pervasive decrease in FA and (to lesser degree) ED throughout WM tracts in voxel-wise analysis, independent of ASD diagnosis or sex. Figure shows the standard MNI152 brain template overlaid with the mean FA skeleton resulting from TBSS (blue), as well as all significant changes (*p* < 0.05) (red).

In voxel-wise TBSS analysis, we examined the influence of age on DTI metrics and connectome ED within each study cohort, while correcting for ASD diagnosis as a covariate. There was a pervasive age-related FA increase in infants, toddlers and adolescents (Figure 2), as well as a corresponding decline in MD, RD, and AD independent of ASD diagnosis status. In adults, the age-related FA increase was predominantly along the corticospinal tract (Figure 2). Supplementary Figures 3–5 demonstrate the age-dependent changes in DTI metrics among different study cohorts. ED assessment of the brain connectome showed an age-related increase in ED among toddlers and adolescents (Figure 2). In toddlers, increasing age was associated with higher ED in commissural tracts as well as frontal, occipital and temporal association tracts. In adolescents, an age-related increase in connectome ED was mainly localized to the posterior corpus callosum. We confirmed findings of voxel-wise analyses in multiple regression analyses of tract-based metrics (Supplementary Table: 4 Tract-Based Multiple Regression).

### Autism spectrum disorder-related alterations of white matter microstructure and connectome edge density imaging

**FIGURE 3:**
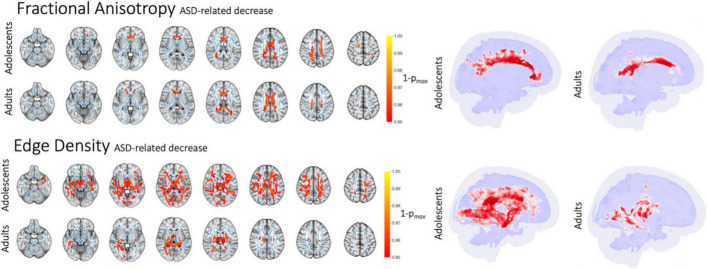
ASD-related decrease in FA and ED in voxel-wise analysis after correction for age. Figure shows white matter tracts with significant difference in red (*p* < 0.05) overlaid on the mean FA skeleton resulting from TBSS (blue).

In voxel-wise analyses, after correcting for age, ASD diagnosis was associated with lower FA in commissural tracts within the corpus callosum among adolescents and adults (Figure 3), but not in infants or toddlers. Corresponding increases in MD and RD were found in adults, and a slight decrease of AD was present in adolescents (Supplementary Figure 6). Compared to DTI diffusion metrics, ED revealed a more widespread reduction in connectome edges among subjects with ASD in the adolescent and adult cohorts. Among adolescents, ASD was associated with an extensive decrease in the ED of WM tracts—except for the internal capsule—after correction for age and sex as covariates. In the adult cohort, ASD was associated with lower ED within the posterior commissural and paraventricular WM tracts. Infants showed an isolated decrease in ED in the left sagittal stratum which was related to ASD. There were no significant changes in the toddler cohort as assessed in a voxel-wise GLM correcting for age and sex. Tract-based multiple regression analyses confirmed voxel-wise findings with significant ASD-related alterations of white matter diffusion metrics and connectome edges only found among adolescents and adults adjusting for age and sex.

### Sex-related changes in white matter microstructure

**FIGURE 4:**
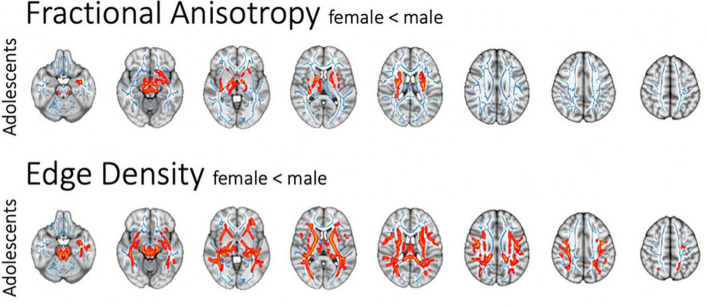
Sex-related changes in FA and ED as assessed in voxel-wise analysis after correction for age and ASD diagnosis. The white matter tracts with significant difference are colored red (*p* < 0.05) and overlaid on the mean FA skeleton (blue).

In the adolescent cohort, there was lower FA along corticospinal tracts in females as compared to males when correcting for ASD diagnosis and age (Figure 4). We could not detect corresponding sex-related differences in the MD, RD, and AD of white matter tracts. In the adolescent cohort, ED revealed a more pervasive sex-related reduction in connectome ED among females compared to males. Tract-based multiple regression confirmed the results of voxel-wise analyses. This effect could not be found in younger cohorts after correcting for the covariate of age; the adult cohort only included one female subject and could therefore not be used to assess and make assumptions about sex-specific alterations. Of note, there was no significant difference between males and females regarding ADOS-scores in the adolescent cohort in a two-sided *t*-test (t-statistic 1.46, *p* = 0.148).

### Machine learning classifiers predicting autism spectrum disorder diagnosis from tract-based diffusion tensor imaging and connectomics

**FIGURE 5:**
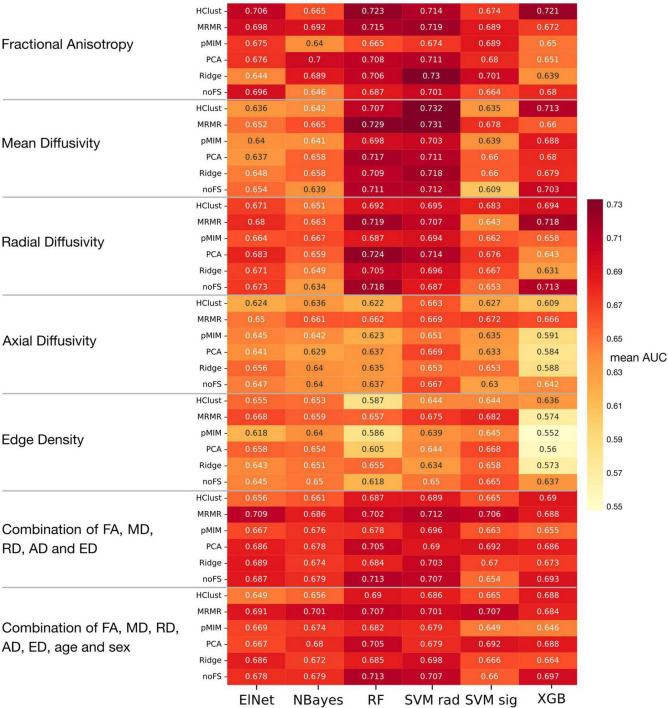
Heatmap of mean AUC across 100 validation folds in machine learning analysis. Abbreviations see Table 2.

Figure 5 displays a heatmap demonstrating the mean averaged AUC across validation folds from twenty repeats of five-fold cross validation, considering data from the adolescent and adult cohort (as mentioned above). The average AUC values range from 0.55 to 0.73, with the highest performance achieved using a support vector machine with radial kernel in combination with hierarchical clustering as feature selection applied to MD metrics. This combination model achieved 0.696 AUC (95% Delong CI 0.578–0.800), 63.2% accuracy, 56.1% sensitivity, and 71.4% specificity in the independent validation cohort.

### Psychological/cognitive performance and brain microstructure

We found no significant influences of ADOS (Lord et al., 2000) scores on diffusivity metrics. In the adult cohort, there was no significant influence of IQ and SRS scores on diffusivity metrics after correction for age.

## Discussion

Using, multicentric data from different age groups, we found an age-related increase in FA and ED, and decrease in MD and RD throughout most WM tracts. After correction for age and sex, ASD diagnosis was associated with WM microstructure disintegrity in adolescent and adult age groups primarily in the corpus callosum. However, ED revealed a more pervasive involvement of the brain’s connectome in adolescents and adults with an ASD diagnosis. We also suggest a potential role for applying machine learning classifiers to assist with ASD diagnosis based on tract-based inputs from DTI scans. Limited by the small numbers of female subjects in our study cohorts, we found reduced FA in corticospinal tracts of female adolescents compared with males after correcting for ASD diagnosis and age.

### Age-related alterations in diffusivity and anisotropy metrics

We found a pervasive rise in FA with increasing age throughout WM tracts in all study cohorts. In infants and toddlers, these changes are likely related to increased myelination of axon fibers and the microstructural maturation of WM (Yu et al., 2020). Research about developmental gradients in functional connectivity and WM microstructure suggests that even though most of WM maturation is completed during toddlerhood, further waves of myelination between association cortices with corresponding DTI changes occur in adolescence and adulthood (Sydnor et al., 2021), which may explain our findings among adolescents and older adults. ED can provide further insight into the brain connectome by examining potential fiber tracts between cortical nodes instead of mere assessment of directional water movement by conventional DTI metrics. We found a significant ED increase with age in toddlerhood and adolescence, which aligns with brain maturation in these age groups. Our findings highlight the necessity of adjusting the analysis of ASD-related microstructural changes for a respective subjects’ age.

### Autism spectrum disorder-related white mater microstructural alterations center in the corpus callosum

Our results suggest that microstructural disintegrity related to ASD is likely more evident in adolescents and young adults, but hardly detectable in younger age groups. These ASD-related changes in WM microstructure are primarily in the anterior and mid corpus callosum (Alexander et al., 2007; Payabvash et al., 2019b). While existing literature presents inconsistent results regarding WM alterations in the particular location within the corpus callosum, our data suggests that DTI abnormalities in the anterior corpus callosum are present in adolescents, become more pronounced and extend to central and posterior commissural tracts with increasing age. Notably, the infants and toddlers age groups included in our analyses are younger than most previous studies (Li et al., 2017). We also found significant ED reductions in central WM tracts related to ASD diagnosis, which were present across most WM tracts in adolescents, but more concentrated to posterior tracts in adults. These findings suggest a more pervasive involvement of the brain connectome compared to what is captured by conventional DTI metrics. Previous studies also show ED alterations in periventricular WM associated with ASD and other neurodevelopmental disorders (Payabvash et al., 2019a,b). In addition, significant changes could be found in a small area within the internal capsule of infants, but not toddlers.

### Sex-specific effects

We found significantly lower FA in association and commissural tracts of female adolescents compared to males after correcting for age and ASD diagnosis (Figure 4). Previous studies suggested a correlation between sex and diffusivity metrics, as well as sex-related differences in correlation between DTI and behavioral measurements (Waller et al., 2017). A recent review of MRI studies evaluating sex-related effects in ASD also found that DTI metrics differ significantly between male and female subjects (Walsh et al., 2021). The female protective effect and extreme male brain hypothesis suggest that sex-related characteristics of brain organization contribute to ASD symptom severity (Werling and Geschwind, 2015).

There have been extensive discussions about sex-related effects in ASD, as the influence of data assessment bias on hypotheses of etiological aspects is hard to quantify. Bias persists due to later diagnosis of females as stereotypical female behavior aligns better with ASD symptoms and currently used diagnostic algorithms might camouflage ASD diagnosis in females (Ratto et al., 2018). The previously approximated male-to-female ratio of 4:1 has been found to vary between 3:1 and 8:1 depending on quality of ASD assessment, and is nowadays estimated to trend toward a more equal sex distribution (Loomes et al., 2017). We could not find a significant interaction between sex and ASD diagnosis; however, our results underline the importance of considering sex in the investigation of ASD-related alterations.

### Machine learning analysis

Machine learning algorithms provide a statistically suitable platform for the analysis of high-dimensional multimodal neuroimaging data, which may account for the complexity of underlying neurobiological changes better than traditional statistical methods. In this study, we showed the feasibility of machine learning algorithms in assisting with the diagnosis of ASD based on DTI metrics of WM tracts. Using a rigorous cross-validation scheme, we optimized, trained, tested and validated an optimal combination of feature selection model and machine learning classifier to predict ASD diagnosis based on average DTI- and connectome derived metrics of WM tracts among adolescents and adults. Machine learning algorithms can potentially evaluate large amounts of imaging data and give detailed information about the individual’s structural connectome. Therefore, algorithms could become a useful tool to consolidate diagnostic algorithms, subsequently improving ASD diagnosis and further differentiate ASD from other neurodevelopmental abnormalities.

## Limitations

While analyses of large, multicentric data is a strength of our study, it also poses a limitation due to the heterogeneity of the data and difference in image acquisition techniques. However, given that voxel-wise and tract-based analyses were conducted separately for each site, we minimized the effects of site-specific differences. Of note, harmonization of diffusion MRI has been proposed to mitigate the issue of data acquisition from different scanners and centers (Pinto et al., 2020); but, given that the four cohorts in our study differ in terms of scanner, acquisition method, age of children, female-to-male ratio, and the rate of ASD among subjects, an optimal and fair harmonization technique might not be achieved. Moreover, although we could include 197 female subjects in our analyses (see Table 1), the majority of included individuals across different cohorts were male (51–99%), which impairs the generalizability of these results to female patients. We recognize that existing biases in ASD diagnoses extends from original assessments to our analyses. Some inaccuracies also come along with coregistration in TBSS and VBM, especially for pediatric cohorts with high variability in anatomy, motion, and head position. To mitigate these limitations, we excluded all cases with motion artefacts in a visual quality control. Finally, for our subjects, common coregistration templates such as MNI152 are limited in their accuracy, which is why we avoided using generic templates and chose study-specific ones wherever possible. We accounted for possible inaccuracies in voxel-wise analyses by confirming findings in tract-based statistics.

## Conclusion

Overall, our results portray a comprehensive assessment of WM microstructure and connectome ED across a wide age range. Based on a large multicentric dataset, we showed age-specific microstructural and connectome abnormalities of WM tracts in ASD. We found a ubiquitous age-related FA increase and diffusivity decrease across different age groups, which highlights the need for age adjusted assessments of ASD-related microstructural alterations. We found an increasing discrepancy between ASD and typically developing individuals with regards to microstructural integrity of anterior commissural tracts starting in adolescents and becoming more pronounced in young adults. ED showed even more extensive involvement of brain connectome in adolescents and young adults with ASD. Additionally, female individuals presented with lower FA values and higher diffusivity in central cerebral white matter after adjustment for age and ASD diagnosis. These findings support previous literature about sex-related effects in DTI metrics and add to sex-related hypotheses in ASD neurobiology. Finally, we showed the feasibility of machine learning classifiers in prediction of ASD diagnosis based on tract-based diffusion and connectome metrics. This creates a foundation for the future application of machine learning in DTI analyses, where such models can integrate multi-modal data to build more robust and generalizable statistical frameworks. Further research is needed to adequately assess the interaction between WM microstructural alterations and symptom severity in ASD.

## Data availability statement

Publicly available datasets were analyzed in this study. This data can be found here: https://nda.nih.gov.

## Author contributions

CW carried out image processing, analyses, interpreted the findings, wrote, and revised the manuscript. SH assisted in data analysis and in interpretation of findings and revised the manuscript. PM, EL, DS, NB, LM, and TC assisted in interpretation of findings and revised the manuscript. SP designed the study, interpreted findings, and contributed to writing and revision of the manuscript. All authors contributed to the article and approved the submitted version.
