# Supplementary material for: Age-dependent white matter microstructural disintegrity in autism spectrum disorder
Source: Front Neurosci. 2022 Sep 7;16:957018. doi: 10.3389/fnins.2022.957018 (PMC9490315; doi:10.3389/fnins.2022.957018)
Supplement: Supplementary file 1 [file Data_Sheet_1.pdf]

Supplemental Material

1. Supplementary Figures ... 2
2. List of Atlas Regions ... 8
   2.1 John Hopkins University White Matter Label Atlas ... 8
   2.2 Harvard-Oxford Cortical Atlas ... 8
   2.3 Harvard-Oxford Subcortical Atlas ... 9
3. Machine Learning ... 10
   3.1 Classifiers ... 10
   3.1.1 Elastic Net (ElNet) ... 10
   3.1.2 Naive Bayes (NBayes) ... 10
   3.1.3 Random Forest (RF) ... 11
   3.1.4 Support Vector Machine (SVM) with sigmoid (SVM sig) and radial (SVM rad) kernel 11
   3.1.5 Extreme Gradient Boosting (XGB) ... 11
   3.2 Feature Selection Algorithms ... 12
   3.2.1 Hierarchical Clustering (HClust) ... 12
   3.2.2 Minimal Redundancy Maximal Relevance (MRMR) ... 12
   3.2.3 Pearson correlation-based redundancy reduction with Mutual Information Maximization filter (pMIM) ... 13
   3.2.4 Principal Component Analysis (PCA)-based feature selection ... 13
   3.2.5 Logistical Regression using Ridge regularization (RIDGE) ... 13
4 Tract-Based Multiple Regression ... 17
   4.1 Infant Cohort ... 17
   4.2 Toddler Cohort ... 20
   4.3 Adolescent Cohort ... 23
References ... 29

1. Supplementary Figures

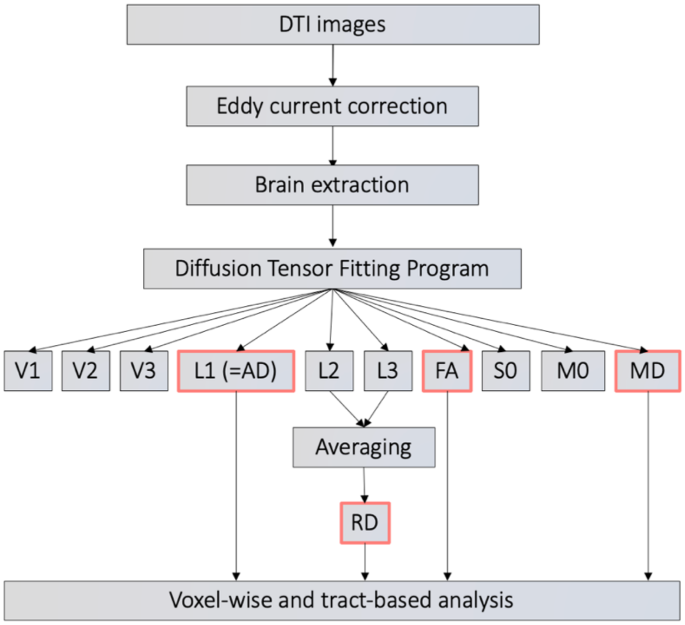

Suppl. Figure 1: Flowchart of DTI Preprocessing pipeline using FSL Diffusion Toolbox (Eddy Current Correction, Brain Extraction Tool, Diffusion Tensor Fitting Program). DTI metrics that were used in subsequent analyses are outlined in red.

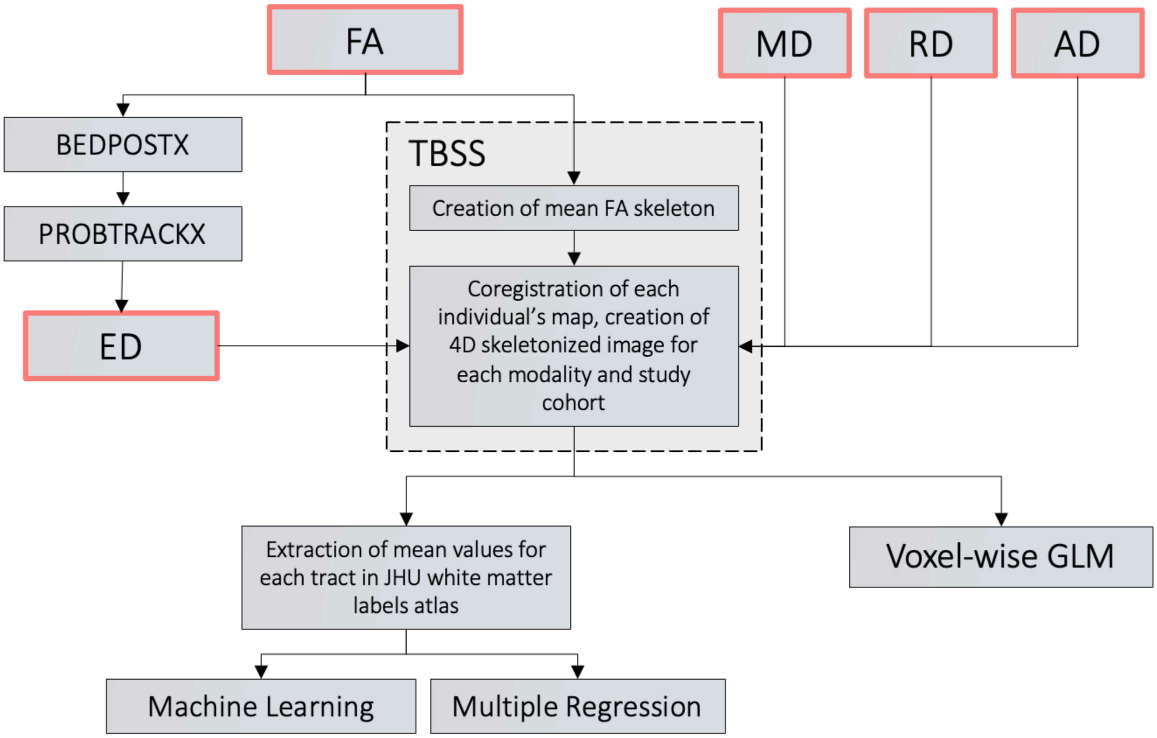

Suppl. Figure 2: Flowchart of further analysis steps, using FSL Tract-Based Statistics (TBSS), Voxel-Based Morphometry (VBM), and probabilistic tractography.

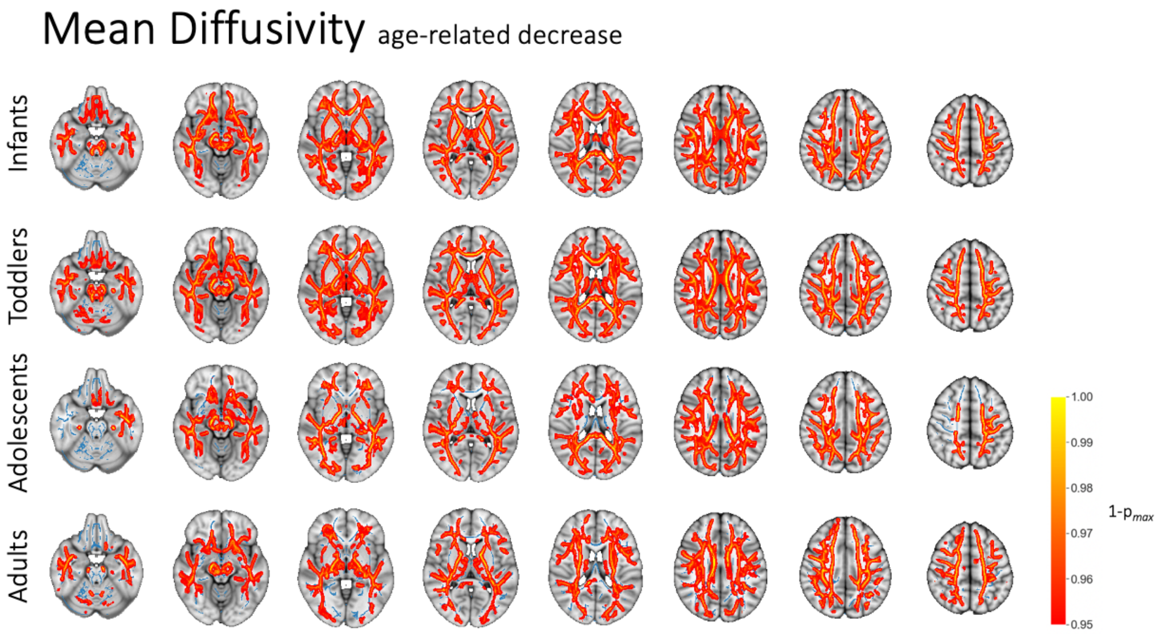

Suppl. Figure 3: Age-related changes in Mean Diffusivity in all four study cohorts. Figures show the MNI152 standard brain template with mean FA skeleton and significant changes ($p<0.05$) as assessed in voxel-wise GLM (using FSL TBSS) in red.

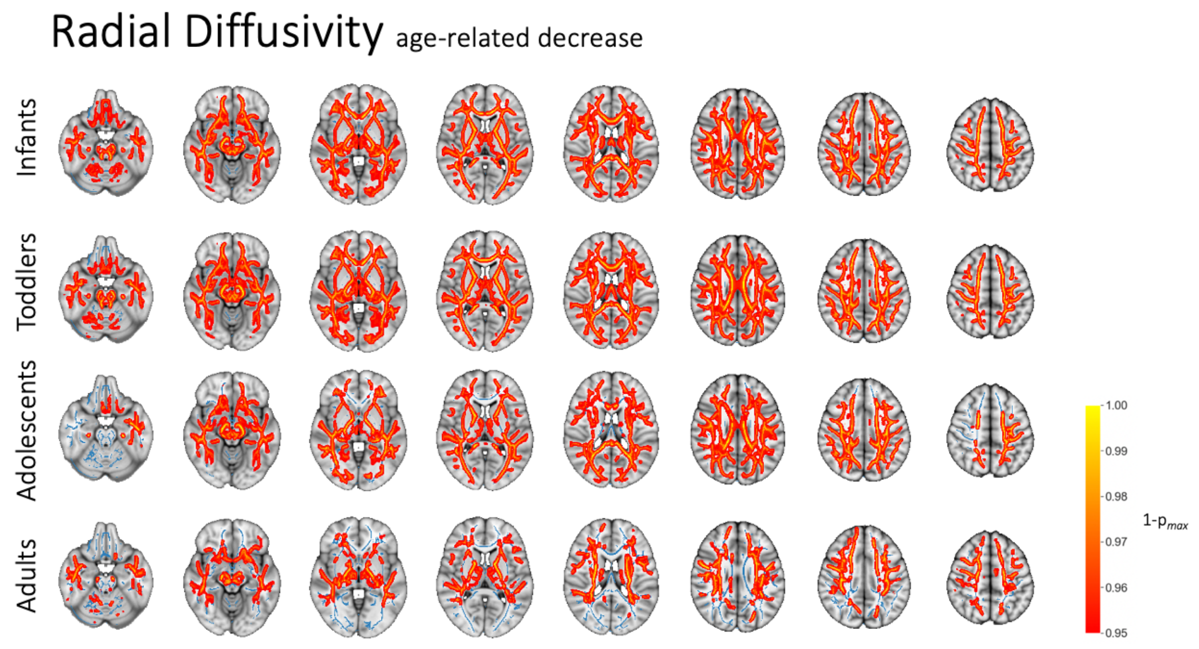

Suppl. Figure 4: Age-related changes in Radial Diffusivity in all four study cohorts. Figures show the MNI152 standard brain template with mean FA skeleton and significant changes ($p<0.05$) as assessed in voxel-wise GLM (using FSL TBSS) in red.

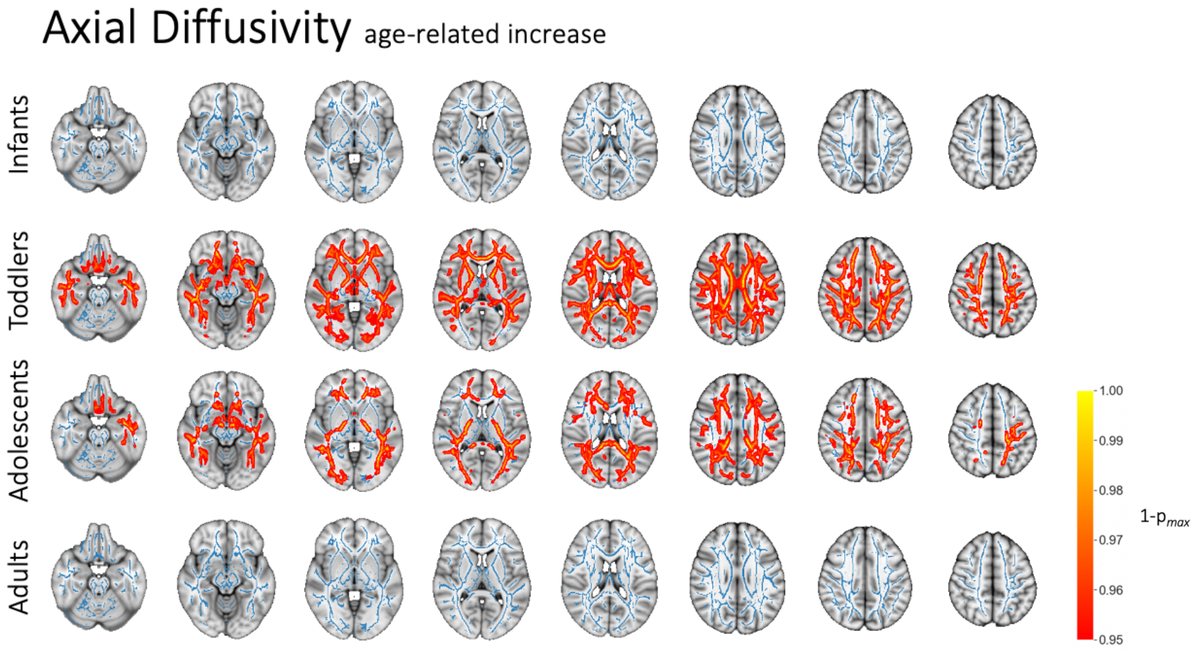

Suppl. Figure 5: Age-related changes in Axial Diffusivity in all four study cohorts. Figures show the MNI152 standard brain template with mean FA skeleton and significant changes ($p<0.05$) as assessed in voxel-wise GLM (using FSL TBSS) in red.

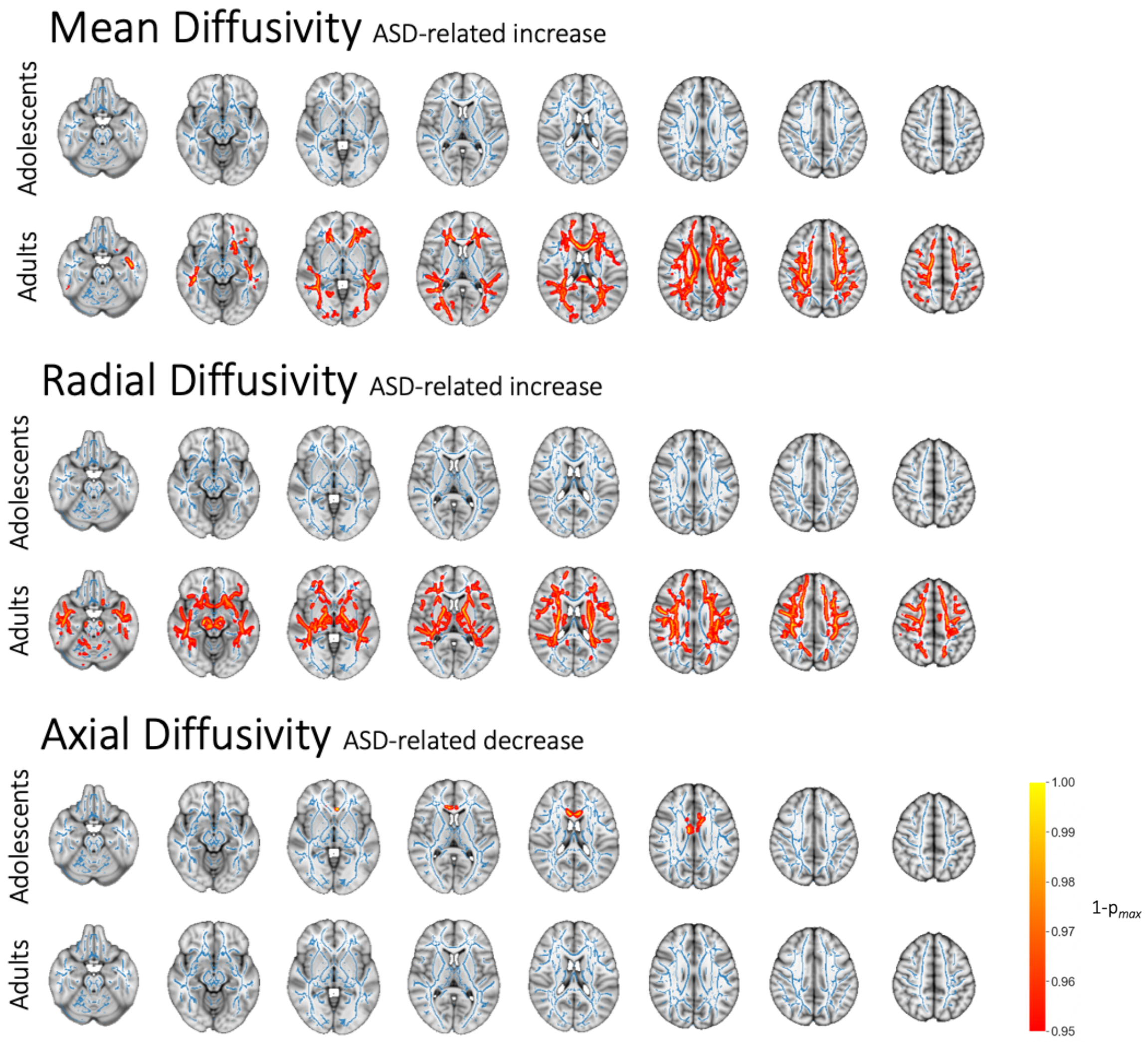

Suppl. Figure 6: ASD-related changes in Mean, Radial and Axial Diffusivity in adolescents and adults. Figures show the MNI152 standard brain template with mean FA skeleton and significant changes ($p<0.05$) as assessed in voxel-wise GLM (using FSL TBSS) in red.

2. List of Atlas Regions

L = Left, R= Right

2.1 John Hopkins University White Matter Label Atlas

Middle cerebellar peduncle
Pontine crossing tract (a part of MCP)
Genu of corpus callosum
Body of corpus callosum
Splenium of corpus callosum
Fornix (column and body of fornix)
Corticospinal tract L/R
Medial lemniscus L/R
Inferior cerebellar peduncle L/R
Superior cerebellar peduncle L/R
Cerebral peduncle L/R
Anterior limb of internal capsule L/R
Posterior limb of internal capsule L/R
Retrolenticular part of internal capsule L/R
Anterior corona radiata L/R
Superior corona radiata L/R
Posterior corona radiata L/R
Posterior thalamic radiation (include optic radiation) L/R
Sagittal stratum (include inferior longitidinal fasciculus and inferior fronto-occipital fasciculus) L/R
External capsule L/R
Cingulum (cingulate gyrus) L/R
Cingulum (hippocampus) L/R
Fornix (cres) / Stria terminalis L/R
Superior longitudinal fasciculus L/R
Superior fronto-occipital fasciculus L/R
Uncinate fasciculus L/R
Tapetum L/R

2.2 Harvard-Oxford Cortical Atlas

Frontal Pole L/R
Insular Cortex L/R
Superior Frontal Gyrus L/R
Middle Frontal Gyrus L/R
Inferior Frontal Gyrus pars triangularis L/R
Inferior Frontal Gyrus pars opercularis L/R
Precentral Gyrus L/R

Temporal Pole L/R
Superior Temporal Gyrus, anterior division L/R
Superior Temporal Gyrus, posterior division L/R
Middle Temporal Gyrus, anterior division L/R
Middle Temporal Gyrus, posterior division L/R
Middle Temporal Gyrus, temporooccipital part L/R
Inferior Temporal Gyrus, anterior division L/R
Inferior Temporal Gyrus, posterior division L/R
Inferior Temporal Gyrus, temporooccipital part L/R
Postcentral Gyrus L/R
Superior Parietal Lobule L/R
Supramarginal Gyrus, anterior division L/R
Supramarginal Gyrus, posterior division L/R
Angular Gyrus L/R
Lateral Occipital Cortex, superior division L/R
Lateral Occipital Cortex, inferior division L/R
Intracalcarine Cortex L/R
Frontal Medial Cortex L/R
Juxtapositional Lobule Cortex L/R
Subcallosal Cortex L/R
Paracingulate Gyrus L/R
Cingulate Gyrus, anterior division L/R
Cingulate Gyrus, posterior division L/R
Precuneous Cortex L/R
Frontal Orbital Cortex L/R
Parahippocampal Gyrus, anterior division L/R
Parahippocampal Gyrus, posterior division L/R
Lingual Gyrus L/R
Temporal Fusiform Cortex, anterior division L/R
Temporal Fusiform Cortex, posterior division L/R
Temporal Occipital Fusiform Cortex L/R
Occipital Fusiform Gyrus L/R
Frontal Operculum Cortex L/R
Central Opercular Cortex L/R
Parietal Operculum Cortex L/R
Planum Polare L/R
Heschl's Gyrus (incl. H1 and H2) L/R
Planum Temporale L/R
Supracalcarine Cortex L/R
Occipital Pole L/R

2.3 Harvard-Oxford Subcortical Atlas

Thalamus L/R
Putamen L/R
Pallidum L/R

Hippocampus L/R
Amygdala L/R
Nucleus Accumbens L/R
Brainstem

3. Machine Learning

The dimensionality reduction and machine learning framework applied here are equivalent to Haider et al.'s work [1]. In the following, descriptions of the algorithms used are replicated below.

3.1 Classifiers

3.1.1 Elastic Net (ElNet)

Elastic Net was implemented using *cv.glmnet* in R *glmnet* package [2], applying the internal 10-fold cross validation mode to determine lambda based on resulting AUCs. Elastic Net is a linear combination of Ridge and Least Absolute Shrinkage and Selection Operator (LASSO) regression. Ridge and LASSO were combined linearly and weighted through the mixing parameter *alpha*, which was tuned in Bayesian Optimization.

3.1.2 Naive Bayes (NBayes)

We used *naive_bayes* in R *naivebayes* package [3]. Gaussian distribution was applied for all features, and no Laplace smoothing was applied. NBayes has no tunable hyperparameters other than feature count, which was tuned in Bayesian Optimization if applicable in respective combinations with feature selection algorithms.

3.1.3 Random Forest (RF)

RF as first described by Breiman [4] was implemented using *randomForest* function of R *randomForest* package [5]. The number of features (*mtry*) at each node as well as the number of terminal nodes in a tree (*maxnodes*) were treated as hyperparameters and tuned in Bayesian Optimization. The total number of trees was set to 1000 and sampling of cases was performed with replacement.

3.1.4 Support Vector Machine (SVM) with sigmoid (SVM sig) and radial (SVM rad) kernel

SVM were implemented using R *e1071* [6] package. In binary classification, SVM aim to identify the optimal separating hyperplane between two groups by maximizing the margins between the classes' closest points. Cost refers to a parameter determining how the number of datapoints in the margin is penalized, hence, how wide the margin is. We applied a sigmoid kernel following the formula $k(x, y) = \tanh(\gamma u^T v + c0)$, where gamma (γ) refers to the slope and *coef0* to the intercept constant [7]. *Gamma, coef0* and *cost* were optimized in Bayesian Optimization.

Another kernel was applied using a radial basis function (b-spline). A b-spline is a piecewise polynomial function of b adjuncted polynomes. In graphical representation, b-splines are flexible bands that pass through a set of defined, so-called control points, and appear as smooth curves. The formula for radial kernels is $k(x, y) = \exp(-\gamma|u - v|^2)$, where u and v refer to the respectively regressions computed to fit the kernel to data [7]. *Gamma* and *cost* were treated as hyperparameters and tuned in Bayesian Optimization.

3.1.5 Extreme Gradient Boosting (XGB)

XGB was implemented using *gbtree* option in R *xgboost* [8] package in tree-booster mode. XGB is an improved implementation of gradient boosting decision trees that uses more accurate approximations to find the best tree in iterative least-squares regression. Contrarily to common gradient boosting, XGB uses advanced regularization and second-order gradients [9]. Specifications of XGB functionality are the learning rate *eta* that reflects on step size shrinkage; the required minimum loss reduction for each further partition *gamma*; the maximum depth of a tree *max_depth*; the minimum weight of each child tree *min_child_weight*; the subsample ratio of training instances applied in every boosting iteration subsample as well as of columns *colsample_by_tree*; and the regularization parameter *lambda*. All of the aforementioned parameters were tuned in Bayesian Optimization. Each classifier was applied to the data on its own as well as in combination with different feature selection algorithms to reduce dimensionality and prevent overfitting.

3.2 Feature Selection Algorithms

3.2.1 Hierarchical Clustering (HClust)

An euclidian distance map was calculated using *dist* in the R *stats* package and subsequently clustered by applying *hclust* (R stats), which uses Ward clustering [10], an agglomerative approach that selects most optimal values for an objective function. The 20 features closest to each other were selected. No Bayesian Optimization was applied to tune feature count.

3.2.2 Minimal Redundancy Maximal Relevance (MRMR)

mRMR function[11] in R *praznik* package [12] was used for MRMR feature selection. MRMR combines maximum relevance feature selection, which considers the correlation of each feature

with the final outcome variable, with minimum redundancy, which is a measure of joint distribution and information gain. For every feature, the mutual information as described by Shannon [13] with all other features is calculated, and the n features of highest correlation to the target variable and lowest mutual information with other features are selected. N was treated as a hyperparameter and tuned in Bayesian Optimization.

3.2.3 Pearson correlation-based redundancy reduction with Mutual Information Maximization filter (pMIM)

Using the R stats package, Pearson correlation between every possible feature pair was determined. *findCorrelation* as specified in the R *caret* package [14] was then applied to find and exclude features of highest correlation. Subsequently, a mutual information filter was introduced using MIM function of the R *mRMR* package. N was treated as a hyperparameter and tuned in Bayesian Optimization.

3.2.4 Principal Component Analysis (PCA)-based feature selection

PCA is used for dimensionality reduction while keeping the variance of data relatively equal to original values. Following PCA-based feature selection as suggested by Song et al. [15], We applied singular value decomposition of the data matrix using *prcomp* in R stats package. We selected the n features contributing most to feature extraction. N was treated as a hyperparameter and optimized in Bayesian Optimization.

3.2.5 Logistical Regression using Ridge regularization (RIDGE)

The *glmnet* function in R *cv.glmnet* [2] package was used. The inherent 10-fold cross validation mode determined the lambda penalty when fitting a Ridge logistic regression to all features. Subsequently, the n features of highest regression coefficient value were selected, and n was treated as a hyperparameter and tuned in Bayesian Optimization.

Additionally, each classifier was applied once without feature selection (noFS), considering metrics of all white matter tracts.

Each Algorithm and Classifier as well as their respective abbreviations and hyperparameters are given in supplementary table 1.

**Machine Learning Classifiers**				
Abbreviation	*Classifier*	*Hyperparameters*	*Upper and Lower Bound*	*Repetitions in Bay. Opt.*
ElNet	Elastic Net	alpha	0 – 1	100
		n (Features)	2 – 30	200
NBayes	Naive Bayes[a]	n (Features)	2 – 30	100
RF	Random Forest	n (Features)	2 – 30	150
		maxnodes	$2 - 2^{15}$	150
		mtry	2 – 40	150
		n (Features)	2 – 30	50
SVM rad	SVM radial	Cost	0.1 – 10	150
		gamma	0 – 0.5	150
		n (Features)	2 – 30	200
SVM sig	SVM sigmoid	Coef0	0 – 1	200
		cost	0.1 – 10	200
		gamma	0 – 0.5	200
		n (Features)	2 – 30	150
XGB	Extreme Gradient Boosting	Eta	0 – 1	200
		Gamma	0 – 5	200
		max_depth	5 – 15	200
		Min_child_weight	0 – 20	200
		Subsample	0.8 – 1	200
		Colsample_by_tree	0.8 – 1	200

		lambda	0.5 – 1	200

**Feature Selection Algorithms**		
Abbreviation	*Algorithm*	*Tuning of n in Bayesian Optimization*
HClust	Hierarchical Clustering	no
MRMR	Minimal Redundancy Maximal Relevance	yes
pMIM	Pearson correlation-based redundancy reduction with Mutual Information Maximization filer	yes
PCA	Principal Component Analysis based feature selection	yes
RIDGE	Logistic Regression using Ridge regularization	yes
no FS	no Feature Selection	n/a

Supplementary table 1: Overview of all Feature Selection and Classifiers used, as well as their hyperparameters and respective boundaries for Bayesian optimization. For Naive Bayes only one parameter as tuned, which will not be tuned in two of the feature selection specifications (HClust and noFS), no Bayesian Optimization has been applied in these combinations.

3.3Final Model Hyperparameters

3.3.1 Final Model in Adolescent and Adult Cohort: Support Vector Machine with Hierarchical Clustering Feature Selection

**Hyperparameter**	**Value**
cost	2.1100
gamma	0.1311

3.3.2 Final Model in all Cohorts: Extreme Gradient Boosting without Feature Selection

**Hyperparameter**	**Value**
eta	0.0583
gamma	3.8748
max_depth	13
min_child_weight	20
subsample	0.9483
colsample_bytree	0.8383

lambda	0.9654

Suppl. Table 2: Tuned Hyperparameters obtained in Bayesian Optimization of the final model.

3.4Feature Importance Calculation

We used *xgb.importance* in the R *xgboost* package to quantify the gain (improvement in accuracy contributed to the branches the respective feature is on) associated with each feature.

#	**Feature**	**Importance score**
1	RD in Pontine crossing tract (a part of MCP)	$9.93x10^{-2}$
2	RD in Left Medial lemniscus	$9.13x10^{-2}$
3	FA in Genu of corpus callosum	$7.24x10^{-2}$
4	AD in Left Uncinate fasciculus	$5.32x10^{-2}$
5	MD in Right Fornix (cres) / Stria terminalis	$3.75x10^{-2}$
6	AD in Right Cingulum (hippocampus)	$3.11x10^{-2}$
7	AD in Right Uncinate fasciculus	$2.59x10^{-2}$
8	AD in Right Posterior corona radiata	$2.52x10^{-2}$
9	AD in Right Tapetum	$2.35x10^{-2}$
10	Sex	$2.33x10^{-2}$
11	MD in Middle cerebellar peduncle	$2.33x10^{-2}$
12	FA in Right Posterior limb of internal capsule	$2.19x10^{-2}$
13	FA in Left Uncinate fasciculus	$1.68x10^{-2}$
14	AD in Left Anterior limb of internal capsule	$1.49x10^{-2}$
15	ED in Left Superior longitudinal fasciculus	$1.46x10^{-2}$
16	AD in Fornix (column and body of fornix)	$1.44x10^{-2}$
17	MD in Right Cingulum (hippocampus)	$1.37x10^{-2}$
18	FA in Body of corpus callosum	$1.32x10^{-2}$
19	ED in Left Superior fronto-occipital fasciculus	$1.22x10^{-2}$
20	ED in Right Anterior limb of internal capsule	$1.2x10^{-2}$

Suppl. Table 3: Feature Importance Calculation of final selected model

4 Tract-Based Multiple Regression

Results of tract-based multiple regression analysis are listed in the following. P-values were adjusted for multiple comparisons using false discovery rate correction.

4.1 Infant Cohort

Fractional Anisotropy	Age		ASD		Sex	
	Coefficient (95% CI)	p-value	Coefficient (95% CI)	p-value	Coefficient (95% CI)	p-value
Middle cerebellar peduncle	1.705(1.034 - 2.376) x10^-2	<0.001	1.42(0.094 - 2.746) x10^-2	0.151	1.091(-0.076 - 2.259) x10^-2	0.400
Pontine crossing tract	1.16(0.532 - 1.787) x10^-2	<0.001	0.42(-0.82 - 1.66) x10^-2	0.633	1.087(-0.005 - 2.178) x10^-2	0.400
Genu of corpus callosum	1.641(0.951 - 2.33) x10^-2	<0.001	1.449(0.087 - 2.81) x10^-2	0.392	-0.505(-1.704 - 0.693) x10^-2	0.727
Body of corpus callosum	0.96(0.22 - 1.7) x10^-2	0.015	0.634(-0.828 - 2.095) x10^-2	0.633	0.282(-1.004 - 1.569) x10^-2	0.827
Splenium of corpus callosum	1.871(1.272 - 2.469) x10^-2	<0.001	0.625(-0.556 - 1.806) x10^-2	0.633	0.607(-0.433 - 1.648) x10^-2	0.571
Fornix	0.948(0.179 - 1.716) x10^-2	0.020	0.057(-1.46 - 1.575) x10^-2	0.938	-0.096(-1.432 - 1.24) x10^-2	0.965
Corticospinal tract R	1.17(0.571 - 1.769) x10^-2	<0.001	0.489(-0.695 - 1.673) x10^-2	0.633	1.896(0.854 - 2.938) x10^-2	<0.001
Corticospinal tract L	1.238(0.715 - 1.762) x10^-2	<0.001	-0.006(-1.04 - 1.028) x10^-2	0.705	1.581(0.67 - 2.491) x10^-2	<0.001
Medial lemniscus R	1.305(0.632 - 1.977) x10^-2	<0.001	-0.129(-1.457 - 1.2) x10^-2	0.934	1.394(0.224 - 2.564) x10^-2	0.400
Medial lemniscus L	1.177(0.606 - 1.748) x10^-2	<0.001	-0.28(-1.408 - 0.847) x10^-2	0.938	0.805(-0.188 - 1.797) x10^-2	0.410
Inferior cerebellar peduncle R	1.504(0.893 - 2.115) x10^-2	<0.001	0.985(-0.222 - 2.192) x10^-2	0.633	0.246(-0.816 - 1.309) x10^-2	0.827
Inferior cerebellar peduncle L	1.086(0.534 - 1.637) x10^-2	<0.001	1.305(0.216 - 2.394) x10^-2	0.176	0.271(-0.688 - 1.23) x10^-2	0.827
Superior cerebellar peduncle R	1.213(0.511 - 1.916) x10^-2	<0.001	0.707(-0.681 - 2.095) x10^-2	0.633	0.291(-0.931 - 1.513) x10^-2	0.827
Superior cerebellar peduncle L	1.267(0.485 - 2.05) x10^-2	0.003	0.937(-0.608 - 2.483) x10^-2	0.633	0.067(-1.294 - 1.427) x10^-2	0.965
Cerebral peduncle R	0.736(0.176 - 1.295) x10^-2	0.014	-0.369(-1.474 - 0.737) x10^-2	0.934	1.502(0.529 - 2.476) x10^-2	0.138
Cerebral peduncle L	0.458(-0.066 - 0.983) x10^-2	0.094	-0.039(-1.075 - 0.997) x10^-2	0.934	0.796(-0.117 - 1.708) x10^-2	0.400
Anterior limb of internal capsule R	1.822(1.189 - 2.456) x10^-2	<0.0011	-0.139(-1.389 - 1.112) x10^-2	0.934	-0.303(-1.404 - 0.798) x10^-2	0.827
Anterior limb of internal capsule L	1.356(0.76 - 1.951) x10^-2	<0.001	0.193(-0.983 - 1.369) x10^-2	0.934	-0.186(-1.221 - 0.85) x10^-2	0.875
Posterior limb of internal capsule R	0.876(0.259 - 1.492) x10^-2	0.009	0.134(-1.084 - 1.351) x10^-2	0.919	1.017(-0.054 - 2.089) x10^-2	0.400
Posterior limb of internal capsule L	0.984(0.286 - 1.683) x10^-2	0.009	0.334(-1.046 - 1.713) x10^-2	0.934	-1.158(-2.372 - 0.057) x10^-2	0.400
Retrolenticular part of internal capsule R	0.494(-0.049 - 1.037) x10^-2	0.086	-0.905(-1.977 - 0.168) x10^-2	0.705	0.83(-0.114 - 1.774) x10^-2	0.400
Retrolenticular part of internal capsule L	0.926(0.407 - 1.445) x10^-2	<0.001	-0.778(-1.803 - 0.246) x10^-2	0.705	0.2(-0.703 - 1.102) x10^-2	0.827
Anterior corona radiata R	0.963(0.46 - 1.465) x10^-2	<0.001	0.68(-0.312 - 1.672) x10^-2	0.633	-0.705(-1.578 - 0.168) x10^-2	0.410
Anterior corona radiata L	0.868(0.4 - 1.336) x10^-2	<0.001	-0.112(-1.037 - 0.812) x10^-2	0.934	-0.65(-1.464 - 0.163) x10^-2	0.410
Superior corona radiata R	1.186(0.614 - 1.758) x10^-2	<0.001	0.197(-0.932 - 1.326) x10^-2	0.934	-0.554(-1.549 - 0.44) x10^-2	0.571
Superior corona radiata L	1.208(0.635 - 1.781) x10^-2	<0.001	0.198(-0.933 - 1.33) x10^-2	0.934	-0.418(-1.414 - 0.578) x10^-2	0.727
Posterior corona radiata R	0.788(0.303 - 1.272) x10^-2	0.003	-0.106(-1.062 - 0.85) x10^-2	0.934	0.339(-0.503 - 1.181) x10^-2	0.727
Posterior corona radiata L	0.661(0.147 - 1.174) x10^-2	0.016	-0.865(-1.879 - 0.149) x10^-2	0.633	0.048(-0.844 - 0.94) x10^-2	0.965
Posterior thalamic radiation R	1.046(0.545 - 1.547) x10^-2	<0.001	-0.806(-1.796 - 0.183) x10^-2	0.633	-0.512(-1.383 - 0.359) x10^-2	0.571
Posterior thalamic radiation L	1.185(0.683 - 1.687) x10^-2	<0.001	-0.703(-1.695 - 0.289) x10^-2	0.838	-0.207(-1.081 - 0.667) x10^-2	0.827
Sagittal stratum R	0.926(0.425 - 1.427) x10^-2	<0.001	-1.15(-2.141 - -0.16) x10^-2	0.633	0.655(-0.216 - 1.527) x10^-2	0.432
Sagittal stratum L	0.482(-0.034 - 0.998) x10^-2	0.080	-1.091(-2.11 - -0.071) x10^-2	0.460	-0.079(-0.976 - 0.819) x10^-2	0.965
External capsule R	0.881(0.407 - 1.354) x10^-2	<0.001	0.064(-0.871 - 1) x10^-2	0.934	-0.066(-0.89 - 0.758) x10^-2	0.965
External capsule L	0.827(0.395 - 1.258) x10^-2	<0.001	-0.117(-0.969 - 0.736) x10^-2	0.934	-0.426(-1.177 - 0.324) x10^-2	0.571
Cingulum cingulate gyrus R	1.241(0.762 - 1.721) x10^-2	<0.001	0.009(-0.938 - 0.957) x10^-2	0.879	0.519(-0.315 - 1.353) x10^-2	0.565
Cingulum cingulate gyrus L	1.418(0.919 - 1.917) x10^-2	<0.001	0.453(-0.533 - 1.438) x10^-2	0.633	0.573(-0.295 - 1.44) x10^-2	0.525
Cingulum hippocampus R	0.642(-0.064 - 1.349) x10^-2	0.086	0.177(-1.219 - 1.574) x10^-2	0.934	0.02(-1.21 - 1.249) x10^-2	0.975
Cingulum hippocampus L	0.59(-0.113 - 1.294) x10^-2	0.106	-0.459(-1.848 - 0.931) x10^-2	0.817	-0.332(-1.555 - 0.892) x10^-2	0.827
Fornix Stria terminalis R	0.764(0.199 - 1.329) x10^-2	0.011	-0.359(-1.476 - 0.757) x10^-2	0.934	0.287(-0.696 - 1.27) x10^-2	0.827
Fornix Stria terminalis. L	0.211(-0.465 - 0.888) x10^-2	0.538	-0.419(-1.756 - 0.917) x10^-2	0.707	-0.881(-2.058 - 0.296) x10^-2	0.432
Superior longitudinal fasciculus R	1.209(0.776 - 1.641) x10^-2	<0.001	0.058(-0.796 - 0.913) x10^-2	0.934	-0.307(-1.059 - 0.445) x10^-2	0.727
Superior longitudinal fasciculus L	1.242(0.809 - 1.676) x10^-2	<0.001	0.056(-0.801 - 0.912) x10^-2	0.934	0.053(-0.701 - 0.807) x10^-2	0.965
Superior fronto.occipital fasciculus R	1.57(0.745 - 2.394) x10^-2	<0.001	-0.002(-1.63 - 1.627) x10^-2	0.938	-1.257(-2.691 - 0.176) x10^-2	0.400
Superior fronto.occipital fasciculus L	1.199(0.411 - 1.987) x10^-2	0.005	1.036(-0.52 - 2.593) x10^-2	0.633	-0.947(-2.318 - 0.423) x10^-2	0.500
Uncinate fasciculus R	0.778(-0.095 - 1.652) x10^-2	0.089	-0.965(-2.69 - 0.76) x10^-2	0.879	0.489(-1.03 - 2.008) x10^-2	0.827
Uncinate fasciculus L	0.391(-0.49 - 1.272) x10^-2	0.390	1.455(-0.284 - 3.195) x10^-2	0.633	0.028(-1.504 - 1.56) x10^-2	0.975
Tapetum R	0.616(-0.424 - 1.656) x10^-2	0.255	0.935(-1.119 - 2.99) x10^-2	0.934	-1.867(-3.676 - -0.058) x10^-2	0.400
Tapetum L	0.969(-0.055 - 1.993) x10^-2	0.079	-0.179(-2.202 - 1.843) x10^-2	0.934	-0.934(-2.714 - 0.847) x10^-2	0.604

Mean Diffusivity	Age		ASD		Sex	
	Coefficient (95% CI)	p-value	Coefficient (95% CI)	p-value	Coefficient (95% CI)	p-value
Middle cerebellar peduncle	-0.576(-1.356 - 0.203) x10^-5	0.171	0.442(-1.098 - 1.982) x10^-5	0.064	0.498(-0.857 - 1.854) x10^-5	0.855
Pontine crossing tract	-1.264(-2.714 - 0.185) x10^5	0.110	-0.969(-3.832 - 1.895) x10^-5	0.302	-1.608(-4.129 - 0.913) x10^-5	0.814
Genu of corpus callosum	-3.255(-4.447 - -2.064) x10^-5	<0.001	-1.809(-4.162 - 0.544) x10^-5	0.062	0.518(-1.554 - 2.589) x10^-5	0.933
Body of corpus callosum	-2.462(-3.592 - -1.332) x10^-5	<0.001	-0.867(-3.099 - 1.364) x10^-5	0.062	0.401(-1.564 - 2.365) x10^-5	0.944
Splenium of corpus callosum	-4.105(-5.082 - -3.129) x10^-5	<0.001	0.159(-1.771 - 2.088) x10^-5	0.097	0.5(-1.198 - 2.199) x10^-5	0.869
Fornix	-5.282(-9.67 - -0.894) x10^-5	0.026	-2.757(-11.424 - 5.911) x10^-5	0.747	6.726(-0.905 - 14.358) x10^-5	0.814
Corticospinal tract R	-0.279(-1.569 - 1.011) x10^-5	0.699	-0.245(-2.793 - 2.303) x10^-5	0.618	0.342(-1.902 - 2.585) x10^-5	0.975
Corticospinal tract L	-0.39(-1.436 - 0.657) x10^-5	0.517	0.192(-1.876 - 2.26) x10^-5	0.088	1.061(-0.759 - 2.882) x10^-5	0.814
Medial lemniscus R	-1.085(-5.184 - 3.014) x10^-5	0.657	5.417(-2.679 - 13.513) x10^-5	0.302	-2.446(-9.574 - 4.682) x10^-5	0.855
Medial lemniscus L	-0.73(-4.852 - 3.391) x10^-5	0.742	7.925(-0.216 - 16.067) x10^-5	0.064	-0.957(-8.125 - 6.211) x10^-5	0.975

Inferior cerebellar peduncle R	-0.089(-1.289 - 1.11) x10^-5	0.883	-0.18(-2.55 - 2.19) x10^-5	0.111	0.997(-1.09 - 3.083) x10^-5	0.814
Inferior cerebellar peduncle L	1.249(-0.312 - 2.809) x10^-5	0.143	2.015(-1.067 - 5.098) x10^-5	0.064	1.426(-1.288 - 4.139) x10^-5	0.814
Superior cerebellar peduncle R	-0.646(-1.895 - 0.604) x10^-5	0.353	3.235(0.767 - 5.704) x10^-5	0.896	-1.516(-3.689 - 0.658) x10^-5	0.814
Superior cerebellar peduncle L	-0.333(-1.749 - 1.083) x10^-5	0.686	1.431(-1.367 - 4.228) x10^-5	0.596	0.229(-2.234 - 2.692) x10^-5	0.975
Cerebral peduncle R	-0.785(-1.804 - 0.233) x10^-5	0.156	-0.572(-2.584 - 1.44) x10^-5	0.055	0.384(-1.388 - 2.155) x10^-5	0.944
Cerebral peduncle L	-1.303(-2.229 - -0.378) x10^-5	0.009	0.351(-1.477 - 2.179) x10^-5	0.055	-1.187(-2.796 - 0.423) x10^-5	0.814
Anterior limb of internal capsule R	-2.511(-3.197 - -1.826) x10^-5	<0.001	0.133(-1.222 - 1.487) x10^-5	0.061	-0.482(-1.675 - 0.711) x10^-5	0.852
Anterior limb of internal capsule L	-2.541(-3.271 - -1.812) x10^-5	<0.001	-0.419(-1.86 - 1.022) x10^-5	0.061	-0.071(-1.34 - 1.198) x10^-5	0.975
Posterior limb of internal capsule R	-1.628(-2.317 - -0.938) x10^-5	<0.001	0.319(-1.043 - 1.681) x10^-5	0.055	0.439(-0.76 - 1.638) x10^-5	0.855
Posterior limb of internal capsule L	-2.001(-2.767 - -1.236) x10^-5	<0.001	0.435(-1.078 - 1.948) x10^-5	0.064	0.634(-0.698 - 1.966) x10^-5	0.814
Retrolenticular part of internal capsule R	-1.599(-2.311 - -0.886) x10^-5	<0.001	-0.269(-1.676 - 1.138) x10^-5	0.055	-0.689(-1.928 - 0.55) x10^-5	0.814
Retrolenticular part of internal capsule L	-1.738(-2.476 - -1.001) x10^-5	<0.001	-0.361(-1.817 - 1.096) x10^-5	0.068	0.593(-0.689 - 1.876) x10^-5	0.814
Anterior corona radiata R	-2.718(-3.449 - -1.988) x10^-5	<0.001	0.284(-1.159 - 1.726) x10^-5	0.062	-0.875(-2.145 - 0.395) x10^-5	0.814
Anterior corona radiata L	-2.808(-3.558 - -2.058) x10^-5	<0.001	0.259(-1.223 - 1.74) x10^-5	0.062	-0.718(-2.023 - 0.586) x10^-5	0.814
Superior corona radiata R	-2.859(-3.628 - -2.091) x10^-5	<0.001	0.181(-1.338 - 1.699) x10^-5	0.055	0.137(-1.2 - 1.474) x10^-5	0.975
Superior corona radiata L	-2.614(-3.413 - -1.816) x10^-5	<0.001	-0.011(-1.588 - 1.566) x10^-5	0.062	0.171(-1.218 - 1.559) x10^-5	0.975
Posterior corona radiata R	-2.471(-3.45 - -1.492) x10^-5	<0.001	0.207(-1.727 - 2.141) x10^-5	0.055	-0.001(-1.704 - 1.702) x10^-5	0.999
Posterior corona radiata L	-1.959(-2.935 - -0.983) x10^-5	<0.001	0.281(-1.647 - 2.209) x10^-5	0.064	0.768(-0.929 - 2.466) x10^-5	0.814
Posterior thalamic radiation R	-2.154(-3.081 - -1.228) x10^-5	<0.001	0.321(-1.508 - 2.151) x10^-5	0.066	0.879(-0.732 - 2.49) x10^-5	0.814
Posterior thalamic radiation L	-2.638(-3.632 - -1.644) x10^-5	<0.001	0.284(-1.68 - 2.247) x10^-5	0.079	1.23(-0.498 - 2.959) x10^-5	0.814
Sagittal stratum R	-1.546(-2.367 - -0.725) x10^-5	<0.001	1.488(-0.134 - 3.109) x10^-5	0.098	-0.078(-1.506 - 1.35) x10^-5	0.975
Sagittal stratum L	-1.95(-2.824 - -1.075) x10^-5	<0.001	0.088(-1.639 - 1.816) x10^-5	0.062	0.841(-0.68 - 2.362) x10^-5	0.814
External capsule R	-2.051(-2.568 - -1.535) x10^-5	<0.001	0.512(-0.509 - 1.533) x10^-5	0.055	-0.295(-1.194 - 0.603) x10^-5	0.856
External capsule L	-2.049(-2.603 - -1.495) x10^-5	<0.001	0.064(-1.03 - 1.158) x10^-5	0.062	0.993(0.03 - 1.957) x10^-5	0.814
Cingulum cingulate gyrus R	-2.081(-2.816 - -1.345) x10^-5	<0.001	0.265(-1.188 - 1.719) x10^-5	0.062	0.662(-0.617 - 1.942) x10^-5	0.814
Cingulum cingulate gyrus L	-1.933(-2.653 - -1.213) x10^-5	<0.001	0.389(-1.033 - 1.811) x10^-5	0.066	0.001(-1.251 - 1.253) x10^-5	0.999
Cingulum hippocampus R	-1.025(-2.014 - -0.036) x10^-5	0.054	0.406(-1.548 - 2.359) x10^-5	0.055	1.116(-0.604 - 2.836) x10^-5	0.814
Cingulum hippocampus L	-1.694(-2.586 - -0.801) x10^-5	<0.001	1.179(-0.584 - 2.942) x10^-5	0.097	-0.181(-1.733 - 1.371) x10^-5	0.975
Fornix Stria terminalis R	-1.511(-2.264 - -0.758) x10^-5	<0.001	-0.352(-1.839 - 1.136) x10^-5	0.621	0.3(-1.01 - 1.609) x10^-5	0.944
Fornix Stria terminalis. L	-1.533(-2.375 - -0.69) x10^-5	<0.001	0.411(-1.253 - 2.076) x10^-5	0.183	1.28(-0.186 - 2.745) x10^-5	0.814
Superior longitudinal fasciculus R	-3.076(-3.807 - -2.345) x10^-5	<0.001	-0.525(-1.968 - 0.919) x10^-5	0.062	0.783(-0.488 - 2.053) x10^-5	0.814
Superior longitudinal fasciculus L	-2.834(-3.619 - -2.05) x10^-5	<0.001	0.071(-1.479 - 1.621) x10^-5	0.256	0.582(-0.783 - 1.946) x10^-5	0.837
Superior fronto.occipital fasciculus R	-2.715(-3.64 - -1.791) x10^-5	<0.001	0.698(-1.129 - 2.525) x10^-5	0.055	-0.052(-1.66 - 1.556) x10^-5	0.990
Superior fronto.occipital fasciculus L	-2.66(-3.613 - -1.708) x10^-5	<0.001	0.387(-1.494 - 2.268) x10^-5	0.113	-0.1(-1.756 - 1.557) x10^-5	0.975
Uncinate fasciculus R	-1.61(-2.89 - -0.329) x10^-5	0.020	0.086(-2.443 - 2.615) x10^-5	0.055	0.365(-1.861 - 2.591) x10^-5	0.975
Uncinate fasciculus L	-1.118(-2.127 - -0.11) x10^-5	0.040	-2.104(-4.095 - -0.112) x10^-5	0.062	1.951(0.198 - 3.704) x10^-5	0.814
Tapetum R	-5.59(-9.536 - -1.644) x10^-5	0.009	-3.16(-10.954 - 4.634) x10^-5	0.062	2.439(-4.423 - 9.301) x10^-5	0.855
Tapetum L	-5.925(-10.772 - -1.079) x10^-5	0.024	-2.222(-11.794 - 7.351) x10^-5	0.321	2.626(-5.802 - 11.054) x10^-5	0.862

**Radial Diffusivity**	**Age**		**ASD**		**Sex**	
	**Coefficient (95% CI)**	**p-value**	**Coefficient (95% CI)**	**p-value**	**Coefficient (95% CI)**	**p-value**
Middle cerebellar peduncle	-1.488(-2.285 - -0.69) x10^-5	<0.001	-0.387(-1.963 - 1.188) x10^-5	0.996	-0.392(-1.779 - 0.995) x10^-5	0.904
Pontine crossing tract	-1.562(-2.694 - -0.429) x10^-5	0.010	-0.669(-2.906 - 1.567) x10^-5	0.996	-1.647(-3.616 - 0.322) x10^-5	0.748
Genu of corpus callosum	-3.66(-4.966 - -2.355) x10^-5	<0.001	-2.465(-5.044 - 0.114) x10^-5	0.996	0.856(-1.415 - 3.126) x10^-5	0.904
Body of corpus callosum	-2.624(-3.981 - -1.266) x10^-5	<0.001	-1.153(-3.835 - 1.528) x10^-5	0.996	-0.106(-2.466 - 2.255) x10^-5	0.971
Splenium of corpus callosum	-4.115(-5.136 - -3.094) x10^-5	<0.001	-0.28(-2.297 - 1.737) x10^-5	0.996	-0.33(-2.105 - 1.446) x10^-5	0.904
Fornix	-4.74(-8.759 - -0.721) x10^-5	0.027	-1.706(-9.645 - 6.232) x10^-5	0.996	5.526(-1.463 - 12.515) x10^-5	0.754
Corticospinal tract R	-0.964(-1.985 - 0.056) x10^-5	0.070	-0.352(-2.367 - 1.664) x10^-5	0.996	-0.926(-2.701 - 0.848) x10^-5	0.904
Corticospinal tract L	-1.129(-2.011 - -0.247) x10^-5	0.016	0.255(-1.487 - 1.996) x10^-5	0.996	-0.232(-1.765 - 1.301) x10^-5	0.904
Medial lemniscus R	-1.481(-4.865 - 1.904) x10^-5	0.406	4.232(-2.453 - 10.918) x10^-5	0.996	-2.764(-8.65 - 3.122) x10^-5	0.904
Medial lemniscus L	-1.139(-4.467 - 2.188) x10^-5	0.500	6.279(-0.294 - 12.851) x10^-5	0.996	-1.127(-6.914 - 4.66) x10^-5	0.904
Inferior cerebellar peduncle R	-0.867(-1.911 - 0.177) x10^-5	0.110	-0.637(-2.7 - 1.426) x10^-5	0.996	0.634(-1.183 - 2.45) x10^-5	0.904
Inferior cerebellar peduncle L	0.452(-0.827 - 1.73) x10^-5	0.496	0.818(-1.708 - 3.343) x10^-5	0.996	0.934(-1.289 - 3.158) x10^-5	0.904
Superior cerebellar peduncle R	-1.152(-2.149 - -0.155) x10^-5	0.029	1.81(-0.159 - 3.779) x10^-5	0.996	-1.155(-2.888 - 0.578) x10^-5	0.904
Superior cerebellar peduncle L	-1.119(-2.273 - 0.034) x10^-5	0.064	0.178(-2.1 - 2.456) x10^-5	0.996	0.205(-1.801 - 2.211) x10^-5	0.948
Cerebral peduncle R	-1.152(-2.161 - -0.142) x10^-5	0.030	-0.116(-2.11 - 1.878) x10^-5	0.996	-0.975(-2.73 - 0.781) x10^-5	0.904
Cerebral peduncle L	-1.291(-2.197 - -0.386) x10^-5	0.008	0.153(-1.635 - 1.941) x10^-5	0.996	-1.409(-2.983 - 0.166) x10^-5	0.748
Anterior limb of internal capsule R	-2.965(-3.725 - -2.205) x10^-5	<0.001	0.335(-1.166 - 1.836) x10^-5	1.000	-0.214(-1.535 - 1.108) x10^-5	0.904
Anterior limb of internal capsule L	-2.746(-3.561 - -1.931) x10^-5	<0.001	-0.267(-1.877 - 1.343) x10^-5	0.996	0.065(-1.353 - 1.482) x10^-5	0.971
Posterior limb of internal capsule R	-1.627(-2.446 - -0.807) x10^-5	<0.001	0.195(-1.423 - 1.813) x10^-5	0.996	-0.557(-1.981 - 0.868) x10^-5	0.904
Posterior limb of internal capsule L	-1.848(-2.773 - -0.924) x10^-5	<0.001	0.086(-1.74 - 1.912) x10^-5	0.996	1.355(-0.252 - 2.963) x10^-5	0.748
Retrolenticular part of internal capsule R	-1.462(-2.201 - -0.723) x10^-5	<0.001	0.604(-0.856 - 2.063) x10^-5	0.996	-1.212(-2.497 - 0.073) x10^-5	0.748
Retrolenticular part of internal capsule L	-1.906(-2.645 - -1.167) x10^-5	<0.001	0.382(-1.077 - 1.841) x10^-5	0.996	0.21(-1.075 - 1.494) x10^-5	0.904
Anterior corona radiata R	-2.807(-3.66 - -1.954) x10^-5	<0.001	-0.259(-1.945 - 1.426) x10^-5	0.996	-0.279(-1.763 - 1.205) x10^-5	0.904
Anterior corona radiata L	-2.835(-3.683 - -1.986) x10^-5	<0.001	0.328(-1.347 - 2.004) x10^-5	0.996	-0.129(-1.604 - 1.346) x10^-5	0.949
Superior corona radiata R	-2.855(-3.732 - -1.978) x10^-5	<0.001	0.082(-1.651 - 1.815) x10^-5	0.996	0.414(-1.112 - 1.94) x10^-5	0.904
Superior corona radiata L	-2.78(-3.7 - -1.861) x10^-5	<0.001	-0.031(-1.847 - 1.786) x10^-5	0.996	0.424(-1.175 - 2.024) x10^-5	0.904
Posterior corona radiata R	-2.548(-3.549 - -1.548) x10^-5	<0.001	0.264(-1.712 - 2.241) x10^-5	0.996	-0.245(-1.985 - 1.495) x10^-5	0.904
Posterior corona radiata L	-2.006(-3.045 - -0.966) x10^-5	<0.001	0.939(-1.114 - 2.993) x10^-5	0.996	0.568(-1.24 - 2.375) x10^-5	0.904
Posterior thalamic radiation R	-2.372(-3.317 - -1.426) x10^-5	<0.001	0.866(-1.002 - 2.734) x10^-5	0.996	0.971(-0.673 - 2.616) x10^-5	0.904
Posterior thalamic radiation L	-2.836(-3.828 - -1.844) x10^-5	<0.001	0.917(-1.041 - 2.876) x10^-5	0.996	1.058(-0.667 - 2.782) x10^-5	0.904
Sagittal stratum R	-1.789(-2.668 - -0.91) x10^-5	<0.001	2.225(0.488 - 3.961) x10^-5	0.996	-0.669(-2.197 - 0.86) x10^-5	0.904
Sagittal stratum L	-1.907(-2.793 - -1.021) x10^-5	<0.001	1.041(-0.71 - 2.791) x10^-5	0.996	0.564(-0.977 - 2.105) x10^-5	0.904
External capsule R	-2.158(-2.773 - -1.544) x10^-5	<0.001	0.503(-0.711 - 1.716) x10^-5	0.996	-0.255(-1.324 - 0.813) x10^-5	0.904
External capsule L	-2.118(-2.743 - -1.494) x10^-5	<0.001	0.211(-1.023 - 1.445) x10^-5	0.996	1(-0.086 - 2.086) x10^-5	0.748
Cingulum cingulate gyrus R	-2.47(-3.197 - -1.743) x10^-5	<0.001	0.19(-1.246 - 1.626) x10^-5	0.996	0.288(-0.976 - 1.552) x10^-5	0.904
Cingulum cingulate gyrus L	-2.468(-3.203 - -1.732) x10^-5	<0.001	0.162(-1.29 - 1.614) x10^-5	0.996	-0.332(-1.61 - 0.947) x10^-5	0.904
Cingulum hippocampus R	-1.188(-2.212 - -0.163) x10^-5	0.028	0.344(-1.679 - 2.368) x10^-5	0.996	0.756(-1.026 - 2.538) x10^-5	0.904
Cingulum hippocampus L	-1.642(-2.58 - -0.704) x10^-5	<0.001	1.388(-0.465 - 3.241) x10^-5	0.996	-0.053(-1.684 - 1.579) x10^-5	0.971
Fornix Stria terminalis R	-1.607(-2.382 - -0.832) x10^-5	<0.001	0.198(-1.333 - 1.729) x10^-5	0.996	-0.001(-1.349 - 1.347) x10^-5	0.999
Fornix Stria terminalis. L	-1.221(-2.159 - -0.284) x10^-5	0.015	0.722(-1.131 - 2.574) x10^-5	0.996	1.357(-0.274 - 2.988) x10^-5	0.748
Superior longitudinal fasciculus R	-3.249(-4.003 - -2.496) x10^-5	<0.001	-0.406(-1.894 - 1.082) x10^-5	0.996	0.805(-0.504 - 2.115) x10^-5	0.904
Superior longitudinal fasciculus L	-3.036(-3.839 - -2.232) x10^-5	<0.001	-0.101(-1.688 - 1.487) x10^-5	0.996	0.417(-0.981 - 1.815) x10^-5	0.904
Superior fronto.occipital fasciculus R	-2.986(-4.041 - -1.931) x10^-5	<0.001	0.576(-1.508 - 2.66) x10^-5	0.996	0.724(-1.111 - 2.558) x10^-5	0.904
Superior fronto.occipital fasciculus L	-2.767(-3.851 - -1.683) x10^-5	<0.001	-0.302(-2.442 - 1.839) x10^-5	0.996	0.566(-1.319 - 2.45) x10^-5	0.904

Uncinate fasciculus R	-1.736(-2.902 - -0.569) x10^-5	0.006	1.013(-1.291 - 3.317) x10^-5	0.996	-0.139(-2.167 - 1.89) x10^-5	0.904
Uncinate fasciculus L	-1.113(-2.151 - -0.076) x10^-5	0.041	-2.005(-4.054 - 0.044) x10^-5	0.996	1.199(-0.605 - 3.002) x10^-5	0.748
Tapetum R	-4.695(-8.184 - -1.205) x10^-5	0.013	-3.511(-10.403 - 3.381) x10^-5	0.996	3.878(-2.19 - 9.946) x10^-5	0.904
Tapetum L	-5.311(-9.374 - -1.249) x10^-5	0.015	-1.673(-9.698 - 6.351) x10^-5	0.996	2.66(-4.405 - 9.725) x10^-5	0.971

**Axial Diffusivity**	**Age**		**ASD**		**Sex**	
	**Coefficient (95% CI)**	**p-value**	**Coefficient (95% CI)**	**p-value**	**Coefficient (95% CI)**	**p-value**
Middle cerebellar peduncle	1.595(0.313 - 2.878) x10^-5	0.026	2.054(-0.48 - 4.587) x10^-5	0.763	2.323(0.092 - 4.553) x10^-5	0.251
Pontine crossing tract	-0.777(-2.849 - 1.295) x10^-5	0.526	-1.169(-5.261 - 2.924) x10^-5	0.823	-1.311(-4.915 - 2.292) x10^-5	0.379
Genu of corpus callosum	-2.124(-3.844 - -0.404) x10^-5	0.026	-2.683(-6.081 - 0.715) x10^-5	0.763	-1.331(-4.323 - 1.66) x10^-5	0.132
Body of corpus callosum	-2.315(-3.807 - -0.823) x10^-5	0.006	-1.231(-4.179 - 1.716) x10^-5	0.876	0.575(-2.02 - 3.17) x10^-5	0.658
Splenium of corpus callosum	-4.505(-5.9 - -3.111) x10^-5	<0.001	0.416(-2.338 - 3.17) x10^-5	0.876	2.271(-0.154 - 4.696) x10^-5	0.590
Fornix	-8.472(-12.539 - -4.404) x10^-5	<0.001	-5.547(-13.581 - 2.488) x10^-5	0.876	8.204(1.131 - 15.278) x10^-5	0.757
Corticospinal tract R	1.607(-0.346 - 3.561) x10^-5	0.164	-0.15(-4.009 - 3.708) x10^-5	0.906	2.712(-0.685 - 6.109) x10^-5	0.288
Corticospinal tract L	1.013(-0.874 - 2.9) x10^-5	0.357	0.891(-2.836 - 4.618) x10^-5	0.763	4.275(0.994 - 7.557) x10^-5	0.221
Medial lemniscus R	-2.126(-7.708 - 3.455) x10^-5	0.526	5.04(-5.984 - 16.065) x10^-5	0.876	-4.406(-14.112 - 5.3) x10^-5	0.590
Medial lemniscus L	-0.124(-6.635 - 6.386) x10^-5	0.970	8.987(-3.873 - 21.847) x10^-5	0.763	-0.076(-11.398 - 11.246) x10^-5	0.998
Inferior cerebellar peduncle R	1.735(-0.451 - 3.921) x10^-5	0.179	0.027(-4.291 - 4.344) x10^-5	0.876	3.914(0.112 - 7.715) x10^-5	0.251
Inferior cerebellar peduncle L	1.482(-0.936 - 3.901) x10^-5	0.313	3.18(-1.597 - 7.957) x10^-5	0.763	3.589(-0.616 - 7.795) x10^-5	0.347
Superior cerebellar peduncle R	-1.207(-4.769 - 2.355) x10^-5	0.560	6.751(-0.284 - 13.786) x10^-5	0.763	-6.359(-12.553 - -0.165) x10^-5	0.251
Superior cerebellar peduncle L	0.593(-3.034 - 4.22) x10^-5	0.763	3.71(-3.454 - 10.873) x10^-5	0.763	0.378(-5.929 - 6.685) x10^-5	0.974
Cerebral peduncle R	0.76(-0.744 - 2.264) x10^-5	0.384	-0.397(-3.367 - 2.573) x10^-5	0.876	4.025(1.409 - 6.64) x10^-5	0.112
Cerebral peduncle L	-0.453(-1.837 - 0.931) x10^-5	0.560	2.92(0.187 - 5.653) x10^-5	0.763	-0.863(-3.27 - 1.543) x10^-5	0.658
Anterior limb of internal capsule R	-2.38(-3.547 - -1.214) x10^-5	<0.001	-0.361(-2.665 - 1.944) x10^-5	0.876	-0.979(-3.008 - 1.05) x10^-5	0.590
Anterior limb of internal capsule L	-2.712(-3.86 - -1.564) x10^-5	<0.001	-1.163(-3.431 - 1.105) x10^-5	0.763	-0.667(-2.663 - 1.33) x10^-5	0.672
Posterior limb of internal capsule R	-1.763(-2.9 - -0.626) x10^-5	0.006	0.262(-1.984 - 2.509) x10^-5	0.876	2.803(0.825 - 4.781) x10^-5	0.112
Posterior limb of internal capsule L	-2.809(-4.243 - -1.376) x10^-5	<0.001	1.174(-1.658 - 4.005) x10^-5	0.918	-1.539(-4.032 - 0.954) x10^-5	0.512
Retrolenticular part of internal capsule R	-2.05(-3.345 - -0.756) x10^-5	0.005	-2.172(-4.729 - 0.385) x10^-5	0.763	0.946(-1.306 - 3.197) x10^-5	0.593
Retrolenticular part of internal capsule L	-1.774(-3.003 - -0.544) x10^-5	0.010	-1.927(-4.355 - 0.501) x10^-5	0.763	1.207(-0.93 - 3.345) x10^-5	0.580
Anterior corona radiata R	-2.722(-3.609 - -1.836) x10^-5	<0.001	0.884(-0.868 - 2.635) x10^-5	0.998	-2.147(-3.689 - -0.605) x10^-5	0.112
Anterior corona radiata L	-3.111(-4.054 - -2.167) x10^-5	<0.001	-0.667(-2.531 - 1.196) x10^-5	0.763	-1.495(-3.136 - 0.145) x10^-5	0.296
Superior corona radiata R	-2.752(-3.68 - -1.824) x10^-5	<0.001	0.24(-1.592 - 2.073) x10^-5	0.976	-0.507(-2.121 - 1.107) x10^-5	0.672
Superior corona radiata L	-2.001(-2.904 - -1.099) x10^-5	<0.001	0.054(-1.728 - 1.837) x10^-5	0.935	-0.438(-2.007 - 1.131) x10^-5	0.681
Posterior corona radiata R	-2.085(-3.219 - -0.951) x10^-5	<0.001	1.134(-1.105 - 3.374) x10^-5	0.876	0.002(-1.969 - 1.974) x10^-5	0.998
Posterior corona radiata L	-1.681(-2.689 - -0.672) x10^-5	0.003	-0.858(-2.85 - 1.133) x10^-5	0.876	1.334(-0.419 - 3.088) x10^-5	0.379
Posterior thalamic radiation R	-1.568(-2.812 - -0.325) x10^-5	0.025	-0.687(-3.143 - 1.768) x10^-5	0.876	0.667(-1.494 - 2.829) x10^-5	0.672
Posterior thalamic radiation L	-2.389(-3.669 - -1.11) x10^-5	<0.001	-1.93(-4.458 - 0.598) x10^-5	0.763	1.648(-0.577 - 3.874) x10^-5	0.379
Sagittal stratum R	-0.915(-2.106 - 0.275) x10^-5	0.191	0.258(-2.093 - 2.61) x10^-5	0.883	1.007(-1.063 - 3.078) x10^-5	0.590
Sagittal stratum L	-1.526(-2.822 - -0.23) x10^-5	0.034	-2.074(-4.634 - 0.487) x10^-5	0.763	0.125(-2.129 - 2.379) x10^-5	0.974
External capsule R	-1.73(-2.411 - -1.049) x10^-5	<0.001	0.384(-0.961 - 1.73) x10^-5	0.998	-0.587(-1.771 - 0.598) x10^-5	0.590
External capsule L	-1.857(-2.519 - -1.195) x10^-5	<0.001	-0.091(-1.399 - 1.216) x10^-5	0.998	0.87(-0.281 - 2.021) x10^-5	0.379
Cingulum cingulate gyrus R	-0.486(-1.991 - 1.02) x10^-5	0.560	0.109(-2.865 - 3.083) x10^-5	0.935	0.77(-1.848 - 3.388) x10^-5	0.674
Cingulum cingulate gyrus L	-0.352(-1.727 - 1.024) x10^-5	0.641	0.127(-2.591 - 2.844) x10^-5	0.998	-0.086(-2.478 - 2.307) x10^-5	0.985
Cingulum hippocampus R	-0.835(-2.255 - 0.585) x10^-5	0.320	0.65(-2.154 - 3.455) x10^-5	0.876	1.771(-0.698 - 4.241) x10^-5	0.379
Cingulum hippocampus L	-1.755(-3.038 - -0.472) x10^-5	0.015	0.771(-1.763 - 3.305) x10^-5	0.876	-0.304(-2.535 - 1.927) x10^-5	0.880
Fornix Stria terminalis R	-1.993(-3.55 - -0.436) x10^-5	0.022	-2.598(-5.673 - 0.478) x10^-5	0.763	1.462(-1.245 - 4.17) x10^-5	0.590
Fornix Stria terminalis. L	-2.944(-4.474 - -1.413) x10^-5	<0.001	-0.813(-3.836 - 2.21) x10^-5	0.918	1.931(-0.731 - 4.592) x10^-5	0.379
Superior longitudinal fasciculus R	-2.508(-3.42 - -1.596) x10^-5	<0.001	-0.907(-2.709 - 0.895) x10^-5	0.823	0.485(-1.101 - 2.072) x10^-5	0.672
Superior longitudinal fasciculus L	-2.394(-3.342 - -1.446) x10^-5	<0.001	0.05(-1.823 - 1.923) x10^-5	0.998	0.761(-0.888 - 2.41) x10^-5	0.590
Superior fronto.occipital fasciculus R	-2.877(-4.541 - -1.213) x10^-5	<0.001	1.922(-1.365 - 5.209) x10^-5	0.876	-1.408(-4.302 - 1.486) x10^-5	0.590
Superior fronto.occipital fasciculus L	-2.718(-4.105 - -1.332) x10^-5	<0.001	1.724(-1.014 - 4.463) x10^-5	0.876	-2.323(-4.734 - 0.088) x10^-5	0.283
Uncinate fasciculus R	-2.05(-5.535 - 1.435) x10^-5	0.320	-2.816(-9.699 - 4.068) x10^-5	0.876	2.582(-3.478 - 8.643) x10^-5	0.593
Uncinate fasciculus L	-2.178(-5.113 - 0.757) x10^-5	0.205	-4.722(-10.519 - 1.075) x10^-5	0.763	5.163(0.059 - 10.267) x10^-5	0.251
Tapetum R	-7.228(-11.721 - -2.735) x10^-5	0.005	4.176(-4.699 - 13.05) x10^-5	0.876	-3.673(-11.487 - 4.14) x10^-5	0.590
Tapetum L	-2.621(-7.18 - 1.939) x10^-5	0.326	-0.258(-9.264 - 8.748) x10^-5	0.906	6.24(-1.689 - 14.17) x10^-5	0.379

**Edge Density**	**Age**		**ASD**		**sex**	
	**Coefficient (95% CI)**	**p-value**	**Coefficient (95% CI)**	**p-value**	**Coefficient (95% CI)**	**p-value**
Middle cerebellar peduncle	0.77(-1.92 - 3.46)*10^3	0.946	-3.35(-8.66 - 1.95)*10^3	0.214	0.9(-3.77 - 5.58)*10^3	0.704
Genu of corpus callosum	4.02(0.85 - 7.2)*10^3	0.186	3.4(-2.87 - 9.66)*10^3	0.286	2.04(-3.47 - 7.56)*10^3	0.465
Body of corpus callosum	1.87(-1.09 - 4.82)*10^3	0.910	4.39(-1.45 - 10.24)*10^3	0.139	-0.9(-6.05 - 4.24)*10^3	0.73
Splenium of corpus callosum	0.12(-2.81 - 3.04)*10^3	0.986	0.52(-5.26 - 6.31)*10^3	0.859	1.55(-3.55 - 6.64)*10^3	0.549
Fornix	0.57(-7.31 - 8.46)*10^3	0.986	-0.95(-16.52 - 14.61)*10^3	0.904	10.5(-3.21 - 24.2)*10^3	0.132
Inferior cerebellar peduncle R	1.48(-1.44 - 4.41)*10^3	0.910	-0.77(-6.55 - 5)*10^3	0.792	0.3(-4.78 - 5.39)*10^3	0.906
Inferior cerebellar peduncle L	1.36(-1.91 - 4.64)*10^3	0.910	-1.88(-8.3 - 4.55)*10^3	0.564	1.78(-3.87 - 7.44)*10^3	0.534
Superior cerebellar peduncle R	-0.82(-3.47 - 1.84)*10^3	0.937	-2.33(-7.56 - 2.9)*10^3	0.379	2.06(-2.57 - 6.69)*10^3	0.38
Superior cerebellar peduncle L	0.62(-2.82 - 4.06)*10^3	0.986	-1.17(-8.03 - 5.68)*10^3	0.736	3.97(-2.01 - 9.95)*10^3	0.191
Cerebral peduncle R	0.67(-5.69 - 7.02)*10^3	0.986	11.07(-1.5 - 23.63)*10^3	0.084	2.33(-8.73 - 13.39)*10^3	0.678
Cerebral peduncle L	0.57(-5.61 - 6.74)*10^3	0.986	0.09(-12.1 - 12.28)*10^3	0.988	-12.02(-22.76 - -1.29)*10^3	0.028
Anterior limb of internal capsule R	2.58(-4.12 - 9.28)*10^3	0.910	12.25(-0.99 - 25.49)*10^3	0.069	15.27(3.61 - 26.92)*10^3	0.011
Anterior limb of internal capsule L	3.85(-2.82 - 10.52)*10^3	0.910	3.72(-9.46 - 16.9)*10^3	0.578	4.92(-6.68 - 16.52)*10^3	0.403
Posterior limb of internal capsule R	-2.22(-8.52 - 4.09)*10^3	0.910	6.52(-5.93 - 18.98)*10^3	0.303	7.14(-3.83 - 18.11)*10^3	0.2
Posterior limb of internal capsule L	-2.23(-8.85 - 4.38)*10^3	0.910	5.45(-7.61 - 18.52)*10^3	0.411	-4.09(-15.6 - 7.41)*10^3	0.483
Retrolenticular part of internal capsule R	6.04(-7.04 - 19.12)*10^3	0.910	-19.11(-44.95 - 6.73)*10^3	0.146	1.56(-21.19 - 24.31)*10^3	0.892
Retrolenticular part of internal capsule L	-5.14(-17.83 - 7.55)*10^3	0.910	-40.8(-65.85 - -15.74)*10^3	0.002	0.98(-21.08 - 23.04)*10^3	0.93
Anterior corona radiata R	2.04(-4.03 - 8.11)*10^3	0.910	7.99(-3.99 - 19.98)*10^3	0.19	3.87(-6.68 - 14.42)*10^3	0.47
Anterior corona radiata L	1.69(-3.31 - 6.68)*10^3	0.910	-0.76(-10.63 - 9.1)*10^3	0.879	14.26(5.57 - 22.94)*10^3	0.001
Superior corona radiata R	-0.54(-5.71 - 4.63)*10^3	0.986	8.73(-1.48 - 18.94)*10^3	0.093	5.67(-3.32 - 14.65)*10^3	0.215
Superior corona radiata L	-2.72(-8.32 - 2.89)*10^3	0.910	3.58(-7.49 - 14.65)*10^3	0.524	4.04(-5.7 - 13.79)*10^3	0.414
Posterior corona radiata R	-3.11(-11.7 - 5.49)*10^3	0.910	9.46(-7.52 - 26.44)*10^3	0.273	9.57(-5.38 - 24.52)*10^3	0.208
Posterior corona radiata L	-6.49(-13.94 - 0.95)*10^3	0.748	1.32(-13.39 - 16.02)*10^3	0.86	2.37(-10.57 - 15.32)*10^3	0.718
Posterior thalamic radiation R	-5.75(-13.67 - 2.18)*10^3	0.828	10.65(-5 - 26.29)*10^3	0.181	19.18(5.4 - 32.95)*10^3	0.007
Posterior thalamic radiation L	-5.7(-12.75 - 1.35)*10^3	0.756	-11.76(-25.69 - 2.16)*10^3	0.097	6.04(-6.22 - 18.31)*10^3	0.332
Sagittal stratum R	-1.93(-15.75 - 11.9)*10^3	0.986	4.39(-22.92 - 31.71)*10^3	0.751	8.55(-15.5 - 32.6)*10^3	0.483
Sagittal stratum L	-5.59(-18.58 - 7.4)*10^3	0.910	-44.58(-70.24 - -18.92)*10^3	<0.001	-12.22(-34.81 - 10.37)*10^3	0.287

External capsule R	-0.74(-11.1 - 9.63)*10^3	0.986	-0.33(-20.8 - 20.15)*10^3	0.975	-9.89(-27.92 - 8.13)*10^3	0.28
External capsule L	-1.08(-10.7 - 8.54)*10^3	0.986	3.28(-15.73 - 22.29)*10^3	0.734	16.22(-0.52 - 32.95)*10^3	0.057
Cingulum cingulate gyrus R	6.44(2.24 - 10.64)*10^3	0.129	0.36(-7.94 - 8.65)*10^3	0.932	-4.46(-11.76 - 2.84)*10^3	0.229
Cingulum cingulate gyrus L	7.13(2.07 - 12.2)*10^3	0.129	5.92(-4.09 - 15.92)*10^3	0.245	-0.51(-9.32 - 8.29)*10^3	0.909
Cingulum hippocampus R	0.19(-4.73 - 5.11)*10^3	0.986	-3.25(-12.96 - 6.47)*10^3	0.51	-8.76(-17.31 - -0.21)*10^3	0.045
Cingulum hippocampus L	0.18(-3.8 - 4.17)*10^3	0.986	-5.06(-12.93 - 2.81)*10^3	0.206	-6.83(-13.76 - 0.09)*10^3	0.053
Fornix Stria terminalis R	2.19(-8.59 - 12.96)*10^3	0.986	-1.75(-23.04 - 19.54)*10^3	0.871	9.66(-9.08 - 28.41)*10^3	0.31
Fornix Stria terminalis. L	-8.23(-18.71 - 2.25)*10^3	0.756	-6.68(-27.38 - 14.03)*10^3	0.525	-2.57(-20.8 - 15.65)*10^3	0.781
Superior longitudinal fasciculus R	-1.28(-8.48 - 5.91)*10^3	0.986	-1.38(-15.6 - 12.83)*10^3	0.848	-9(-21.52 - 3.51)*10^3	0.157
Superior longitudinal fasciculus L	-7.1(-13.86 - -0.34)*10^3	0.430	-5.47(-18.83 - 7.89)*10^3	0.42	-1.8(-13.57 - 9.96)*10^3	0.762
Superior fronto.occipital fasciculus R	-0.24(-11.02 - 10.55)*10^3	0.989	18.75(-2.56 - 40.06)*10^3	0.084	25.57(6.81 - 44.33)*10^3	0.008
Superior fronto.occipital fasciculus L	4.33(-5.93 - 14.59)*10^3	0.910	-0.05(-20.31 - 20.21)*10^3	0.996	-0.8(-18.64 - 17.04)*10^3	0.929
Uncinate fasciculus R	-0.04(-15.33 - 15.25)*10^3	0.996	-4.42(-34.62 - 25.79)*10^3	0.773	14.81(-11.78 - 41.41)*10^3	0.273
Uncinate fasciculus L	-6.02(-31.98 - 19.94)*10^3	0.986	-15.83(-67.11 - 35.45)*10^3	0.543	30.89(-14.25 - 76.04)*10^3	0.178
Tapetum R	2.35(-3.71 - 8.41)*10^3	0.910	3.99(-7.98 - 15.96)*10^3	0.511	16.58(6.04 - 27.11)*10^3	0.002
Tapetum L	-1.13(-5.87 - 3.62)*10^3	0.986	-5.29(-14.67 - 4.09)*10^3	0.267	7.04(-1.22 - 15.3)*10^3	0.094

4.2 Toddler Cohort

Fractional Anisotropy	Age		ASD		Sex	
	Coefficient (95% CI)	p-value	Coefficient (95% CI)	p-value	Coefficient (95% CI)	p-value
Middle cerebellar peduncle	1.667(1.158 - 2.177) x10^-3	<0.001	-1.33(-9.926 - 7.266) x10^-3	0.283	0.406(-9.017 - 9.83) x10^-3	0.956
Pontine crossing tract	2.11(1.341 - 2.878) x10^-3	<0.001	2.269(-10.693 - 15.23) x10^-3	0.235	0.576(-13.633 - 14.786) x10^-3	0.956
Genu of corpus callosum	3.515(2.594 - 4.435) x10^-3	<0.001	4.238(-11.286 - 19.762) x10^-3	0.197	-8.59(-25.608 - 8.428) x10^-3	0.956
Body of corpus callosum	3.504(2.427 - 4.58) x10^-3	<0.001	9.263(-8.889 - 27.415) x10^-3	0.102	-1.697(-21.596 - 18.201) x10^-3	0.956
Splenium of corpus callosum	1.785(1.049 - 2.521) x10^-3	<0.001	5.478(-6.925 - 17.881) x10^-3	0.247	-10.448(-24.045 - 3.149) x10^-3	0.956
Fornix	2.037(0.927 - 3.146) x10^-3	<0.001	-0.835(-19.546 - 17.876) x10^-3	0.308	5.683(-14.829 - 26.194) x10^-3	0.956
Corticospinal tract R	1.868(1.134 - 2.601) x10^-3	<0.001	-4.771(-17.142 - 7.6) x10^-3	0.416	0.972(-12.589 - 14.533) x10^-3	0.956
Corticospinal tract L	2.014(1.231 - 2.797) x10^-3	<0.001	0.362(-12.839 - 13.564) x10^-3	0.235	8.536(-5.936 - 23.008) x10^-3	0.956
Medial lemniscus R	1.483(0.762 - 2.205) x10^-3	<0.001	0.767(-11.401 - 12.935) x10^-3	0.304	-4.37(-17.709 - 8.969) x10^-3	0.956
Medial lemniscus L	1.843(1.204 - 2.482) x10^-3	<0.001	0.889(-9.883 - 11.661) x10^-3	0.277	-4.887(-16.696 - 6.921) x10^-3	0.956
Inferior cerebellar peduncle R	1.846(1.132 - 2.559) x10^-3	<0.001	-1.302(-13.334 - 10.73) x10^-3	0.308	-6.275(-19.465 - 6.914) x10^-3	0.956
Inferior cerebellar peduncle L	1.556(0.832 - 2.279) x10^-3	<0.001	3.855(-8.345 - 16.055) x10^-3	0.277	-7.06(-20.434 - 6.314) x10^-3	0.956
Superior cerebellar peduncle R	1.48(0.667 - 2.292) x10^-3	<0.001	-0.192(-13.888 - 13.505) x10^-3	0.308	-1.533(-16.547 - 13.481) x10^-3	0.956
Superior cerebellar peduncle L	1.59(0.788 - 2.393) x10^-3	<0.001	-0.619(-14.159 - 12.92) x10^-3	0.308	-0.874(-15.716 - 13.968) x10^-3	0.956
Cerebral peduncle R	2.601(1.839 - 3.363) x10^-3	<0.001	9.662(-3.184 - 22.508) x10^-3	0.036	2.255(-11.827 - 16.337) x10^-3	0.956
Cerebral peduncle L	3.103(2.296 - 3.91) x10^-3	<0.001	2.024(-11.582 - 15.63) x10^-3	0.109	4.669(-10.246 - 19.584) x10^-3	0.956
Anterior limb of internal capsule R	2.191(1.588 - 2.793) x10^-3	<0.001	-3.454(-13.613 - 6.705) x10^-3	0.283	2.021(-9.115 - 13.158) x10^-3	0.956
Anterior limb of internal capsule L	2.491(1.937 - 3.046) x10^-3	<0.001	-2.293(-11.638 - 7.052) x10^-3	0.189	8.641(-1.604 - 18.885) x10^-3	0.956
Posterior limb of internal capsule R	1.873(1.313 - 2.432) x10^-3	<0.001	5.152(-4.283 - 14.587) x10^-3	0.102	-1.573(-11.916 - 8.77) x10^-3	0.956
Posterior limb of internal capsule L	2.096(1.479 - 2.713) x10^-3	<0.001	4.376(-6.034 - 14.786) x10^-3	0.109	-1.925(-13.337 - 9.486) x10^-3	0.956
Retrolenticular part of internal capsule R	2.113(1.386 - 2.84) x10^-3	<0.001	-0.381(-12.635 - 11.873) x10^-3	0.283	-5.42(-18.853 - 8.014) x10^-3	0.956
Retrolenticular part of internal capsule L	2.235(1.469 - 3.002) x10^-3	<0.001	-7.367(-20.29 - 5.555) x10^-3	0.717	-8.661(-22.827 - 5.504) x10^-3	0.956
Anterior corona radiata R	2.26(1.579 - 2.942) x10^-3	<0.001	-0.882(-12.372 - 10.608) x10^-3	0.284	-8.908(-21.504 - 3.688) x10^-3	0.956
Anterior corona radiata L	2.589(1.848 - 3.331) x10^-3	<0.001	-4.027(-16.53 - 8.476) x10^-3	0.308	-3.297(-17.003 - 10.409) x10^-3	0.956
Superior corona radiata R	1.908(1.277 - 2.539) x10^-3	<0.001	-1.609(-12.248 - 9.031) x10^-3	0.308	-6.308(-17.971 - 5.356) x10^-3	0.956
Superior corona radiata L	2.204(1.52 - 2.888) x10^-3	<0.001	-1.497(-13.026 - 10.032) x10^-3	0.283	-5.579(-18.218 - 7.059) x10^-3	0.956
Posterior corona radiata R	1.483(0.742 - 2.223) x10^-3	<0.001	2.64(-9.849 - 15.128) x10^-3	0.308	-10.937(-24.627 - 2.753) x10^-3	0.956
Posterior corona radiata L	1.997(1.263 - 2.731) x10^-3	<0.001	0.618(-11.763 - 12.999) x10^-3	0.247	2.11(-11.463 - 15.682) x10^-3	0.956
Posterior thalamic radiation R	2.343(1.532 - 3.154) x10^-3	<0.001	2.767(-10.906 - 16.441) x10^-3	0.283	-15.678(-30.667 - -0.688) x10^-3	0.956
Posterior thalamic radiation L	2.462(1.559 - 3.365) x10^-3	<0.001	1.846(-13.382 - 17.074) x10^-3	0.283	-14.007(-30.7 - 2.687) x10^-3	0.956
Sagittal stratum R	2.383(1.635 - 3.13) x10^-3	<0.001	-7.332(-19.934 - 5.271) x10^-3	0.416	0.852(-12.964 - 14.667) x10^-3	0.956
Sagittal stratum L	1.98(1.281 - 2.679) x10^-3	<0.001	-3.874(-15.666 - 7.918) x10^-3	0.416	-5.689(-18.616 - 7.238) x10^-3	0.956
External capsule R	2.245(1.715 - 2.775) x10^-3	<0.001	-6.729(-15.666 - 2.208) x10^-3	0.308	3.149(-6.648 - 12.946) x10^-3	0.956
External capsule L	2.047(1.504 - 2.59) x10^-3	<0.001	-4.882(-14.033 - 4.27) x10^-3	0.308	-0.485(-10.518 - 9.547) x10^-3	0.956
Cingulum cingulate gyrus R	2.581(1.744 - 3.417) x10^-3	<0.001	-5.685(-19.791 - 8.421) x10^-3	0.283	9.778(-5.686 - 25.241) x10^-3	0.956
Cingulum cingulate gyrus L	2.987(2.148 - 3.825) x10^-3	<0.001	-4.421(-18.561 - 9.719) x10^-3	0.277	8.535(-6.966 - 24.035) x10^-3	0.956
Cingulum hippocampus R	1.5(0.434 - 2.566) x10^-3	0.006	4.755(-13.221 - 22.73) x10^-3	0.284	-5(-24.705 - 14.705) x10^-3	0.956
Cingulum hippocampus L	2.238(1.182 - 3.293) x10^-3	<0.001	-1.032(-18.829 - 16.765) x10^-3	0.308	-3.001(-22.51 - 16.508) x10^-3	0.956
Fornix Stria terminalis R	1.809(1.059 - 2.56) x10^-3	<0.001	0.801(-11.853 - 13.455) x10^-3	0.283	-3.413(-17.284 - 10.459) x10^-3	0.956
Fornix Stria terminalis. L	1.998(1.262 - 2.734) x10^-3	<0.001	-2.205(-14.617 - 10.206) x10^-3	0.283	3.279(-10.327 - 16.885) x10^-3	0.956
Superior longitudinal fasciculus R	1.882(1.157 - 2.607) x10^-3	<0.001	-1.865(-14.084 - 10.355) x10^-3	0.416	-10.404(-23.799 - 2.992) x10^-3	0.956
Superior longitudinal fasciculus L	2.243(1.538 - 2.948) x10^-3	<0.001	-8.186(-20.076 - 3.703) x10^-3	0.608	-1.811(-14.844 - 11.222) x10^-3	0.956
Superior fronto.occipital fasciculus R	2.074(1.279 - 2.868) x10^-3	<0.001	1.943(-11.453 - 15.339) x10^-3	0.247	-1.278(-15.963 - 13.408) x10^-3	0.956
Superior fronto.occipital fasciculus L	2.333(1.577 - 3.089) x10^-3	<0.001	-2.621(-15.372 - 10.129) x10^-3	0.278	5.342(-8.635 - 19.32) x10^-3	0.956
Uncinate fasciculus R	3.134(2.168 - 4.101) x10^-3	<0.001	-9.872(-26.163 - 6.419) x10^-3	0.308	11.517(-6.342 - 29.376) x10^-3	0.956
Uncinate fasciculus L	3.067(2.094 - 4.041) x10^-3	<0.001	-5.253(-21.668 - 11.161) x10^-3	0.308	-2.213(-20.207 - 15.781) x10^-3	0.956
Tapetum R	1.65(0.071 - 3.229) x10^-3	0.041	8.636(-17.991 - 35.263) x10^-3	0.283	-1.703(-30.893 - 27.486) x10^-3	0.956
Tapetum L	1.794(0.107 - 3.481) x10^-3	0.037	2.41(-26.035 - 30.855) x10^-3	0.390	-0.406(-31.588 - 30.777) x10^-3	0.979

Mean Diffusivity	Age		ASD		Sex	
	Coefficient (95% CI)	p-value	Coefficient (95% CI)	p-value	Coefficient (95% CI)	p-value
Middle cerebellar peduncle	-1.011(-1.641 - -0.38) x10^-6	0.002	-2.394(-13.027 - 8.24) x10^-6	0.109	-6.554(-18.211 - 5.103) x10^-6	0.557
Pontine crossing tract	-1.011(-1.641 - -0.38) x10^-6	0.002	-2.394(-13.027 - 8.24) x10^-6	0.109	-6.554(-18.211 - 5.103) x10^-6	0.557
Genu of corpus callosum	-5.834(-7.471 - -4.197) x10^-6	<0.001	-5.498(-33.1 - 22.103) x10^-6	0.108	13.304(-16.954 - 43.561) x10^-6	0.649
Body of corpus callosum	-5.176(-6.617 - -3.735) x10^-6	<0.001	-4.059(-28.352 - 20.235) x10^-6	0.108	14.08(-12.551 - 40.712) x10^-6	0.594
Splenium of corpus callosum	-2.617(-3.647 - -1.587) x10^-6	<0.001	-4.217(-21.583 - 13.149) x10^-6	0.164	17(-2.037 - 36.037) x10^-6	0.557
Fornix	-6.076(-9.513 - -2.64) x10^-6	<0.001	32.615(-25.325 - 90.555) x10^-6	0.961	-5.356(-68.871 - 58.16) x10^-6	0.890
Corticospinal tract R	-1.455(-2.35 - -0.561) x10^-6	0.002	8.313(-6.769 - 23.395) x10^-6	0.775	-13.267(-29.8 - 3.266) x10^-6	0.557
Corticospinal tract L	-1.424(-2.278 - -0.571) x10^-6	0.001	-1.767(-16.156 - 12.623) x10^-6	0.124	-10.125(-25.9 - 5.649) x10^-6	0.557
Medial lemniscus R	-0.583(-1.188 - 0.023) x10^-6	0.059	-0.314(-10.529 - 9.901) x10^-6	0.400	-2.857(-14.056 - 8.341) x10^-6	0.819
Medial lemniscus L	-1.188(-1.902 - -0.474) x10^-6	0.001	-1.559(-13.598 - 10.481) x10^-6	0.159	-2.589(-15.787 - 10.608) x10^-6	0.859
Inferior cerebellar peduncle R	-0.704(-1.329 - -0.079) x10^-6	0.028	-4.89(-15.433 - 5.653) x10^-6	0.124	1.731(-9.826 - 13.289) x10^-6	0.890

Inferior cerebellar peduncle L	-0.694(-1.462 - 0.073) x10^-6	0.076	-6.758(-19.697 - 6.181) x10^-6	0.109	-2.924(-17.108 - 11.26) x10^-6	0.859
Superior cerebellar peduncle R	-0.858(-1.947 - 0.231) x10^-6	0.121	4.873(-13.486 - 23.233) x10^-6	0.961	2.075(-18.052 - 22.201) x10^-6	0.890
Superior cerebellar peduncle L	-0.822(-1.84 - 0.196) x10^-6	0.112	0.174(-16.991 - 17.34) x10^-6	0.902	11.161(-7.657 - 29.978) x10^-6	0.557
Cerebral peduncle R	-2.22(-2.964 - -1.475) x10^-6	<0.001	-2.619(-15.172 - 9.934) x10^-6	0.108	-8.746(-22.507 - 5.015) x10^-6	0.557
Cerebral peduncle L	-2.164(-2.862 - -1.467) x10^-6	<0.001	-1.323(-13.087 - 10.441) x10^-6	0.108	-7.684(-20.58 - 5.211) x10^-6	0.557
Anterior limb of internal capsule R	-2.747(-3.317 - -2.177) x10^-6	<0.001	1.077(-8.533 - 10.686) x10^-6	0.108	-0.861(-11.396 - 9.673) x10^-6	0.890
Anterior limb of internal capsule L	-2.456(-3.045 - -1.868) x10^-6	<0.001	0.421(-9.507 - 10.348) x10^-6	0.108	0.399(-10.484 - 11.282) x10^-6	0.942
Posterior limb of internal capsule R	-1.856(-2.377 - -1.334) x10^-6	<0.001	-3.031(-11.826 - 5.765) x10^-6	0.108	3.465(-6.177 - 13.106) x10^-6	0.717
Posterior limb of internal capsule L	-1.785(-2.323 - -1.248) x10^-6	<0.001	-0.395(-9.46 - 8.671) x10^-6	0.122	4.057(-5.881 - 13.996) x10^-6	0.672
Retrolenticular part of internal capsule R	-2.546(-3.349 - -1.743) x10^-6	<0.001	-7.272(-20.809 - 6.266) x10^-6	0.108	14.707(-0.133 - 29.547) x10^-6	0.557
Retrolenticular part of internal capsule L	-2.45(-3.266 - -1.635) x10^-6	<0.001	-2.711(-16.467 - 11.044) x10^-6	0.122	10.239(-4.84 - 25.318) x10^-6	0.557
Anterior corona radiata R	-4.218(-5.249 - -3.187) x10^-6	<0.001	-0.258(-17.639 - 17.123) x10^-6	0.108	6.623(-12.431 - 25.677) x10^-6	0.717
Anterior corona radiata L	-4.16(-5.233 - -3.087) x10^-6	<0.001	-1.892(-19.991 - 16.207) x10^-6	0.108	6.75(-13.09 - 26.59) x10^-6	0.717
Superior corona radiata R	-3.016(-3.807 - -2.225) x10^-6	<0.001	-4.576(-17.914 - 8.763) x10^-6	0.108	7.263(-7.359 - 21.885) x10^-6	0.620
Superior corona radiata L	-2.917(-3.744 - -2.091) x10^-6	<0.001	-4.138(-18.074 - 9.799) x10^-6	0.108	12.652(-2.626 - 27.929) x10^-6	0.557
Posterior corona radiata R	-3.236(-4.5 - -1.972) x10^-6	<0.001	-13.36(-34.676 - 7.956) x10^-6	0.108	19.793(-3.574 - 43.16) x10^-6	0.557
Posterior corona radiata L	-2.945(-4.075 - -1.815) x10^-6	<0.001	-5.958(-25.013 - 13.097) x10^-6	0.113	12.77(-8.119 - 33.658) x10^-6	0.557
Posterior thalamic radiation R	-2.893(-3.992 - -1.795) x10^-6	<0.001	-8.673(-27.194 - 9.849) x10^-6	0.109	21.098(0.794 - 41.401) x10^-6	0.557
Posterior thalamic radiation L	-3.678(-4.909 - -2.447) x10^-6	<0.001	-3.864(-24.618 - 16.89) x10^-6	0.117	12.817(-9.935 - 35.568) x10^-6	0.557
Sagittal stratum R	-3.272(-4.148 - -2.396) x10^-6	<0.001	-0.636(-15.409 - 14.137) x10^-6	0.152	17.009(0.814 - 33.203) x10^-6	0.557
Sagittal stratum L	-3.19(-4.142 - -2.238) x10^-6	<0.001	-4.155(-20.21 - 11.899) x10^-6	0.109	13.686(-3.913 - 31.286) x10^-6	0.557
External capsule R	-2.425(-2.958 - -1.891) x10^-6	<0.001	-4.199(-13.189 - 4.792) x10^-6	0.108	6.955(-2.901 - 16.81) x10^-6	0.557
External capsule L	-2.4(-2.958 - -1.843) x10^-6	<0.001	-0.352(-9.754 - 9.05) x10^-6	0.108	4.467(-5.84 - 14.774) x10^-6	0.649
Cingulum cingulate gyrus R	-3.609(-4.572 - -2.646) x10^-6	<0.001	-3.03(-19.268 - 13.209) x10^-6	0.108	11.887(-5.913 - 29.688) x10^-6	0.557
Cingulum cingulate gyrus L	-3.583(-4.598 - -2.568) x10^-6	<0.001	-3.054(-20.167 - 14.058) x10^-6	0.109	14.062(-4.697 - 32.821) x10^-6	0.557
Cingulum hippocampus R	-1.51(-2.319 - -0.702) x10^-6	<0.001	-8.378(-22.014 - 5.258) x10^-6	0.108	-1.942(-16.89 - 13.007) x10^-6	0.890
Cingulum hippocampus L	-1.561(-2.295 - -0.827) x10^-6	<0.001	0.201(-12.179 - 12.58) x10^-6	0.152	-4.546(-18.117 - 9.026) x10^-6	0.717
Fornix Stria terminalis R	-1.535(-2.311 - -0.76) x10^-6	<0.001	8.173(-4.903 - 21.249) x10^-6	0.961	1.735(-12.599 - 16.069) x10^-6	0.890
Fornix Stria terminalis. L	-1.372(-2.068 - -0.675) x10^-6	<0.001	2.159(-9.587 - 13.905) x10^-6	0.370	-1.815(-14.691 - 11.061) x10^-6	0.890
Superior longitudinal fasciculus R	-2.879(-3.755 - -2.003) x10^-6	<0.001	-4.53(-19.301 - 10.241) x10^-6	0.108	12.969(-3.223 - 29.162) x10^-6	0.557
Superior longitudinal fasciculus L	-2.953(-3.882 - -2.024) x10^-6	<0.001	4.007(-11.662 - 19.677) x10^-6	0.370	12.407(-4.77 - 29.584) x10^-6	0.557
Superior fronto.occipital fasciculus R	-3.238(-4.153 - -2.323) x10^-6	<0.001	-3.665(-19.102 - 11.771) x10^-6	0.108	8.246(-8.676 - 25.168) x10^-6	0.620
Superior fronto.occipital fasciculus L	-2.956(-3.859 - -2.053) x10^-6	<0.001	-1.145(-16.376 - 14.086) x10^-6	0.152	12.885(-3.811 - 29.582) x10^-6	0.557
Uncinate fasciculus R	-2.443(-3.175 - -1.711) x10^-6	<0.001	-2.776(-15.112 - 9.56) x10^-6	0.108	-3.658(-17.182 - 9.865) x10^-6	0.813
Uncinate fasciculus L	-2.73(-3.451 - -2.009) x10^-6	<0.001	-0.688(-12.848 - 11.473) x10^-6	0.108	1.273(-12.058 - 14.604) x10^-6	0.890
Tapetum R	-4.49(-7.121 - -1.858) x10^-6	0.001	-20.852(-65.223 - 23.519) x10^-6	0.108	10.01(-38.63 - 58.651) x10^-6	0.859
Tapetum L	-7.047(-10.188 - -3.905) x10^-6	<0.001	23.86(-29.115 - 76.834) x10^-6	0.465	-26.739(-84.811 - 31.334) x10^-6	0.645

**Radial Diffusivity**	**Age**		**ASD**		**Sex**	
	**Coefficient (95% CI)**	**p-value**	**Coefficient (95% CI)**	**p-value**	**Coefficient (95% CI)**	**p-value**
Middle cerebellar peduncle	-1.094(-1.502 - -0.686) x10^-6	<0.001	-0.032(-6.916 - 6.852) x10^-6	0.112	-3.184(-10.731 - 4.362) x10^-6	0.768
Pontine crossing tract	-1.428(-1.914 - -0.942) x10^-6	<0.001	1.974(-6.217 - 10.166) x10^-6	0.112	-8.651(-17.631 - 0.329) x10^-6	0.725
Genu of corpus callosum	-4.372(-5.563 - -3.181) x10^-6	<0.001	-4.873(-24.954 - 15.208) x10^-6	0.112	10.525(-11.488 - 32.539) x10^-6	0.768
Body of corpus callosum	-4.078(-5.243 - -2.913) x10^-6	<0.001	-7.035(-26.678 - 12.608) x10^-6	0.112	7.601(-13.932 - 29.135) x10^-6	0.776
Splenium of corpus callosum	-1.894(-2.648 - -1.14) x10^-6	<0.001	-5.157(-17.87 - 7.556) x10^-6	0.112	11.985(-1.951 - 25.922) x10^-6	0.725
Fornix	-4.453(-6.891 - -2.015) x10^-6	<0.001	15.962(-25.141 - 57.065) x10^-6	0.662	-5.216(-50.274 - 39.842) x10^-6	0.891
Corticospinal tract R	-1.39(-1.96 - -0.819) x10^-6	<0.001	5.626(-4 - 15.251) x10^-6	0.498	-7.235(-17.787 - 3.316) x10^-6	0.768
Corticospinal tract L	-1.433(-1.997 - -0.87) x10^-6	<0.001	-0.41(-9.908 - 9.088) x10^-6	0.112	-7.6(-18.012 - 2.812) x10^-6	0.725
Medial lemniscus R	-0.891(-1.338 - -0.444) x10^-6	<0.001	-0.109(-7.649 - 7.432) x10^-6	0.191	-0.316(-8.582 - 7.95) x10^-6	0.940
Medial lemniscus L	-1.261(-1.696 - -0.826) x10^-6	<0.001	-0.986(-8.32 - 6.349) x10^-6	0.112	0.619(-7.421 - 8.659) x10^-6	0.898
Inferior cerebellar peduncle R	-0.98(-1.434 - -0.525) x10^-6	<0.001	-1.886(-9.55 - 5.777) x10^-6	0.139	3.57(-4.831 - 11.971) x10^-6	0.768
Inferior cerebellar peduncle L	-0.887(-1.435 - -0.339) x10^-6	0.002	-4.052(-13.285 - 5.181) x10^-6	0.112	1.532(-8.59 - 11.654) x10^-6	0.891
Superior cerebellar peduncle R	-0.993(-1.784 - -0.202) x10^-6	0.014	2.291(-11.052 - 15.634) x10^-6	0.679	2.37(-12.258 - 16.997) x10^-6	0.891
Superior cerebellar peduncle L	-1.023(-1.774 - -0.272) x10^-6	0.008	-0.016(-12.677 - 12.646) x10^-6	0.532	6.396(-7.484 - 20.276) x10^-6	0.768
Cerebral peduncle R	-2.125(-2.683 - -1.567) x10^-6	<0.001	-5.639(-15.045 - 3.767) x10^-6	0.033	-5.015(-15.327 - 5.296) x10^-6	0.768
Cerebral peduncle L	-2.33(-2.888 - -1.772) x10^-6	<0.001	-1.769(-11.179 - 7.64) x10^-6	0.077	-5.109(-15.424 - 5.206) x10^-6	0.768
Anterior limb of internal capsule R	-2.118(-2.546 - -1.69) x10^-6	<0.001	1.783(-5.438 - 9.005) x10^-6	0.112	-1.044(-8.96 - 6.872) x10^-6	0.891
Anterior limb of internal capsule L	-2.131(-2.541 - -1.72) x10^-6	<0.001	1.345(-5.579 - 8.268) x10^-6	0.112	-3.208(-10.797 - 4.382) x10^-6	0.768
Posterior limb of internal capsule R	-1.581(-1.977 - -1.185) x10^-6	<0.001	-3.693(-10.372 - 2.986) x10^-6	0.077	2.285(-5.037 - 9.606) x10^-6	0.776
Posterior limb of internal capsule L	-1.653(-2.062 - -1.244) x10^-6	<0.001	-2.297(-9.201 - 4.606) x10^-6	0.112	2.628(-4.94 - 10.196) x10^-6	0.776
Retrolenticular part of internal capsule R	-2.137(-2.772 - -1.503) x10^-6	<0.001	-2.861(-13.561 - 7.838) x10^-6	0.112	8.953(-2.776 - 20.682) x10^-6	0.725
Retrolenticular part of internal capsule L	-2.175(-2.817 - -1.534) x10^-6	<0.001	2.162(-8.653 - 12.977) x10^-6	0.279	8.767(-3.088 - 20.623) x10^-6	0.725
Anterior corona radiata R	-3.117(-3.842 - -2.391) x10^-6	<0.001	0.571(-11.668 - 12.81) x10^-6	0.112	7.533(-5.884 - 20.95) x10^-6	0.768
Anterior corona radiata L	-3.265(-4.035 - -2.495) x10^-6	<0.001	1.615(-11.369 - 14.598) x10^-6	0.112	4.523(-9.71 - 18.756) x10^-6	0.776
Superior corona radiata R	-2.253(-2.804 - -1.702) x10^-6	<0.001	-0.786(-10.074 - 8.503) x10^-6	0.112	5.472(-4.711 - 15.654) x10^-6	0.768
Superior corona radiata L	-2.334(-2.901 - -1.767) x10^-6	<0.001	-0.93(-10.496 - 8.636) x10^-6	0.112	8.554(-1.932 - 19.041) x10^-6	0.725
Posterior corona radiata R	-2.248(-3.147 - -1.349) x10^-6	<0.001	-8.092(-23.253 - 7.069) x10^-6	0.112	14.52(-2.1 - 31.14) x10^-6	0.725
Posterior corona radiata L	-2.34(-3.145 - -1.536) x10^-6	<0.001	-2.684(-16.242 - 10.875) x10^-6	0.112	5.389(-9.474 - 20.253) x10^-6	0.776
Posterior thalamic radiation R	-2.486(-3.301 - -1.67) x10^-6	<0.001	-4.907(-18.664 - 8.85) x10^-6	0.113	17.036(1.955 - 32.116) x10^-6	0.725
Posterior thalamic radiation L	-2.924(-3.86 - -1.988) x10^-6	<0.001	-2.067(-17.856 - 13.721) x10^-6	0.137	12.792(-4.516 - 30.1) x10^-6	0.725
Sagittal stratum R	-2.713(-3.383 - -2.042) x10^-6	<0.001	4.034(-7.27 - 15.338) x10^-6	0.220	6.016(-6.376 - 18.408) x10^-6	0.768
Sagittal stratum L	-2.462(-3.141 - -1.783) x10^-6	<0.001	1.011(-10.444 - 12.467) x10^-6	0.168	8.235(-4.322 - 20.793) x10^-6	0.768
External capsule R	-2.135(-2.576 - -1.693) x10^-6	<0.001	-0.087(-7.536 - 7.363) x10^-6	0.112	2.757(-5.41 - 10.923) x10^-6	0.776
External capsule L	-2.041(-2.502 - -1.58) x10^-6	<0.001	1.243(-6.526 - 9.012) x10^-6	0.112	2.944(-5.573 - 11.46) x10^-6	0.776
Cingulum cingulate gyrus R	-2.877(-3.611 - -2.143) x10^-6	<0.001	0.769(-11.605 - 13.144) x10^-6	0.112	2.461(-11.104 - 16.026) x10^-6	0.891
Cingulum cingulate gyrus L	-3.009(-3.731 - -2.286) x10^-6	<0.001	0.662(-11.528 - 12.851) x10^-6	0.112	3.636(-9.726 - 16.999) x10^-6	0.809
Cingulum hippocampus R	-1.333(-1.978 - -0.688) x10^-6	<0.001	-6.806(-17.677 - 4.064) x10^-6	0.112	1.251(-10.665 - 13.168) x10^-6	0.891
Cingulum hippocampus L	-1.666(-2.306 - -1.026) x10^-6	<0.001	-0.001(-10.795 - 10.793) x10^-6	0.128	-1.408(-13.24 - 10.425) x10^-6	0.891
Fornix Stria terminalis R	-1.518(-2.101 - -0.936) x10^-6	<0.001	3.871(-5.944 - 13.685) x10^-6	0.463	1.519(-9.239 - 12.278) x10^-6	0.891
Fornix Stria terminalis. L	-1.52(-2.069 - -0.971) x10^-6	<0.001	2.072(-7.189 - 11.334) x10^-6	0.201	-2.362(-12.515 - 7.791) x10^-6	0.860
Superior longitudinal fasciculus R	-2.294(-2.968 - -1.619) x10^-6	<0.001	-0.044(-11.42 - 11.331) x10^-6	0.168	9.319(-3.152 - 21.789) x10^-6	0.725
Superior longitudinal fasciculus L	-2.436(-3.12 - -1.753) x10^-6	<0.001	5.883(-5.639 - 17.405) x10^-6	0.450	6.345(-6.286 - 18.976) x10^-6	0.768
Superior fronto.occipital fasciculus R	-2.432(-3.056 - -1.808) x10^-6	<0.001	-2.055(-12.577 - 8.466) x10^-6	0.112	4.959(-6.575 - 16.492) x10^-6	0.768
Superior fronto.occipital fasciculus L	-2.416(-3.007 - -1.825) x10^-6	<0.001	0.593(-9.367 - 10.552) x10^-6	0.112	4.494(-6.424 - 15.413) x10^-6	0.768
Uncinate fasciculus R	-2.499(-3.184 - -1.815) x10^-6	<0.001	2.243(-9.298 - 13.783) x10^-6	0.112	-6.875(-19.526 - 5.776) x10^-6	0.768
Uncinate fasciculus L	-2.7(-3.393 - -2.007) x10^-6	<0.001	1.392(-10.294 - 13.079) x10^-6	0.112	1.086(-11.726 - 13.897) x10^-6	0.898

Tapetum R	-2.914(-4.858 - -0.969) x10^-6	0.004	-16.515(-49.308 - 16.278) x10^-6	0.112	6.172(-29.777 - 42.12) x10^-6	0.891
Tapetum L	-4.217(-6.497 - -1.937) x10^-6	<0.001	10.963(-27.478 - 49.405) x10^-6	0.450	-12.744(-54.885 - 29.396) x10^-6	0.776

**Axial Diffusivity**	**Age**		**ASD**		**Sex**	
	**Coefficient (95% CI)**	**p-value**	**Coefficient (95% CI)**	**p-value**	**Coefficient (95% CI)**	**p-value**
Middle cerebellar peduncle	0.203(-0.741 - 1.147) x10^-6	0.671	-6.564(-22.485 - 9.358) x10^-6	0.542	-10.418(-27.872 - 7.035) x10^-6	0.637
Pontine crossing tract	-0.15(-1.249 - 0.949) x10^-6	0.787	14.013(-4.522 - 32.549) x10^-6	0.643	-22.667(-42.986 - -2.348) x10^-6	0.278
Genu of corpus callosum	-4.391(-6.077 - -2.705) x10^-6	<0.001	-1.684(-30.11 - 26.743) x10^-6	0.407	7.934(-23.228 - 39.096) x10^-6	0.844
Body of corpus callosum	-3.293(-4.655 - -1.931) x10^-6	<0.001	9.268(-13.702 - 32.238) x10^-6	0.849	19.35(-5.83 - 44.53) x10^-6	0.612
Splenium of corpus callosum	-2.176(-3.348 - -1.003) x10^-6	<0.001	2.455(-17.315 - 22.225) x10^-6	0.705	15.2(-6.472 - 36.872) x10^-6	0.612
Fornix	-4.861(-8.201 - -1.52) x10^-6	0.005	49.948(-6.383 - 106.279) x10^-6	0.649	-0.068(-61.819 - 61.684) x10^-6	0.998
Corticospinal tract R	-0.208(-1.61 - 1.193) x10^-6	0.769	7.853(-15.778 - 31.483) x10^-6	0.863	-18.119(-44.024 - 7.785) x10^-6	0.612
Corticospinal tract L	0.052(-1.288 - 1.392) x10^-6	0.938	-4.059(-26.651 - 18.533) x10^-6	0.705	-7.734(-32.501 - 17.032) x10^-6	0.806
Medial lemniscus R	0.923(-0.278 - 2.124) x10^-6	0.13	-0.718(-20.969 - 19.532) x10^-6	0.863	-7.754(-29.954 - 14.445) x10^-6	0.790
Medial lemniscus L	0.197(-1.181 - 1.575) x10^-6	0.777	-1.82(-25.052 - 21.413) x10^-6	0.849	-9.805(-35.273 - 15.664) x10^-6	0.790
Inferior cerebellar peduncle R	0.849(-0.293 - 1.991) x10^-6	0.143	-8.99(-28.243 - 10.264) x10^-6	0.705	-5.089(-26.196 - 16.017) x10^-6	0.844
Inferior cerebellar peduncle L	0.558(-0.613 - 1.729) x10^-6	0.346	-7.711(-27.447 - 12.026) x10^-6	0.643	-13.956(-35.592 - 7.679) x10^-6	0.612
Superior cerebellar peduncle R	0.42(-1.163 - 2.002) x10^-6	0.6	7.037(-19.64 - 33.715) x10^-6	0.684	-0.108(-29.352 - 29.137) x10^-6	0.998
Superior cerebellar peduncle L	0.594(-0.878 - 2.065) x10^-6	0.425	1.246(-23.565 - 26.057) x10^-6	0.701	13.848(-13.35 - 41.047) x10^-6	0.709
Cerebral peduncle R	-0.286(-1.596 - 1.024) x10^-6	0.665	9.507(-12.578 - 31.592) x10^-6	0.709	-10.964(-35.174 - 13.246) x10^-6	0.742
Cerebral peduncle L	0.484(-0.767 - 1.735) x10^-6	0.444	1.507(-19.589 - 22.603) x10^-6	0.863	-7.988(-31.115 - 15.138) x10^-6	0.790
Anterior limb of internal capsule R	-1.863(-2.769 - -0.958) x10^-6	<0.001	-2.267(-17.536 - 13.002) x10^-6	0.407	0.611(-16.127 - 17.349) x10^-6	0.998
Anterior limb of internal capsule L	-1.002(-1.943 - -0.062) x10^-6	0.037	-2.243(-18.103 - 13.616) x10^-6	0.703	10.644(-6.741 - 28.03) x10^-6	0.637
Posterior limb of internal capsule R	-0.819(-1.759 - 0.121) x10^-6	0.087	2.165(-13.681 - 18.012) x10^-6	0.863	3.648(-13.723 - 21.02) x10^-6	0.859
Posterior limb of internal capsule L	-0.388(-1.451 - 0.675) x10^-6	0.471	5.82(-12.104 - 23.743) x10^-6	0.705	4.013(-15.635 - 23.662) x10^-6	0.859
Retrolenticular part of internal capsule R	-1.22(-2.298 - -0.142) x10^-6	0.027	-12.875(-31.054 - 5.304) x10^-6	0.353	16.898(-3.031 - 36.826) x10^-6	0.512
Retrolenticular part of internal capsule L	-0.85(-2.085 - 0.385) x10^-6	0.175	-14.659(-35.481 - 6.163) x10^-6	0.353	4.145(-18.68 - 26.971) x10^-6	0.863
Anterior corona radiata R	-3.314(-4.611 - -2.016) x10^-6	<0.001	-2.91(-24.794 - 18.974) x10^-6	0.353	-2.628(-26.618 - 21.362) x10^-6	0.969
Anterior corona radiata L	-2.693(-4.033 - -1.352) x10^-6	<0.001	-10.592(-33.197 - 12.012) x10^-6	0.320	6.382(-18.398 - 31.162) x10^-6	0.844
Superior corona radiata R	-2.299(-3.406 - -1.192) x10^-6	<0.001	-11.386(-30.056 - 7.284) x10^-6	0.219	5(-15.466 - 25.467) x10^-6	0.844
Superior corona radiata L	-1.736(-3.012 - -0.46) x10^-6	0.008	-9.587(-31.102 - 11.928) x10^-6	0.407	12.148(-11.437 - 35.734) x10^-6	0.709
Posterior corona radiata R	-2.939(-4.279 - -1.599) x10^-6	<0.001	-16.036(-38.636 - 6.564) x10^-6	0.219	16.356(-8.418 - 41.131) x10^-6	0.612
Posterior corona radiata L	-1.837(-3.135 - -0.54) x10^-6	0.006	-10.095(-31.971 - 11.782) x10^-6	0.422	22.483(-1.499 - 46.465) x10^-6	0.453
Posterior thalamic radiation R	-1.225(-2.576 - 0.126) x10^-6	0.075	-11.168(-33.945 - 11.609) x10^-6	0.422	12.435(-12.534 - 37.404) x10^-6	0.709
Posterior thalamic radiation L	-2.29(-3.67 - -0.911) x10^-6	0.001	-5.294(-28.554 - 17.967) x10^-6	0.407	0.057(-25.442 - 25.556) x10^-6	0.998
Sagittal stratum R	-1.661(-2.994 - -0.328) x10^-6	0.015	-13.828(-36.304 - 8.648) x10^-6	0.422	33.148(8.509 - 57.787) x10^-6	0.278
Sagittal stratum L	-2.194(-3.528 - -0.86) x10^-6	0.002	-15.217(-37.711 - 7.277) x10^-6	0.320	16.46(-8.199 - 41.119) x10^-6	0.612
External capsule R	-0.859(-1.466 - -0.252) x10^-6	0.006	-11.892(-22.132 - -1.652) x10^-6	0.219	12.715(1.489 - 23.94) x10^-6	0.278
External capsule L	-1.079(-1.709 - -0.45) x10^-6	<0.001	-4.68(-15.299 - 5.938) x10^-6	0.353	4.275(-7.365 - 15.915) x10^-6	0.790
Cingulum cingulate gyrus R	-2.214(-3.477 - -0.952) x10^-6	<0.001	-11.101(-32.383 - 10.181) x10^-6	0.407	27.879(4.549 - 51.209) x10^-6	0.278
Cingulum cingulate gyrus L	-1.732(-3.192 - -0.271) x10^-6	0.021	-11.426(-36.049 - 13.196) x10^-6	0.542	31.574(4.581 - 58.566) x10^-6	0.278
Cingulum hippocampus R	-0.548(-2.176 - 1.08) x10^-6	0.506	-4.584(-32.03 - 22.862) x10^-6	0.665	-10.018(-40.105 - 20.069) x10^-6	0.790
Cingulum hippocampus L	0.325(-1.117 - 1.766) x10^-6	0.656	0.675(-23.631 - 24.981) x10^-6	0.994	-9.704(-36.349 - 16.941) x10^-6	0.790
Fornix Stria terminalis R	-0.076(-1.394 - 1.241) x10^-6	0.909	12.699(-9.518 - 34.916) x10^-6	0.446	1.409(-22.946 - 25.764) x10^-6	0.998
Fornix Stria terminalis. L	0.443(-0.737 - 1.624) x10^-6	0.458	0.211(-19.695 - 20.116) x10^-6	0.849	1.629(-20.191 - 23.45) x10^-6	0.998
Superior longitudinal fasciculus R	-1.741(-2.77 - -0.712) x10^-6	0.001	-13.389(-30.738 - 3.959) x10^-6	0.219	10.423(-8.594 - 29.441) x10^-6	0.705
Superior longitudinal fasciculus L	-1.561(-2.699 - -0.423) x10^-6	0.008	-5.736(-24.919 - 13.448) x10^-6	0.573	18.062(-2.968 - 39.092) x10^-6	0.512
Superior fronto.occipital fasciculus R	-2.432(-3.804 - -1.061) x10^-6	<0.001	-4.3(-27.422 - 18.822) x10^-6	0.422	10.076(-15.271 - 35.422) x10^-6	0.790
Superior fronto.occipital fasciculus L	-1.653(-3.046 - -0.26) x10^-6	0.021	-5.358(-28.841 - 18.126) x10^-6	0.701	25.102(-0.642 - 50.845) x10^-6	0.448
Uncinate fasciculus R	0.167(-0.962 - 1.296) x10^-6	0.77	-14.949(-33.99 - 4.093) x10^-6	0.422	9.451(-11.423 - 30.324) x10^-6	0.742
Uncinate fasciculus L	-0.084(-1.246 - 1.078) x10^-6	0.886	-6.371(-25.963 - 13.221) x10^-6	0.684	0.814(-20.663 - 22.292) x10^-6	0.998
Tapetum R	-4.737(-7.878 - -1.596) x10^-6	0.003	-12.823(-65.786 - 40.14) x10^-6	0.422	11.399(-46.661 - 69.458) x10^-6	0.859
Tapetum L	-8.489(-11.887 - -5.092) x10^-6	<0.001	38.887(-18.399 - 96.173) x10^-6	0.705	-41.93(-104.729 - 20.868) x10^-6	0.612

**Edge Density**	**Age**		**ASD**		**sex**	
	**Coefficient (95% CI)**	**p-value**	**Coefficient (96% CI)**	**p-value**	**Coefficient (95% CI)**	**p-value**
Middle cerebellar peduncle	-148.17(-353.9 - 57.56)	0.156	0.11(-3.35 - 3.58)*10^3	0.948	-0.7(-4.5 - 3.1)*10^3	0.752
Genu of corpus callosum	4200.24(360.84 - 8039.65)	0.032	-63.97(-128.71 - 0.77)*10^3	0.053	-58.54(-129.5 - 12.43)*10^3	0.196
Body of corpus callosum	3635.24(1014.27 - 6256.21)	0.007	-6.85(-51.04 - 37.35)*10^3	0.759	-38.52(-86.96 - 9.93)*10^3	0.210
Splenium of corpus callosum	908.22(-2837.16 - 4653.6)	0.631	-18.96(-82.11 - 44.19)*10^3	0.553	-19.54(-88.77 - 49.69)*10^3	0.623
Fornix	2823.36(362.47 - 5284.25)	0.025	-28.28(-69.77 - 13.22)*10^3	0.179	-31.03(-76.52 - 14.45)*10^3	0.262
Inferior cerebellar peduncle R	-18.47(-347.69 - 310.75)	0.912	-1.53(-7.13 - 4.08)*10^3	0.59	-3.1(-9.3 - 3.11)*10^3	0.403
Inferior cerebellar peduncle L	-52.58(-212.36 - 107.2)	0.515	-1.05(-3.8 - 1.7)*10^3	0.45	-1.21(-4.23 - 1.81)*10^3	0.475
Superior cerebellar peduncle R	-36.14(-134.1 - 61.83)	0.466	-0.29(-1.96 - 1.38)*10^3	0.728	-0.86(-2.7 - 0.99)*10^3	0.433
Superior cerebellar peduncle L	-18.54(-95.11 - 58.03)	0.632	0.03(-1.26 - 1.33)*10^3	0.958	-0.79(-2.23 - 0.65)*10^3	0.383
Cerebral peduncle R	-1067.04(-2801.49 - 667.4)	0.225	6.08(-23.16 - 35.33)*10^3	0.681	-20.67(-52.73 - 11.39)*10^3	0.288
Cerebral peduncle L	249.63(-1573.32 - 2072.57)	0.786	-14.81(-45.54 - 15.93)*10^3	0.341	-35.33(-69.03 - -1.64)*10^3	0.109
Anterior limb of internal capsule R	2940.79(319.96 - 5561.61)	0.028	-20.69(-64.88 - 23.51)*10^3	0.355	-66.55(-115 - -18.11)*10^3	0.082
Anterior limb of internal capsule L	4974.91(2175.17 - 7774.66)	<0.001	-39.68(-86.89 - 7.52)*10^3	0.098	-96.68(-148.43 - -44.93)*10^3	<0.001
Posterior limb of internal capsule R	2200.35(78.35 - 4322.36)	0.042	-33.45(-69.23 - 2.33)*10^3	0.067	-42.53(-81.75 - -3.3)*10^3	0.109
Posterior limb of internal capsule L	2719.52(456.66 - 4982.39)	0.019	-36.84(-74.99 - 1.32)*10^3	0.058	-65.34(-107.16 - -23.51)*10^3	0.062
Retrolenticular part of internal capsule R	2182.03(-1410.42 - 5774.47)	0.231	-59.77(-120.35 - 0.8)*10^3	0.053	-82.03(-148.43 - -15.63)*10^3	0.097
Retrolenticular part of internal capsule L	1482.94(-1948.48 - 4914.36)	0.393	-63.38(-121.24 - -5.52)*10^3	0.032	-69.66(-133.09 - -6.23)*10^3	0.109
Anterior corona radiata R	3279.85(75.79 - 6483.9)	0.045	-41.88(-95.9 - 12.15)*10^3	0.127	-52.69(-111.91 - 6.54)*10^3	0.185
Anterior corona radiata L	4714.94(1590.94 - 7838.94)	0.003	-43.34(-96.01 - 9.34)*10^3	0.106	-69.66(-127.4 - -11.91)*10^3	0.097
Superior corona radiata R	1134.12(-544.31 - 2812.56)	0.183	-13.27(-41.57 - 15.04)*10^3	0.355	-22.56(-53.58 - 8.47)*10^3	0.240
Superior corona radiata L	1467.75(-371.65 - 3307.14)	0.117	-26.37(-57.38 - 4.65)*10^3	0.095	-41.98(-75.98 - -7.98)*10^3	0.097
Posterior corona radiata R	-1245.7(-4419.65 - 1928.25)	0.438	-13.64(-67.16 - 39.88)*10^3	0.614	-24.98(-83.65 - 33.69)*10^3	0.456
Posterior corona radiata L	-845.06(-3628.52 - 1938.39)	0.548	-4.86(-51.79 - 42.07)*10^3	0.838	-26.38(-77.83 - 25.07)*10^3	0.398
Posterior thalamic radiation R	4922.25(849.54 - 8994.96)	0.018	-48.75(-117.42 - 19.93)*10^3	0.162	-51.82(-127.1 - 23.46)*10^3	0.262
Posterior thalamic radiation L	1823.75(-2085.5 - 5733)	0.357	-24.13(-90.05 - 41.78)*10^3	0.469	-38.71(-110.96 - 33.55)*10^3	0.384
Sagittal stratum R	8425.01(3272.91 - 13577.11)	0.002	-59.27(-146.14 - 27.61)*10^3	0.179	-85.41(-180.64 - 9.82)*10^3	0.185
Sagittal stratum L	5006.01(213.97 - 9798.04)	0.041	-45.82(-126.62 - 34.98)*10^3	0.263	-77.23(-165.8 - 11.35)*10^3	0.188
External capsule R	5936.66(3168.02 - 8705.3)	<0.001	-52.66(-99.35 - -5.98)*10^3	0.027	-80.79(-131.97 - -29.62)*10^3	0.062
External capsule L	4728.52(1865.3 - 7591.73)	0.001	-65.2(-113.48 - -16.93)*10^3	0.009	-75.87(-128.79 - -22.95)*10^3	0.068

Cingulum cingulate gyrus R	2316.98(325.08 - 4308.88)	0.023	-39.04(-72.63 - -5.46)*10^3	0.023	-27.99(-64.81 - 8.82)*10^3	0.230
Cingulum cingulate gyrus L	4660.08(2493.36 - 6826.8)	<0.001	-46.99(-83.52 - -10.45)*10^3	0.012	-71.73(-111.78 - -31.68)*10^3	<0.001
Cingulum hippocampus R	1382.67(-20.73 - 2786.07)	0.053	-15.12(-38.78 - 8.54)*10^3	0.208	-27.96(-53.9 - -2.02)*10^3	0.109
Cingulum hippocampus L	494.99(-705.85 - 1695.83)	0.415	-4.73(-24.98 - 15.52)*10^3	0.644	-18.43(-40.62 - 3.77)*10^3	0.196
Fornix Stria terminalis R	2718.1(-136.89 - 5573.09)	0.062	-27.51(-75.65 - 20.62)*10^3	0.259	-44.41(-97.18 - 8.37)*10^3	0.196
Fornix Stria terminalis. L	5726.27(2460.18 - 8992.37)	<0.001	-50.84(-105.91 - 4.23)*10^3	0.07	-70.26(-130.63 - -9.88)*10^3	0.098
Superior longitudinal fasciculus R	2010.39(-1176.76 - 5197.54)	0.214	-52.43(-106.17 - 1.31)*10^3	0.056	-56.56(-115.47 - 2.36)*10^3	0.154
Superior longitudinal fasciculus L	2469.49(-970.29 - 5909.27)	0.157	-85.36(-143.36 - -27.36)*10^3	0.004	-78.07(-141.65 - -14.49)*10^3	0.097
Superior fronto.occipital fasciculus R	2330.84(-626.3 - 5287.99)	0.121	-15.89(-65.75 - 33.97)*10^3	0.529	-24.39(-79.05 - 30.27)*10^3	0.443
Superior fronto.occipital fasciculus L	4753.03(2051.43 - 7454.63)	<0.001	-18.71(-64.26 - 26.84)*10^3	0.417	-57.74(-107.68 - -7.8)*10^3	0.098
Uncinate fasciculus R	4604.44(28.38 - 9180.49)	0.049	-31.26(-108.41 - 45.9)*10^3	0.423	-63.47(-148.05 - 21.12)*10^3	0.230
Uncinate fasciculus L	5210.98(230.48 - 10191.48)	0.04	-47.05(-131.03 - 36.93)*10^3	0.269	-96.65(-188.71 - -4.59)*10^3	0.109
Tapetum R	1906.11(-778.83 - 4591.04)	0.162	-34.18(-79.45 - 11.09)*10^3	0.137	4.3(-45.33 - 53.93)*10^3	0.864
Tapetum L	668.4(-2187.82 - 3524.62)	0.643	5.89(-42.27 - 54.05)*10^3	0.809	7.2(-45.6 - 59.99)*10^3	0.807

4.3 Adolescent Cohort

**Fractional Anisotropy**	**Age**		**ASD**		**Sex**	
	**Coefficient (95% CI)**	**p-value**	**Coefficient (95% CI)**	**p-value**	**Coefficient (95% CI)**	**p-value**
Middle cerebellar peduncle	7.612(2.85 - 12.374) x10^-5	0.002	4.769(-5.026 - 14.564) x10^-3	0.283	2.091(-2.549 - 6.731) x10^-2	0.633
Pontine crossing tract	7.641(0.351 - 14.931) x10^-5	0.04	-6.639(-21.634 - 8.357) x10^-3	0.235	-2.281(-9.384 - 4.822) x10^-2	0.700
Genu of corpus callosum	-7.423(-15.401 - 0.555) x10^-5	0.068	-27.937(-44.348 - -11.526) x10^-3	0.197	2.379(-5.394 - 10.153) x10^-2	0.707
Body of corpus callosum	-7.733(-17.408 - 1.942) x10^-5	0.116	-33.76(-53.661 - -13.858) x10^-3	0.102	-4.092(-13.519 - 5.335) x10^-2	0.633
Splenium of corpus callosum	-2.955(-9.798 - 3.887) x10^-5	0.393	-18.848(-32.922 - -4.773) x10^-3	0.247	1.617(-5.05 - 8.284) x10^-2	0.797
Fornix	-24.421(-39.031 - -9.811) x10^-5	0.001	-13.654(-43.706 - 16.398) x10^-3	0.308	-8.985(-23.22 - 5.251) x10^-2	0.511
Corticospinal tract R	12.246(4.174 - 20.317) x10^-5	0.003	6.578(-10.024 - 23.18) x10^-3	0.416	7.681(-0.183 - 15.545) x10^-2	0.379
Corticospinal tract L	12.606(2.556 - 22.656) x10^-5	0.015	14.456(-6.216 - 35.128) x10^-3	0.235	4.086(-5.707 - 13.878) x10^-2	0.633
Medial lemniscus R	18.072(11.091 - 25.053) x10^-5	<0.001	1.249(-13.109 - 15.608) x10^-3	0.304	7.099(0.297 - 13.901) x10^-2	0.379
Medial lemniscus L	14.034(7.428 - 20.639) x10^-5	<0.001	-0.872(-14.459 - 12.715) x10^-3	0.277	6.249(-0.187 - 12.685) x10^-2	0.379
Inferior cerebellar peduncle R	13.106(6.299 - 19.913) x10^-5	<0.001	1.388(-12.613 - 15.389) x10^-3	0.308	8.052(1.42 - 14.684) x10^-2	0.379
Inferior cerebellar peduncle L	8.805(2.612 - 14.997) x10^-5	0.006	-0.36(-13.098 - 12.377) x10^-3	0.277	2.078(-3.955 - 8.112) x10^-2	0.700
Superior cerebellar peduncle R	10.983(-0.051 - 22.017) x10^-5	0.051	-2.144(-24.84 - 20.553) x10^-3	0.308	8.537(-2.215 - 19.288) x10^-2	0.379
Superior cerebellar peduncle L	8.686(-0.97 - 18.343) x10^-5	0.077	5.008(-14.855 - 24.871) x10^-3	0.308	7.173(-2.236 - 16.582) x10^-2	0.379
Cerebral peduncle R	8.333(-0.64 - 17.307) x10^-5	0.068	-0.194(-18.652 - 18.263) x10^-3	0.036	3.807(-4.936 - 12.55) x10^-2	0.633
Cerebral peduncle L	4.522(-3.754 - 12.799) x10^-5	0.281	-6.209(-23.233 - 10.815) x10^-3	0.109	1.204(-6.86 - 9.268) x10^-2	0.820
Anterior limb of internal capsule R	4.551(-0.73 - 9.831) x10^-5	0.09	-12.311(-23.173 - -1.448) x10^-3	0.283	-0.623(-5.769 - 4.522) x10^-2	0.845
Anterior limb of internal capsule L	10.68(5.004 - 16.357) x10^-5	<0.001	-12.391(-24.067 - -0.714) x10^-3	0.189	4.548(-0.983 - 10.079) x10^-2	0.379
Posterior limb of internal capsule R	8.96(3.227 - 14.693) x10^-5	0.003	-9.914(-21.707 - 1.878) x10^-3	0.102	4.478(-1.108 - 10.064) x10^-2	0.379
Posterior limb of internal capsule L	11.984(6.284 - 17.683) x10^-5	<0.001	-11.124(-22.847 - 0.6) x10^-3	0.109	4.642(-0.912 - 10.195) x10^-2	0.379
Retrolenticular part of internal capsule R	5.807(-0.096 - 11.709) x10^-5	0.054	-7.215(-19.356 - 4.925) x10^-3	0.283	4.847(-0.904 - 10.598) x10^-2	0.379
Retrolenticular part of internal capsule L	0.941(-5.303 - 7.185) x10^-5	0.765	-2.685(-15.528 - 10.158) x10^-3	0.717	4.578(-1.505 - 10.662) x10^-2	0.379
Anterior corona radiata R	1.65(-5.079 - 8.379) x10^-5	0.627	-6.585(-20.426 - 7.255) x10^-3	0.284	2.851(-3.705 - 9.407) x10^-2	0.633
Anterior corona radiata L	1.096(-6.375 - 8.566) x10^-5	0.771	-10.767(-26.134 - 4.599) x10^-3	0.308	5.461(-1.818 - 12.74) x10^-2	0.379
Superior corona radiata R	5.299(-0.693 - 11.29) x10^-5	0.082	-4.916(-17.24 - 7.409) x10^-3	0.308	4.358(-1.48 - 10.196) x10^-2	0.379
Superior corona radiata L	5.561(0.565 - 10.557) x10^-5	0.03	-11.217(-21.493 - -0.941) x10^-3	0.283	5.388(0.52 - 10.256) x10^-2	0.379
Posterior corona radiata R	-0.22(-6.985 - 6.546) x10^-5	0.949	-14.588(-28.505 - -0.672) x10^-3	0.308	0.12(-6.472 - 6.712) x10^-2	0.971
Posterior corona radiata L	-1.476(-8.211 - 5.258) x10^-5	0.664	-12.205(-26.057 - 1.646) x10^-3	0.247	0.974(-5.588 - 7.535) x10^-2	0.820
Posterior thalamic radiation R	-5.225(-12.015 - 1.564) x10^-5	0.13	-11.309(-25.275 - 2.657) x10^-3	0.283	1.012(-5.603 - 7.628) x10^-2	0.820
Posterior thalamic radiation L	-1.958(-8.571 - 4.655) x10^-5	0.558	-10.456(-24.058 - 3.147) x10^-3	0.283	2.083(-4.36 - 8.526) x10^-2	0.700
Sagittal stratum R	6.933(-0.518 - 14.384) x10^-5	0.068	-4.204(-19.53 - 11.121) x10^-3	0.416	3.365(-3.895 - 10.624) x10^-2	0.633
Sagittal stratum L	6.4(-0.742 - 13.542) x10^-5	0.078	-2.549(-17.24 - 12.141) x10^-3	0.416	2.97(-3.989 - 9.929) x10^-2	0.633
External capsule R	3.348(-2.34 - 9.036) x10^-5	0.245	-3.493(-15.192 - 8.207) x10^-3	0.308	0.243(-5.298 - 5.785) x10^-2	0.951
External capsule L	3.998(-1.26 - 9.256) x10^-5	0.134	-3.935(-14.751 - 6.88) x10^-3	0.308	1.042(-4.081 - 6.165) x10^-2	0.820
Cingulum cingulate gyrus R	9.344(-1.389 - 20.077) x10^-5	0.087	-10.598(-32.675 - 11.479) x10^-3	0.283	3.836(-6.622 - 14.294) x10^-2	0.681
Cingulum cingulate gyrus L	9.35(-1.471 - 20.171) x10^-5	0.089	-9.565(-31.823 - 12.693) x10^-3	0.277	3.941(-6.602 - 14.485) x10^-2	0.681
Cingulum hippocampus R	14.528(2.073 - 26.982) x10^-5	0.023	25.964(0.346 - 51.583) x10^-3	0.284	10.216(-1.92 - 22.351) x10^-2	0.379
Cingulum hippocampus L	14.325(3.049 - 25.6) x10^-5	0.013	16.005(-7.189 - 39.198) x10^-3	0.308	8.942(-2.044 - 19.929) x10^-2	0.379
Fornix Stria terminalis R	2.981(-5.569 - 11.531) x10^-5	0.49	-10.285(-27.872 - 7.302) x10^-3	0.283	4.165(-4.165 - 12.496) x10^-2	0.633
Fornix Stria terminalis. L	4.858(-3.366 - 13.083) x10^-5	0.244	-4.303(-21.22 - 12.614) x10^-3	0.283	6.705(-1.308 - 14.719) x10^-2	0.379
Superior longitudinal fasciculus R	5.521(-0.28 - 11.322) x10^-5	0.062	-5.072(-17.004 - 6.861) x10^-3	0.416	3.242(-2.41 - 8.894) x10^-2	0.563
Superior longitudinal fasciculus L	4.903(-0.441 - 10.247) x10^-5	0.072	-7.765(-18.757 - 3.227) x10^-3	0.608	2.517(-2.69 - 7.724) x10^-2	0.633
Superior fronto.occipital fasciculus R	1.623(-9.226 - 12.473) x10^-5	0.767	-12.605(-34.921 - 9.712) x10^-3	0.247	-1.757(-12.328 - 8.814) x10^-2	0.820
Superior fronto.occipital fasciculus L	7.647(-3.807 - 19.101) x10^-5	0.188	-16.365(-39.925 - 7.195) x10^-3	0.278	1.874(-9.286 - 13.035) x10^-2	0.820
Uncinate fasciculus R	8.19(-1.249 - 17.629) x10^-5	0.088	-3.809(-23.224 - 15.606) x10^-3	0.308	9.8(0.603 - 18.997) x10^-2	0.379
Uncinate fasciculus L	6.896(-2.771 - 16.563) x10^-5	0.16	-15.67(-35.555 - 4.215) x10^-3	0.308	2.08(-7.339 - 11.5) x10^-2	0.815
Tapetum R	8.351(-1.407 - 18.109) x10^-5	0.093	-15.643(-35.715 - 4.429) x10^-3	0.283	6.694(-2.814 - 16.202) x10^-2	0.417
Tapetum L	9.189(-2.092 - 20.47) x10^-5	0.109	-6.543(-29.747 - 16.661) x10^-3	0.390	6.332(-4.66 - 17.323) x10^-2	0.563

**Mean Diffusivity**	**Age**		**ASD**		**Sex**	
	**Coefficient (95% CI)**	**p-value**	**Coefficient (95% CI)**	**p-value**	**Coefficient (95% CI)**	**p-value**
Middle cerebellar peduncle	-1.392(-1.982 - -0.801) x10^-7	<0.001	0.657(-11.486 - 12.801) x10^-6	0.659	-4.467(-10.22 - 1.285) x10^-5	0.550
Pontine crossing tract	-0.959(-2.168 - 0.249) x10^-7	0.118	-18.087(-42.95 - 6.777) x10^-6	0.659	2.41(-9.36 - 14.192)x10^-5	0.903
Genu of corpus callosum	-0.174(-1.001 - 0.654) x10^-7	0.678	16.381(-0.648 - 33.41) x10^-6	0.789	-0.929(-9 - 7.14)x10^-5	0.935
Body of corpus callosum	0.059(-1.062 - 1.18) x10^-7	0.917	30(6.941 - 53.059) x10^-6	0.963	7.35(-3.57 - 18.271)x10^-5	0.555
Splenium of corpus callosum	-0.064(-0.948 - 0.819) x10^-7	0.885	14.106(-4.061 - 32.272) x10^-6	0.659	-0.57(-9.18 - 8.03)x10^-5	0.935
Fornix	14.817(8.432 - 21.203) x10^-7	<0.001	67.274(-64.067 - 198.614) x10^-6	0.789	74.023(11.808 - 136.238)x10^-5	0.320
Corticospinal tract R	-1.048(-2.231 - 0.135) x10^-7	0.082	-13.252(-37.583 - 11.079) x10^-6	0.686	-4.24(-15.77 - 7.28)x10^-5	0.771
Corticospinal tract L	-1.725(-3.014 - -0.436) x10^-7	0.009	-14.915(-41.42 - 11.59) x10^-6	0.659	-4.1(-16.656 - 8.45)x10^-5	0.791
Medial lemniscus R	-0.424(-1.44 - 0.592) x10^-7	0.409	-23.098(-44.004 - -2.193) x10^-6	0.789	1.12(-8.79 - 11.019)x10^-5	0.935
Medial lemniscus L	-0.739(-1.545 - 0.067) x10^-7	0.072	-13.28(-29.854 - 3.295) x10^-6	0.659	-2.69(-10.539 - 5.16)x10^-5	0.791

Inferior cerebellar peduncle R	-1.064(-1.648 - -0.481) x10^-7	<0.001	-4.592(-16.597 - 7.413) x10^-6	0.751	-5.48(-11.171 - 0.202)x10^-5	0.472
Inferior cerebellar peduncle L	-0.984(-1.53 - -0.438) x10^-7	<0.001	-3.898(-15.133 - 7.337) x10^-6	0.659	-2.8(-8.12 - 2.53)x10^-5	0.718
Superior cerebellar peduncle R	-0.835(-2.844 - 1.175) x10^-7	0.412	-1.97(-43.299 - 39.359) x10^-6	0.781	-8.6(-28.178 - 10.976)x10^-5	0.739
Superior cerebellar peduncle L	-0.731(-2.112 - 0.651) x10^-7	0.296	-3.638(-32.054 - 24.778) x10^-6	0.659	-9.84(-23.3 - 3.62)x10^-5	0.554
Cerebral peduncle R	-1.764(-2.93 - -0.599) x10^-7	0.003	-10.227(-34.2 - 13.747) x10^-6	0.659	1.74(-9.61 - 13.099)x10^-5	0.935
Cerebral peduncle L	-1.698(-2.833 - -0.563) x10^-7	0.004	-10.654(-34 - 12.692) x10^-6	0.659	-1.75(-12.809 - 9.31)x10^-5	0.935
Anterior limb of internal capsule R	-1.167(-1.599 - -0.735) x10^-7	<0.001	3.013(-5.877 - 11.902) x10^-6	0.789	0.327(-3.88 - 4.54)x10^-5	0.935
Anterior limb of internal capsule L	-1.235(-1.707 - -0.762) x10^-7	<0.001	4.937(-4.778 - 14.651) x10^-6	0.789	2.92(-1.68 - 7.52)x10^-5	0.596
Posterior limb of internal capsule R	-1.59(-2.088 - -1.093) x10^-7	<0.001	1.327(-8.91 - 11.565) x10^-6	0.686	-3.43(-8.28 - 1.42)x10^-5	0.555
Posterior limb of internal capsule L	-1.685(-2.224 - -1.146) x10^-7	<0.001	-0.179(-11.269 - 10.912) x10^-6	0.659	-2.49(-7.74 - 2.76)x10^-5	0.728
Retrolenticular part of internal capsule R	-1.17(-1.647 - -0.692) x10^-7	<0.001	9.286(-0.541 - 19.113) x10^-6	0.789	-2.27(-6.93 - 2.38)x10^-5	0.728
Retrolenticular part of internal capsule L	-0.97(-1.441 - -0.499) x10^-7	<0.001	6.099(-3.595 - 15.793) x10^-6	0.789	-2.76(-7.35 - 1.83)x10^-5	0.629
Anterior corona radiata R	-0.972(-1.524 - -0.42) x10^-7	<0.001	11.817(0.47 - 23.165) x10^-6	0.789	-3.65(-9.02 - 1.73)x10^-5	0.555
Anterior corona radiata L	-0.93(-1.666 - -0.193) x10^-7	0.014	20.989(5.839 - 36.139) x10^-6	0.751	-2.76(-9.94 - 4.42)x10^-5	0.771
Superior corona radiata R	-1.092(-1.579 - -0.605) x10^-7	<0.001	11.203(1.188 - 21.218) x10^-6	0.781	-0.588(-5.33 - 4.16)x10^-5	0.935
Superior corona radiata L	-1(-1.521 - -0.478) x10^-7	<0.001	13.282(2.565 - 24) x10^-6	0.781	0.088(-4.99 - 5.17)x10^-5	0.972
Posterior corona radiata R	-0.623(-1.299 - 0.052) x10^-7	0.07	21.786(7.891 - 35.681) x10^-6	0.659	-1.5(-8.09 - 5.08)x10^-5	0.903
Posterior corona radiata L	-0.942(-1.536 - -0.348) x10^-7	0.002	18.842(6.62 - 31.064) x10^-6	0.659	-3.38(-9.17 - 2.41)x10^-5	0.629
Posterior thalamic radiation R	-1.09(-1.687 - -0.493) x10^-7	<0.001	20.912(8.628 - 33.195) x10^-6	0.789	-9.05(-14.868 - -3.23)x10^-5	0.144
Posterior thalamic radiation L	-0.903(-1.508 - -0.298) x10^-7	0.004	16.291(3.85 - 28.731) x10^-6	0.789	-7.89(-13.782 - -2)x10^-5	0.216
Sagittal stratum R	-1.259(-1.98 - -0.537) x10^-7	<0.001	10.832(-4.017 - 25.681) x10^-6	0.963	-2.25(-9.28 - 4.78)x10^-5	0.791
Sagittal stratum L	-1.274(-1.839 - -0.71) x10^-7	<0.001	7.963(-3.646 - 19.573) x10^-6	0.789	-4.87(-10.368 - 0.631)x10^-5	0.550
External capsule R	-0.644(-1.145 - -0.143) x10^-7	0.012	0.382(-9.924 - 10.689) x10^-6	0.963	-0.491(-5.37 - 4.39)x10^-5	0.935
External capsule L	-0.488(-0.926 - -0.05) x10^-7	0.029	0.76(-8.242 - 9.761) x10^-6	0.963	0.925(-3.34 - 5.19)x10^-5	0.903
Cingulum cingulate gyrus R	-2.034(-2.864 - -1.205) x10^-7	<0.001	9.598(-7.467 - 26.663) x10^-6	0.944	-9.04(-17.12 - -0.953)x10^-5	0.348
Cingulum cingulate gyrus L	-0.795(-1.617 - 0.028) x10^-7	0.058	13.839(-3.078 - 30.755) x10^-6	0.659	-1.58(-9.59 - 6.43)x10^-5	0.903
Cingulum hippocampus R	-1.759(-2.764 - -0.753) x10^-7	<0.001	-7.729(-28.416 - 12.957) x10^-6	0.659	-7.73(-17.534 - 2.06)x10^-5	0.550
Cingulum hippocampus L	-1.585(-2.57 - -0.6) x10^-7	0.002	-8.949(-29.211 - 11.314) x10^-6	0.659	-4.85(-14.446 - 4.75)x10^-5	0.727
Fornix Stria terminalis R	-0.522(-1.553 - 0.509) x10^-7	0.317	21.515(0.307 - 42.723) x10^-6	0.659	-3.76(-13.806 - 6.29)x10^-5	0.771
Fornix Stria terminalis. L	-0.796(-1.443 - -0.149) x10^-7	0.017	9.377(-3.938 - 22.692) x10^-6	0.781	-6.15(-12.453 - 0.161)x10^-5	0.472
Superior longitudinal fasciculus R	-1.049(-1.6 - -0.499) x10^-7	<0.001	9.38(-1.939 - 20.699) x10^-6	0.789	-4.02(-9.38 - 1.34)x10^-5	0.554
Superior longitudinal fasciculus L	-0.797(-1.385 - -0.208) x10^-7	0.009	12(-0.106 - 24.107) x10^-6	0.789	-2.62(-8.36 - 3.11)x10^-5	0.732
Superior fronto.occipital fasciculus R	-0.857(-1.497 - -0.217) x10^-7	0.009	7.253(-5.907 - 20.414) x10^-6	0.963	0.292(-5.94 - 6.53)x10^-5	0.946
Superior fronto.occipital fasciculus L	-0.927(-1.903 - 0.049) x10^-7	0.062	24.038(3.963 - 44.113) x10^-6	0.789	0.771(-8.74 - 10.281)x10^-5	0.935
Uncinate fasciculus R	-2.247(-2.966 - -1.527) x10^-7	<0.001	-0.185(-14.985 - 14.614) x10^-6	0.963	-5.8(-12.813 - 1.21)x10^-5	0.550
Uncinate fasciculus L	-1.374(-2.04 - -0.708) x10^-7	<0.001	1.919(-11.785 - 15.623) x10^-6	0.789	-5.19(-11.685 - 1.3)x10^-5	0.550
Tapetum R	-1.279(-2.244 - -0.314) x10^-7	0.01	28.317(8.464 - 48.171) x10^-6	0.789	-3.47(-12.874 - 5.94)x10^-5	0.771
Tapetum L	-1.193(-2.467 - 0.081) x10^-7	0.066	31.344(5.142 - 57.547) x10^-6	0.659	-3.64(-16.05 - 8.77)x10^-5	0.817

**Radial Diffusivity**	**Age**		**ASD**		**Sex**	
	**Coefficient (95% CI)**	**p-value**	**Coefficient (95% CI)**	**p-value**	**Coefficient (95% CI)**	**p-value**
Middle cerebellar peduncle	-0.881(-1.278 - -0.484) x10^-7	<0.001	-2.453(-10.612 - 5.707) x10^-6	0.440	-2.94(-6.81 - 0.922)x10^-5	0.134
Pontine crossing tract	-0.749(-1.538 - 0.039) x10^-7	0.062	-6.249(-22.468 - 9.971) x10^-6	0.440	1.98(-5.71 - 9.66)x10^-5	0.611
Genu of corpus callosum	0.378(-0.309 - 1.064) x10^-7	0.278	22.584(8.458 - 36.71) x10^-6	0.488	-1.62(-8.31 - 5.07)x10^-5	0.631
Body of corpus callosum	0.553(-0.39 - 1.496) x10^-7	0.247	30.929(11.531 - 50.327) x10^-6	0.440	5.23(-3.96 - 14.421)x10^-5	0.261
Splenium of corpus callosum	0.176(-0.5 - 0.853) x10^-7	0.606	16.536(2.624 - 30.447) x10^-6	0.708	-1.09(-7.68 - 5.5)x10^-5	0.742
Fornix	10.117(5.667 - 14.568) x10^-7	<0.001	47.046(-44.494 - 138.587) x10^-6	0.556	47.625(4.26 - 90.987)x10^-5	0.032
Corticospinal tract R	-0.972(-1.803 - -0.141) x10^-7	0.022	-9.289(-26.379 - 7.802) x10^-6	0.488	-4.96(-13.053 - 3.14)x10^-5	0.227
Corticospinal tract L	-1.292(-2.288 - -0.296) x10^-7	0.012	-13.466(-33.957 - 7.024) x10^-6	0.440	-3.44(-13.151 - 6.26)x10^-5	0.483
Medial lemniscus R	-1.035(-1.707 - -0.362) x10^-7	0.003	-10.554(-24.388 - 3.279) x10^-6	0.629	-2.79(-9.35 - 3.76)x10^-5	0.399
Medial lemniscus L	-0.941(-1.478 - -0.404) x10^-7	<0.001	-5.085(-16.134 - 5.963) x10^-6	0.440	-3.9(-9.13 - 1.34)x10^-5	0.143
Inferior cerebellar peduncle R	-0.982(-1.476 - -0.489) x10^-7	<0.001	-1.195(-11.349 - 8.959) x10^-6	0.516	-5.34(-10.15 - -0.53)x10^-5	0.03
Inferior cerebellar peduncle L	-0.758(-1.207 - -0.309) x10^-7	0.001	-0.273(-9.5 - 8.954) x10^-6	0.451	-1.97(-6.34 - 2.4)x10^-5	0.373
Superior cerebellar peduncle R	-0.89(-2.284 - 0.504) x10^-7	0.208	0.833(-27.844 - 29.51) x10^-6	0.708	-7.48(-21.06 - 6.11)x10^-5	0.277
Superior cerebellar peduncle L	-0.682(-1.698 - 0.334) x10^-7	0.186	-3.319(-24.213 - 17.574) x10^-6	0.440	-7.14(-17.035 - 2.76)x10^-5	0.155
Cerebral peduncle R	-0.914(-1.843 - 0.014) x10^-7	0.054	-3.014(-22.117 - 16.09) x10^-6	0.940	-0.586(-9.63 - 8.46)x10^-5	0.898
Cerebral peduncle L	-0.753(-1.583 - 0.076) x10^-7	0.074	-0.035(-17.088 - 17.018) x10^-6	0.858	-2.15(-10.226 - 5.93)x10^-5	0.599
Anterior limb of internal capsule R	-0.607(-0.929 - -0.286) x10^-7	<0.001	7.291(0.676 - 13.905) x10^-6	0.847	0.542(-2.59 - 3.68)x10^-5	0.732
Anterior limb of internal capsule L	-0.916(-1.286 - -0.546) x10^-7	<0.001	7.886(0.27 - 15.501) x10^-6	0.607	-0.315(-3.92 - 3.29)x10^-5	0.863
Posterior limb of internal capsule R	-0.953(-1.375 - -0.531) x10^-7	<0.001	5.227(-3.446 - 13.901) x10^-6	0.940	-3.19(-7.3 - 0.917)x10^-5	0.126
Posterior limb of internal capsule L	-1.138(-1.564 - -0.712) x10^-7	<0.001	5.296(-3.466 - 14.059) x10^-6	0.889	-3.15(-7.3 - 1)x10^-5	0.135
Retrolenticular part of internal capsule R	-0.777(-1.206 - -0.347) x10^-7	<0.001	7.11(-1.724 - 15.944) x10^-6	0.889	-3.7(-7.88 - 0.486)x10^-5	0.082
Retrolenticular part of internal capsule L	-0.452(-0.891 - -0.013) x10^-7	0.044	3.641(-5.394 - 12.675) x10^-6	0.889	-3.52(-7.8 - 0.756)x10^-5	0.105
Anterior corona radiata R	-0.528(-0.989 - -0.066) x10^-7	0.025	8.386(-1.103 - 17.875) x10^-6	0.516	-2.71(-7.21 - 1.78)x10^-5	0.234
Anterior corona radiata L	-0.452(-1.057 - 0.153) x10^-7	0.141	14.028(1.582 - 26.473) x10^-6	0.440	-3.29(-9.19 - 2.6)x10^-5	0.27
Superior corona radiata R	-0.747(-1.158 - -0.336) x10^-7	<0.001	7.498(-0.953 - 15.95) x10^-6	0.440	-2.2(-6.2 - 1.8)x10^-5	0.278
Superior corona radiata L	-0.697(-1.092 - -0.302) x10^-7	<0.001	11.01(2.888 - 19.132) x10^-6	0.451	-2.36(-6.21 - 1.49)x10^-5	0.226
Posterior corona radiata R	-0.341(-0.877 - 0.195) x10^-7	0.21	16.02(4.989 - 27.052) x10^-6	0.440	-1.14(-6.36 - 4.09)x10^-5	0.667
Posterior corona radiata L	-0.429(-0.918 - 0.061) x10^-7	0.086	13.25(3.174 - 23.327) x10^-6	0.440	-2.64(-7.42 - 2.13)x10^-5	0.274
Posterior thalamic radiation R	-0.193(-0.691 - 0.306) x10^-7	0.444	14.093(3.839 - 24.347) x10^-6	0.451	-4.17(-9.03 - 0.684)x10^-5	0.091
Posterior thalamic radiation L	-0.208(-0.705 - 0.288) x10^-7	0.407	12.121(1.908 - 22.333) x10^-6	0.451	-3.73(-8.57 - 1.11)x10^-5	0.129
Sagittal stratum R	-1.014(-1.595 - -0.432) x10^-7	<0.001	4.839(-7.126 - 16.805) x10^-6	0.723	-2.96(-8.63 - 2.71)x10^-5	0.302
Sagittal stratum L	-0.949(-1.472 - -0.426) x10^-7	<0.001	3.7(-7.058 - 14.458) x10^-6	0.708	-3.67(-8.76 - 1.43)x10^-5	0.157
External capsule R	-0.418(-0.837 - 0.002) x10^-7	0.051	1.727(-6.899 - 10.353) x10^-6	0.708	-0.236(-4.32 - 3.85)x10^-5	0.909
External capsule L	-0.372(-0.743 - -0.002) x10^-7	0.049	2.092(-5.53 - 9.714) x10^-6	0.708	0.053(-3.56 - 3.66)x10^-5	0.977
Cingulum cingulate gyrus R	-1.475(-2.216 - -0.734) x10^-7	<0.001	3.802(-11.445 - 19.049) x10^-6	0.708	-6.17(-13.392 - 1.05)x10^-5	0.093
Cingulum cingulate gyrus L	-0.811(-1.546 - -0.076) x10^-7	0.031	10.405(-4.718 - 25.527) x10^-6	0.843	-2.74(-9.9 - 4.43)x10^-5	0.45
Cingulum hippocampus R	-1.368(-2.216 - -0.52) x10^-7	0.002	-13.958(-31.404 - 3.488) x10^-6	0.516	-8.36(-16.626 - -0.098)x10^-5	0.047
Cingulum hippocampus L	-1.331(-2.101 - -0.561) x10^-7	<0.001	-9.641(-25.474 - 6.193) x10^-6	0.440	-7.33(-14.826 - 0.175)x10^-5	0.055
Fornix Stria terminalis R	-0.234(-1.029 - 0.562) x10^-7	0.561	14.918(-1.44 - 31.275) x10^-6	0.451	-3.52(-11.267 - 4.23)x10^-5	0.369
Fornix Stria terminalis. L	-0.557(-1.166 - 0.051) x10^-7	0.072	6.25(-6.268 - 18.767) x10^-6	0.708	-6.08(-12.01 - -0.151)x10^-5	0.045
Superior longitudinal fasciculus R	-0.795(-1.259 - -0.33) x10^-7	0.001	6.055(-3.506 - 15.615) x10^-6	0.451	-3.36(-7.89 - 1.17)x10^-5	0.144
Superior longitudinal fasciculus L	-0.652(-1.115 - -0.189) x10^-7	0.006	8.562(-0.954 - 18.078) x10^-6	0.463	-2.56(-7.07 - 1.95)x10^-5	0.263
Superior fronto.occipital fasciculus R	-0.427(-1.001 - 0.146) x10^-7	0.142	8.805(-2.985 - 20.595) x10^-6	0.708	1.04(-4.54 - 6.63)x10^-5	0.711
Superior fronto.occipital fasciculus L	-0.642(-1.465 - 0.182) x10^-7	0.125	18.012(1.069 - 34.954) x10^-6	0.940	-0.006(-8.03 - 8.02)x10^-5	0.999
Uncinate fasciculus R	-1.424(-2.051 - -0.798) x10^-7	<0.001	1.475(-11.42 - 14.369) x10^-6	0.440	-6.15(-12.253 - -0.037)x10^-5	0.049

Uncinate fasciculus L	-0.898(-1.493 - -0.303) x10^-7	0.004	7.342(-4.899 - 19.583) x10^-6	0.556	-3.42(-9.22 - 2.38)x10^-5	0.245
Tapetum R	-0.952(-1.73 - -0.174) x10^-7	0.017	18.764(2.759 - 34.769) x10^-6	0.451	-4.68(-12.261 - 2.9)x10^-5	0.223
Tapetum L	-1.051(-2.032 - -0.07) x10^-7	0.036	16.017(-4.156 - 36.189) x10^-6	0.440	-4.63(-14.187 - 4.92)x10^-5	0.338

**Axial Diffusivity**	**Age**		**ASD**		**Sex**	
	**Coefficient (95% CI)**	**p-value**	**Coefficient (95% CI)**	**p-value**	**Coefficient (95% CI)**	**p-value**
Middle cerebellar peduncle	-1.551(-3.904 - 0.801) x10^-7	0.194	24.976(-23.408 - 73.361) x10^-6	0.367	-3.28(-26.201 - 19.638)x10^-5	0.980
Pontine crossing tract	-0.61(-2.458 - 1.237) x10^-7	0.513	-29.339(-67.34 - 8.663) x10^-6	0.319	1.97(-16.033 - 19.969)x10^-5	0.980
Genu of corpus callosum	-1.664(-2.993 - -0.334) x10^-7	0.015	-23.959(-51.302 - 3.385) x10^-6	0.195	1.75(-11.207 - 14.698)x10^-5	0.980
Body of corpus callosum	-1.472(-3.238 - 0.294) x10^-7	0.101	6.592(-29.738 - 42.921) x10^-6	0.107	7.51(-9.7 - 24.722)x10^-5	0.980
Splenium of corpus callosum	-0.714(-2.891 - 1.464) x10^-7	0.517	5.775(-39.01 - 50.561) x10^-6	0.195	2.99(-18.229 - 24.2)x10^-5	0.980
Fornix	14.092(8.001 - 20.183) x10^-7	<0.001	62.598(-62.694 - 187.891) x10^-6	0.726	79.655(20.305 - 139.005)x10^-5	0.216
Corticospinal tract R	-0.202(-3.184 - 2.78) x10^-7	0.893	6.705(-54.636 - 68.045) x10^-6	0.602	3.88(-25.174 - 32.939)x10^-5	0.980
Corticospinal tract L	-1.305(-3.796 - 1.186) x10^-7	0.301	10.389(-40.854 - 61.631) x10^-6	0.365	-0.572(-24.845 - 23.702)x10^-5	0.980
Medial lemniscus R	1.828(0.405 - 3.252) x10^-7	0.012	-34.492(-63.779 - -5.205) x10^-6	0.978	12.097(-1.78 - 25.97)x10^-5	0.696
Medial lemniscus L	0.616(-0.739 - 1.97) x10^-7	0.369	-24.029(-51.893 - 3.834) x10^-6	0.697	3.72(-9.48 - 16.917)x10^-5	0.980
Inferior cerebellar peduncle R	-0.243(-2.093 - 1.606) x10^-7	0.794	0.664(-37.386 - 38.713) x10^-6	0.578	0.652(-17.372 - 18.675)x10^-5	0.980
Inferior cerebellar peduncle L	-0.675(-2.627 - 1.277) x10^-7	0.494	0.168(-39.98 - 40.316) x10^-6	0.195	-1.16(-20.173 - 17.862)x10^-5	0.980
Superior cerebellar peduncle R	0.154(-2.338 - 2.645) x10^-7	0.903	-14.597(-65.84 - 36.646) x10^-6	0.578	-3.77(-28.042 - 20.505)x10^-5	0.980
Superior cerebellar peduncle L	-0.157(-1.944 - 1.63) x10^-7	0.862	-3.48(-40.232 - 33.272) x10^-6	0.195	-8.41(-25.815 - 9)x10^-5	0.980
Cerebral peduncle R	-2.546(-4.196 - -0.896) x10^-7	0.003	-15.372(-49.312 - 18.569) x10^-6	0.195	7.81(-8.27 - 23.886)x10^-5	0.980
Cerebral peduncle L	-2.82(-4.48 - -1.161) x10^-7	0.001	-28.099(-62.238 - 6.04) x10^-6	0.195	1.54(-14.635 - 17.708)x10^-5	0.980
Anterior limb of internal capsule R	-1.679(-2.627 - -0.731) x10^-7	<0.001	-14.481(-33.976 - 5.014) x10^-6	0.212	-0.924(-10.158 - 8.31)x10^-5	0.980
Anterior limb of internal capsule L	-0.944(-1.842 - -0.046) x10^-7	0.04	-11.529(-30.008 - 6.95) x10^-6	0.216	9.31(0.554 - 18.061)x10^-5	0.444
Posterior limb of internal capsule R	-1.913(-2.833 - -0.993) x10^-7	<0.001	-14.956(-33.88 - 3.968) x10^-6	0.195	-0.995(-9.96 - 7.97)x10^-5	0.980
Posterior limb of internal capsule L	-1.634(-2.674 - -0.594) x10^-7	0.002	-21.375(-42.774 - 0.024) x10^-6	0.212	1.35(-8.78 - 11.49)x10^-5	0.980
Retrolenticular part of internal capsule	-1.17(-2.052 - -0.288) x10^-7	0.01	5.226(-12.912 - 23.364) x10^-6	0.602	4.18(-4.41 - 12.773)x10^-5	0.980
Retrolenticular part of internal capsule	-1.543(-2.404 - -0.683) x10^-7	<0.001	6.855(-10.85 - 24.56) x10^-6	0.664	2.28(-6.11 - 10.667)x10^-5	0.980
Anterior corona radiata R	-1.34(-2.144 - -0.536) x10^-7	0.001	8.893(-7.645 - 25.432) x10^-6	0.978	-3.09(-10.923 - 4.75)x10^-5	0.980
Anterior corona radiata L	-1.433(-2.3 - -0.567) x10^-7	0.001	19.148(1.319 - 36.978) x10^-6	0.966	1.14(-7.3 - 9.59)x10^-5	0.980
Superior corona radiata R	-1.027(-1.814 - -0.239) x10^-7	0.011	9.918(-6.289 - 26.125) x10^-6	0.793	4.76(-2.91 - 12.44)x10^-5	0.980
Superior corona radiata L	-0.932(-1.708 - -0.156) x10^-7	0.019	6.375(-9.589 - 22.339) x10^-6	0.857	7.18(-0.38 - 14.745)x10^-5	0.595
Posterior corona radiata R	-0.837(-1.888 - 0.214) x10^-7	0.117	20.682(-0.93 - 42.294) x10^-6	0.602	-0.564(-10.802 - 9.67)x10^-5	0.980
Posterior corona radiata L	-1.56(-2.479 - -0.642) x10^-7	0.001	17.427(-1.465 - 36.319) x10^-6	0.960	-2.32(-11.27 - 6.63)x10^-5	0.980
Posterior thalamic radiation R	-2.715(-3.743 - -1.687) x10^-7	<0.001	19.367(-1.779 - 40.512) x10^-6	0.602	-14.835(-24.851 - -4.82)x10^-5	0.192
Posterior thalamic radiation L	-2.083(-3.119 - -1.046) x10^-7	<0.001	10.444(-10.885 - 31.773) x10^-6	0.857	-12.582(-22.686 - -2.48)x10^-5	0.240
Sagittal stratum R	-0.734(-1.954 - 0.485) x10^-7	0.235	16.061(-9.022 - 41.144) x10^-6	0.664	2(-9.88 - 13.88)x10^-5	0.980
Sagittal stratum L	-0.968(-1.911 - -0.025) x10^-7	0.044	10.571(-8.82 - 29.962) x10^-6	0.857	-3.8(-12.985 - 5.39)x10^-5	0.980
External capsule R	-0.686(-1.346 - -0.026) x10^-7	0.042	-5.515(-19.089 - 8.058) x10^-6	0.602	-0.738(-7.17 - 5.69)x10^-5	0.980
External capsule L	-0.338(-0.932 - 0.257) x10^-7	0.262	-4.205(-16.431 - 8.02) x10^-6	0.529	2.71(-3.08 - 8.5)x10^-5	0.980
Cingulum cingulate gyrus R	-1.671(-2.935 - -0.408) x10^-7	0.01	17.171(-8.828 - 43.17) x10^-6	0.462	-8.72(-21.038 - 3.59)x10^-5	0.980
Cingulum cingulate gyrus L	0.061(-1.342 - 1.464) x10^-7	0.931	13.006(-15.858 - 41.871) x10^-6	0.106	3.62(-10.051 - 17.295)x10^-5	0.980
Cingulum hippocampus R	-1.185(-2.713 - 0.342) x10^-7	0.127	15.063(-16.354 - 46.479) x10^-6	0.352	1.69(-13.193 - 16.571)x10^-5	0.980
Cingulum hippocampus L	-0.775(-2.36 - 0.81) x10^-7	0.334	0.691(-31.911 - 33.293) x10^-6	0.365	7.34(-8.1 - 22.788)x10^-5	0.980
Fornix Stria terminalis R	-0.864(-2.287 - 0.558) x10^-7	0.23	20.672(-8.583 - 49.926) x10^-6	0.529	-0.659(-14.517 - 13.198)x10^-5	0.980
Fornix Stria terminalis. L	-0.728(-1.804 - 0.348) x10^-7	0.182	7.548(-14.584 - 29.679) x10^-6	0.602	-0.133(-10.617 - 10.351)x10^-5	0.980
Superior longitudinal fasciculus R	-0.774(-1.484 - -0.063) x10^-7	0.033	9.739(-4.879 - 24.357) x10^-6	0.602	-2.15(-9.08 - 4.77)x10^-5	0.980
Superior longitudinal fasciculus L	-0.426(-1.185 - 0.333) x10^-7	0.267	9.414(-6.197 - 25.025) x10^-6	0.602	-0.372(-7.77 - 7.02)x10^-5	0.980
Superior fronto.occipital fasciculus R	-1.295(-2.444 - -0.146) x10^-7	0.028	-3.78(-27.409 - 19.85) x10^-6	0.578	-2.23(-13.428 - 8.96)x10^-5	0.980
Superior fronto.occipital fasciculus L	-0.839(-2.023 - 0.345) x10^-7	0.163	18.913(-5.442 - 43.268) x10^-6	0.529	2.26(-9.27 - 13.802)x10^-5	0.980
Uncinate fasciculus R	-2.479(-3.614 - -1.345) x10^-7	<0.001	-2.493(-25.822 - 20.835) x10^-6	0.216	1.26(-9.79 - 12.307)x10^-5	0.980
Uncinate fasciculus L	-1.46(-2.645 - -0.276) x10^-7	0.016	-17.801(-42.162 - 6.559) x10^-6	0.195	-5.46(-16.999 - 6.08)x10^-5	0.980
Tapetum R	-0.979(-4.246 - 2.287) x10^-7	0.553	47.668(-19.524 - 114.859) x10^-6	0.578	5.34(-26.489 - 37.167)x10^-5	0.980
Tapetum L	-0.423(-3.103 - 2.258) x10^-7	0.755	60.409(5.276 - 115.542) x10^-6	0.529	4.51(-21.604 - 30.628)x10^-5	0.980

**Edge Density**	**Age**		**ASD**		**Sex**	
	Coefficient (95% CI)	p-value	Coefficient (95% CI)	p-value	Coefficient (95% CI)	p-value
Middle cerebellar peduncle	-0.18(-0.43 - 0.07)x10^3	0.15	-0.41(-18.04 - 17.21)x10^3	0.963	-17.53(-35.08 - 0.03)x10^3	0.107
Genu of corpus callosum	-0.25(-0.8 - 0.31)x10^3	0.383	-13.85(-53.13 - 25.43)x10^3	0.488	-1.96(-41.08 - 37.16)x10^3	0.921
Body of corpus callosum	0.74(0.07 - 1.42)x10^3	0.032	46.26(-1.8 - 94.32)x10^3	0.059	-22.76(-70.63 - 25.11)x10^3	0.400
Splenium of corpus callosum	0.1(-0.44 - 0.64)x10^3	0.721	15.16(-23.4 - 53.72)x10^3	0.439	-53.85(-92.26 - -15.45)x10^3	0.060
Fornix	-0.55(-1.16 - 0.06)x10^3	0.077	50.97(7.83 - 94.1)x10^3	0.021	-27.69(-70.66 - 15.27)x10^3	0.265
Inferior cerebellar peduncle R	-0.38(-0.72 - -0.03)x10^3	0.032	5.52(-18.85 - 29.89)x10^3	0.656	-21.82(-46.09 - 2.46)x10^3	0.135
Inferior cerebellar peduncle L	-0.1(-0.34 - 0.15)x10^3	0.43	-7.16(-24.45 - 10.12)x10^3	0.415	-12.51(-29.72 - 4.71)x10^3	0.212
Superior cerebellar peduncle R	-0.56(-1.26 - 0.14)x10^3	0.115	-2.28(-52.04 - 47.49)x10^3	0.928	-52.18(-101.74 - -2.61)x10^3	0.104
Superior cerebellar peduncle L	-0.75(-1.58 - 0.09)x10^3	0.078	-3.44(-62.55 - 55.68)x10^3	0.909	-63.2(-122.08 - -4.32)x10^3	0.104
Cerebral peduncle R	-0.42(-0.91 - 0.08)x10^3	0.099	24.8(-10.48 - 60.07)x10^3	0.167	-34.23(-69.37 - 0.9)x10^3	0.107
Cerebral peduncle L	-0.64(-1.09 - -0.19)x10^3	0.006	-0.46(-32.38 - 31.46)x10^3	0.977	-36.66(-68.45 - -4.87)x10^3	0.096
Anterior limb of internal capsule R	0.42(-0.02 - 0.86)x10^3	0.062	5.88(-25.54 - 37.3)x10^3	0.713	-49.68(-80.98 - -18.38)x10^3	0.060
Anterior limb of internal capsule L	0.22(-0.21 - 0.65)x10^3	0.306	39.87(9.47 - 70.28)x10^3	0.01	-54.7(-84.99 - -24.42)x10^3	<0.001
Posterior limb of internal capsule R	-0.17(-0.58 - 0.24)x10^3	0.411	-1.41(-30.55 - 27.72)x10^3	0.924	-53.64(-82.66 - -24.63)x10^3	<0.001
Posterior limb of internal capsule L	-0.6(-0.95 - -0.25)x10^3	<0.001	-7.73(-32.69 - 17.23)x10^3	0.543	-43.63(-68.49 - -18.77)x10^3	<0.001
Retrolenticular part of internal capsule R	0.32(-0.32 - 0.95)x10^3	0.325	29.2(-15.83 - 74.22)x10^3	0.203	-62.37(-107.22 - -17.52)x10^3	0.060
Retrolenticular part of internal capsule L	0.31(-0.33 - 0.96)x10^3	0.34	44.35(-1.56 - 90.25)x10^3	0.058	-48.5(-94.23 - -2.78)x10^3	0.104
Anterior corona radiata R	-0.24(-0.72 - 0.23)x10^3	0.314	21.47(-12.39 - 55.32)x10^3	0.213	-14.87(-48.6 - 18.85)x10^3	0.406
Anterior corona radiata L	-0.2(-0.66 - 0.26)x10^3	0.387	35.54(2.81 - 68.28)x10^3	0.033	-9.37(-41.97 - 23.24)x10^3	0.587
Superior corona radiata R	0.35(0.02 - 0.69)x10^3	0.04	19.5(-4.49 - 43.5)x10^3	0.111	-20.92(-44.82 - 2.98)x10^3	0.138
Superior corona radiata L	0.24(-0.12 - 0.59)x10^3	0.184	22.84(-2.34 - 48.02)x10^3	0.075	-20.36(-45.44 - 4.72)x10^3	0.164
Posterior corona radiata R	0.94(0.39 - 1.49)x10^3	<0.001	47.64(8.62 - 86.67)x10^3	0.017	-27.44(-66.31 - 11.43)x10^3	0.221
Posterior corona radiata L	0.97(0.43 - 1.52)x10^3	<0.001	57.11(18.15 - 96.08)x10^3	0.004	-19.35(-58.16 - 19.46)x10^3	0.385
Posterior thalamic radiation R	0.36(-0.26 - 0.99)x10^3	0.255	44.43(0.13 - 88.73)x10^3	0.049	-67.9(-112.03 - -23.77)x10^3	0.060
Posterior thalamic radiation L	-0.01(-0.66 - 0.64)x10^3	0.972	33.52(-12.79 - 79.82)x10^3	0.155	-45.07(-91.19 - 1.05)x10^3	0.107
Sagittal stratum R	0.26(-0.54 - 1.06)x10^3	0.524	37.55(-19.17 - 94.27)x10^3	0.193	-75.2(-131.69 - -18.71)x10^3	0.060

Sagittal stratum L	0.13(-0.63 - 0.89)x10^3	0.731	35.86(-18.18 - 89.9)x10^3	0.192	-32.68(-86.51 - 21.14)x10^3	0.282
External capsule R	0.37(-0.28 - 1.02)x10^3	0.265	31.64(-14.74 - 78.03)x10^3	0.18	-54.9(-101.1 - -8.7)x10^3	0.096
External capsule L	0.29(-0.29 - 0.87)x10^3	0.322	32.22(-8.94 - 73.38)x10^3	0.124	-36.52(-77.52 - 4.48)x10^3	0.135
Cingulum cingulate gyrus R	0.76(0.2 - 1.32)x10^3	0.008	29.23(-10.47 - 68.93)x10^3	0.148	-24.85(-64.39 - 14.69)x10^3	0.271
Cingulum cingulate gyrus L	0.46(-0.14 - 1.07)x10^3	0.132	53.14(10.19 - 96.1)x10^3	0.016	-42.97(-85.75 - -0.19)x10^3	0.107
Cingulum hippocampus R	-0.2(-0.84 - 0.44)x10^3	0.542	28.44(-16.82 - 73.7)x10^3	0.217	-20.04(-65.12 - 25.04)x10^3	0.406
Cingulum hippocampus L	-0.29(-0.87 - 0.29)x10^3	0.328	-23.4(-64.84 - 18.03)x10^3	0.267	-40.4(-81.67 - 0.87)x10^3	0.107
Fornix Stria terminalis R	-0.63(-1.22 - -0.05)x10^3	0.034	28.45(-13.06 - 69.97)x10^3	0.178	-46.02(-87.37 - -4.67)x10^3	0.104
Fornix Stria terminalis. L	-0.21(-0.9 - 0.47)x10^3	0.54	32.2(-16.5 - 80.9)x10^3	0.194	-60.95(-109.46 - -12.45)x10^3	0.080
Superior longitudinal fasciculus R	0.4(-0.12 - 0.92)x10^3	0.133	41.61(4.47 - 78.74)x10^3	0.028	-39.87(-76.86 - -2.88)x10^3	0.104
Superior longitudinal fasciculus L	0.53(0.06 - 0.99)x10^3	0.026	43.46(10.54 - 76.38)x10^3	0.01	-38.43(-71.22 - -5.64)x10^3	0.096
Superior fronto.occipital fasciculus R	0.08(-0.4 - 0.56)x10^3	0.744	28.62(-5.16 - 62.4)x10^3	0.096	-30.87(-64.52 - 2.77)x10^3	0.131
Superior fronto.occipital fasciculus L	0.22(-0.26 - 0.7)x10^3	0.373	43.14(9.14 - 77.14)x10^3	0.013	-25.37(-59.24 - 8.49)x10^3	0.201
Uncinate fasciculus R	-1.57(-2.53 - -0.62)x10^3	0.001	-19.76(-87.53 - 48.01)x10^3	0.566	-56.52(-124.01 - 10.98)x10^3	0.154
Uncinate fasciculus L	-0.75(-1.43 - -0.07)x10^3	0.031	16.89(-31.42 - 65.2)x10^3	0.492	-65.2(-113.32 - -17.08)x10^3	0.060
Tapetum R	0.7(0 - 1.41)x10^3	0.05	27.29(-22.62 - 77.2)x10^3	0.282	-22.83(-72.54 - 26.88)x10^3	0.406
Tapetum L	0.05(-0.78 - 0.87)x10^3	0.91	43(-15.57 - 101.56)x10^3	0.149	-58.8(-117.13 - -0.47)x10^3	0.107

4.4 Adult Cohort

**Fractional Anisotropy**	**Age**		**ASD**		**Sex**	
	**Coefficient (95% CI)**	**p-value**	**Coefficient (95% CI)**	**p-value**	**Coefficient (95% CI)**	**p-value**
Middle cerebellar peduncle	7.612(2.85 - 12.374) x10^-5	0.002	4.769(-5.026 - 14.564) x10^-3	0.424	2.091(-2.549 - 6.731) x10^-2	0.633
Pontine crossing tract	7.641(0.351 - 14.931) x10^-5	0.04	-6.639(-21.634 - 8.357) x10^-3	0.729	-2.281(-9.384 - 4.822) x10^-2	0.700
Genu of corpus callosum	-7.423(-15.401 - 0.555) x10^-5	0.068	-27.937(-44.348 - -11.526) x10^-3	0.002	2.379(-5.394 - 10.153) x10^-2	0.707
Body of corpus callosum	-7.733(-17.408 - 1.942) x10^-5	0.116	-33.76(-53.661 - -13.858) x10^-3	0.002	-4.092(-13.519 - 5.335) x10^-2	0.633
Splenium of corpus callosum	-2.955(-9.798 - 3.887) x10^-5	0.393	-18.848(-32.922 - -4.773) x10^-3	0.024	1.617(-5.05 - 8.284) x10^-2	0.797
Fornix	-24.421(-39.031 - -9.811) x10^-5	0.001	-13.654(-43.706 - 16.398) x10^-3	0.424	-8.985(-23.22 - 5.251) x10^-2	0.511
Corticospinal tract R	12.246(4.174 - 20.317) x10^-5	0.003	6.578(-10.024 - 23.18) x10^-3	0.660	7.681(-0.183 - 15.545) x10^-2	0.379
Corticospinal tract L	12.606(2.556 - 22.656) x10^-5	0.015	14.456(-6.216 - 35.128) x10^-3	0.424	4.086(-5.707 - 13.878) x10^-2	0.633
Medial lemniscus R	18.072(11.091 - 25.053) x10^-5	<0.001	1.249(-13.109 - 15.608) x10^-3	0.729	7.099(0.297 - 13.901) x10^-2	0.379
Medial lemniscus L	14.034(7.428 - 20.639) x10^-5	<0.001	-0.872(-14.459 - 12.715) x10^-3	0.810	6.249(-0.187 - 12.685) x10^-2	0.379
Inferior cerebellar peduncle R	13.106(6.299 - 19.913) x10^-5	<0.001	1.388(-12.613 - 15.389) x10^-3	0.729	8.052(1.42 - 14.684) x10^-2	0.379
Inferior cerebellar peduncle L	8.805(2.612 - 14.997) x10^-5	0.006	-0.36(-13.098 - 12.377) x10^-3	0.866	2.078(-3.955 - 8.112) x10^-2	0.700
Superior cerebellar peduncle R	10.983(-0.051 - 22.017) x10^-5	0.051	-2.144(-24.84 - 20.553) x10^-3	0.960	8.537(-2.215 - 19.288) x10^-2	0.379
Superior cerebellar peduncle L	8.686(-0.97 - 18.343) x10^-5	0.077	5.008(-14.855 - 24.871) x10^-3	0.729	7.173(-2.236 - 16.582) x10^-2	0.379
Cerebral peduncle R	8.333(-0.64 - 17.307) x10^-5	0.068	-0.194(-18.652 - 18.263) x10^-3	0.925	3.807(-4.936 - 12.55) x10^-2	0.633
Cerebral peduncle L	4.522(-3.754 - 12.799) x10^-5	0.281	-6.209(-23.233 - 10.815) x10^-3	0.724	1.204(-6.86 - 9.268) x10^-2	0.820
Anterior limb of internal capsule R	4.551(-0.73 - 9.831) x10^-5	0.09	-12.311(-23.173 - -1.448) x10^-3	0.361	-0.623(-5.769 - 4.522) x10^-2	0.845
Anterior limb of internal capsule L	10.68(5.004 - 16.357) x10^-5	<0.001	-12.391(-24.067 - -0.714) x10^-3	0.418	4.548(-0.983 - 10.079) x10^-2	0.379
Posterior limb of internal capsule R	8.96(3.227 - 14.693) x10^-5	0.003	-9.914(-21.707 - 1.878) x10^-3	0.424	4.478(-1.108 - 10.064) x10^-2	0.379
Posterior limb of internal capsule L	11.984(6.284 - 17.683) x10^-5	<0.001	-11.124(-22.847 - 0.6) x10^-3	0.424	4.642(-0.912 - 10.195) x10^-2	0.379
Retrolenticular part of internal capsule R	5.807(-0.096 - 11.709) x10^-5	0.054	-7.215(-19.356 - 4.925) x10^-3	0.660	4.847(-0.904 - 10.598) x10^-2	0.379
Retrolenticular part of internal capsule L	0.941(-5.303 - 7.185) x10^-5	0.765	-2.685(-15.528 - 10.158) x10^-3	0.781	4.578(-1.505 - 10.662) x10^-2	0.379
Anterior corona radiata R	1.65(-5.079 - 8.379) x10^-5	0.627	-6.585(-20.426 - 7.255) x10^-3	0.681	2.851(-3.705 - 9.407) x10^-2	0.633
Anterior corona radiata L	1.096(-6.375 - 8.566) x10^-5	0.771	-10.767(-26.134 - 4.599) x10^-3	0.424	5.461(-1.818 - 12.74) x10^-2	0.379
Superior corona radiata R	5.299(-0.693 - 11.29) x10^-5	0.082	-4.916(-17.24 - 7.409) x10^-3	0.728	4.358(-1.48 - 10.196) x10^-2	0.379
Superior corona radiata L	5.561(0.565 - 10.557) x10^-5	0.03	-11.217(-21.493 - -0.941) x10^-3	0.315	5.388(0.52 - 10.256) x10^-2	0.379
Posterior corona radiata R	-0.22(-6.985 - 6.546) x10^-5	0.949	-14.588(-28.505 - -0.672) x10^-3	0.315	0.12(-6.472 - 6.712) x10^-2	0.971
Posterior corona radiata L	-1.476(-8.211 - 5.258) x10^-5	0.664	-12.205(-26.057 - 1.646) x10^-3	0.315	0.974(-5.588 - 7.535) x10^-2	0.820
Posterior thalamic radiation R	-5.225(-12.015 - 1.564) x10^-5	0.13	-11.309(-25.275 - 2.657) x10^-3	0.418	1.012(-5.603 - 7.628) x10^-2	0.820
Posterior thalamic radiation L	-1.958(-8.571 - 4.655) x10^-5	0.558	-10.456(-24.058 - 3.147) x10^-3	0.418	2.083(-4.36 - 8.526) x10^-2	0.700
Sagittal stratum R	6.933(-0.518 - 14.384) x10^-5	0.068	-4.204(-19.53 - 11.121) x10^-3	0.857	3.365(-3.895 - 10.624) x10^-2	0.633
Sagittal stratum L	6.4(-0.742 - 13.542) x10^-5	0.078	-2.549(-17.24 - 12.141) x10^-3	0.926	2.97(-3.989 - 9.929) x10^-2	0.633
External capsule R	3.348(-2.34 - 9.036) x10^-5	0.245	-3.493(-15.192 - 8.207) x10^-3	0.744	0.243(-5.298 - 5.785) x10^-2	0.951
External capsule L	3.998(-1.26 - 9.256) x10^-5	0.134	-3.935(-14.751 - 6.88) x10^-3	0.729	1.042(-4.081 - 6.165) x10^-2	0.820
Cingulum cingulate gyrus R	9.344(-1.389 - 20.077) x10^-5	0.087	-10.598(-32.675 - 11.479) x10^-3	0.721	3.836(-6.622 - 14.294) x10^-2	0.681
Cingulum cingulate gyrus L	9.35(-1.471 - 20.171) x10^-5	0.089	-9.565(-31.823 - 12.693) x10^-3	0.721	3.941(-6.602 - 14.485) x10^-2	0.681
Cingulum hippocampus R	14.528(2.073 - 26.982) x10^-5	0.023	25.964(0.346 - 51.583) x10^-3	0.315	10.216(-1.92 - 22.351) x10^-2	0.379
Cingulum hippocampus L	14.325(3.049 - 25.6) x10^-5	0.013	16.005(-7.189 - 39.198) x10^-3	0.418	8.942(-2.044 - 19.929) x10^-2	0.379
Fornix Stria terminalis R	2.981(-5.569 - 11.531) x10^-5	0.49	-10.285(-27.872 - 7.302) x10^-3	0.424	4.165(-4.165 - 12.496) x10^-2	0.633
Fornix Stria terminalis. L	4.858(-3.366 - 13.083) x10^-5	0.244	-4.303(-21.22 - 12.614) x10^-3	0.729	6.705(-1.308 - 14.719) x10^-2	0.379
Superior longitudinal fasciculus R	5.521(-0.28 - 11.322) x10^-5	0.062	-5.072(-17.004 - 6.861) x10^-3	0.721	3.242(-2.41 - 8.894) x10^-2	0.563
Superior longitudinal fasciculus L	4.903(-0.441 - 10.247) x10^-5	0.072	-7.765(-18.757 - 3.227) x10^-3	0.424	2.517(-2.69 - 7.724) x10^-2	0.633
Superior fronto.occipital fasciculus R	1.623(-9.226 - 12.473) x10^-5	0.767	-12.605(-34.921 - 9.712) x10^-3	0.660	-1.757(-12.328 - 8.814) x10^-2	0.820
Superior fronto.occipital fasciculus L	7.647(-3.807 - 19.101) x10^-5	0.188	-16.365(-39.925 - 7.195) x10^-3	0.424	1.874(-9.286 - 13.035) x10^-2	0.820
Uncinate fasciculus R	8.19(-1.249 - 17.629) x10^-5	0.088	-3.809(-23.224 - 15.606) x10^-3	0.940	9.8(0.603 - 18.997) x10^-2	0.379
Uncinate fasciculus L	6.896(-2.771 - 16.563) x10^-5	0.16	-15.67(-35.555 - 4.215) x10^-3	0.424	2.08(-7.339 - 11.5) x10^-2	0.815
Tapetum R	8.351(-1.407 - 18.109) x10^-5	0.093	-15.643(-35.715 - 4.429) x10^-3	0.424	6.694(-2.814 - 16.202) x10^-2	0.417
Tapetum L	9.189(-2.092 - 20.47) x10^-5	0.109	-6.543(-29.747 - 16.661) x10^-3	0.729	6.332(-4.66 - 17.323) x10^-2	0.563

**Mean Diffusivity**	**Age**		**ASD**		**Sex**	
	**Coefficient (95% CI)**	**p-value**	**Coefficient (95% CI)**	**p-value**	**Coefficient (95% CI)**	**p-value**
Middle cerebellar peduncle	-1.392(-1.982 - -0.801) x10^-7	<0.001	0.657(-11.486 - 12.801) x10^-6	0.863	-4.467(-10.22 - 1.285) x10^-5	0.550
Pontine crossing tract	-0.959(-2.168 - 0.249) x10^-7	0.118	-18.087(-42.95 - 6.777) x10^-6	0.294	2.414(-9.363 - 14.192) x10^-5	0.903
Genu of corpus callosum	-0.174(-1.001 - 0.654) x10^-7	0.678	16.381(-0.648 - 33.41) x10^-6	0.131	-0.929(-8.996 - 7.137) x10^-5	0.935
Body of corpus callosum	0.059(-1.062 - 1.18) x10^-7	0.917	30(6.941 - 53.059) x10^-6	0.066	7.348(-3.575 - 18.271) x10^-5	0.555
Splenium of corpus callosum	-0.064(-0.948 - 0.819) x10^-7	0.885	14.106(-4.061 - 32.272) x10^-6	0.240	-0.57(-9.176 - 8.035) x10^-5	0.935
Fornix	14.817(8.432 - 21.203) x10^-7	<0.001	67.274(-64.067 - 198.614) x10^-6	0.294	74.023(11.808 - 136.238) x10^-5	0.320
Corticospinal tract R	-1.048(-2.231 - 0.135) x10^-7	0.082	-13.252(-37.583 - 11.079) x10^-6	0.452	-4.244(-15.77 - 7.281) x10^-5	0.771

Corticospinal tract L	-1.725(-3.014 - -0.436) x10^-7	0.009	-14.915(-41.42 - 11.59) x10^-6	0.434	-4.1(-16.656 - 8.455) x10^-5	0.791
Medial lemniscus R	-0.424(-1.44 - 0.592) x10^-7	0.409	-23.098(-44.004 - -2.193) x10^-6	0.245	1.116(-8.787 - 11.019) x10^-5	0.935
Medial lemniscus L	-0.739(-1.545 - 0.067) x10^-7	0.072	-13.28(-29.854 - 3.295) x10^-6	0.294	-2.687(-10.539 - 5.164) x10^-5	0.791
Inferior cerebellar peduncle R	-1.064(-1.648 - -0.481) x10^-7	<0.001	-4.592(-16.597 - 7.413) x10^-6	0.509	-5.484(-11.171 - 0.202) x10^-5	0.472
Inferior cerebellar peduncle L	-0.984(-1.53 - -0.438) x10^-7	<0.001	-3.898(-15.133 - 7.337) x10^-6	0.498	-2.796(-8.118 - 2.526) x10^-5	0.718
Superior cerebellar peduncle R	-0.835(-2.844 - 1.175) x10^-7	0.412	-1.97(-43.299 - 39.359) x10^-6	0.897	-8.601(-28.178 - 10.976) x10^-5	0.739
Superior cerebellar peduncle L	-0.731(-2.112 - 0.651) x10^-7	0.296	-3.638(-32.054 - 24.778) x10^-6	0.897	-9.839(-23.3 - 3.621) x10^-5	0.554
Cerebral peduncle R	-1.764(-2.93 - -0.599) x10^-7	0.003	-10.227(-34.2 - 13.747) x10^-6	0.452	1.743(-9.613 - 13.099) x10^-5	0.935
Cerebral peduncle L	-1.698(-2.833 - -0.563) x10^-7	0.004	-10.654(-34 - 12.692) x10^-6	0.452	-1.75(-12.808 - 9.309) x10^-5	0.935
Anterior limb of internal capsule R	-1.167(-1.599 - -0.735) x10^-7	<0.001	3.013(-5.877 - 11.902) x10^-6	0.940	0.327(-3.884 - 4.538) x10^-5	0.935
Anterior limb of internal capsule L	-1.235(-1.707 - -0.762) x10^-7	<0.001	4.937(-4.778 - 14.651) x10^-6	0.897	2.921(-1.68 - 7.523) x10^-5	0.596
Posterior limb of internal capsule R	-1.59(-2.088 - -1.093) x10^-7	<0.001	1.327(-8.91 - 11.565) x10^-6	0.897	-3.43(-8.28 - 1.419) x10^-5	0.555
Posterior limb of internal capsule L	-1.685(-2.224 - -1.146) x10^-7	<0.001	-0.179(-11.269 - 10.912) x10^-6	0.712	-2.489(-7.743 - 2.764) x10^-5	0.728
Retrolenticular part of internal capsule R	-1.17(-1.647 - -0.692) x10^-7	<0.001	9.286(-0.541 - 19.113) x10^-6	0.393	-2.275(-6.929 - 2.38) x10^-5	0.728
Retrolenticular part of internal capsule L	-0.97(-1.441 - -0.499) x10^-7	<0.001	6.099(-3.595 - 15.793) x10^-6	0.628	-2.761(-7.352 - 1.831) x10^-5	0.629
Anterior corona radiata R	-0.972(-1.524 - -0.42) x10^-7	<0.001	11.817(0.47 - 23.165) x10^-6	0.294	-3.647(-9.022 - 1.729) x10^-5	0.555
Anterior corona radiata L	-0.93(-1.666 - -0.193) x10^-7	0.014	20.989(5.839 - 36.139) x10^-6	0.084	-2.76(-9.936 - 4.417) x10^-5	0.771
Superior corona radiata R	-1.092(-1.579 - -0.605) x10^-7	<0.001	11.203(1.188 - 21.218) x10^-6	0.294	-0.588(-5.332 - 4.156) x10^-5	0.935
Superior corona radiata L	-1(-1.521 - -0.478) x10^-7	<0.001	13.282(2.565 - 24) x10^-6	0.252	0.088(-4.989 - 5.165) x10^-5	0.972
Posterior corona radiata R	-0.623(-1.299 - 0.052) x10^-7	0.07	21.786(7.891 - 35.681) x10^-6	0.066	-1.503(-8.085 - 5.078) x10^-5	0.903
Posterior corona radiata L	-0.942(-1.536 - -0.348) x10^-7	0.002	18.842(6.62 - 31.064) x10^-6	0.067	-3.383(-9.172 - 2.406) x10^-5	0.629
Posterior thalamic radiation R	-1.09(-1.687 - -0.493) x10^-7	<0.001	20.912(8.628 - 33.195) x10^-6	0.066	-9.05(-14.868 - -3.231) x10^-5	0.144
Posterior thalamic radiation L	-0.903(-1.508 - -0.298) x10^-7	0.004	16.291(3.85 - 28.731) x10^-6	0.156	-7.889(-13.782 - -1.996) x10^-5	0.216
Sagittal stratum R	-1.259(-1.98 - -0.537) x10^-7	<0.001	10.832(-4.017 - 25.681) x10^-6	0.517	-2.25(-9.284 - 4.784) x10^-5	0.791
Sagittal stratum L	-1.274(-1.839 - -0.71) x10^-7	<0.001	7.963(-3.646 - 19.573) x10^-6	0.630	-4.868(-10.368 - 0.631) x10^-5	0.550
External capsule R	-0.644(-1.145 - -0.143) x10^-7	0.012	0.382(-9.924 - 10.689) x10^-6	0.897	-0.491(-5.373 - 4.391) x10^-5	0.935
External capsule L	-0.488(-0.926 - -0.05) x10^-7	0.029	0.76(-8.242 - 9.761) x10^-6	0.906	0.925(-3.339 - 5.189) x10^-5	0.903
Cingulum cingulate gyrus R	-2.034(-2.864 - -1.205) x10^-7	<0.001	9.598(-7.467 - 26.663) x10^-6	0.688	-9.036(-17.12 - -0.953) x10^-5	0.348
Cingulum cingulate gyrus L	-0.795(-1.617 - 0.028) x10^-7	0.058	13.839(-3.078 - 30.755) x10^-6	0.294	-1.579(-9.592 - 6.435) x10^-5	0.903
Cingulum hippocampus R	-1.759(-2.764 - -0.753) x10^-7	<0.001	-7.729(-28.416 - 12.957) x10^-6	0.509	-7.735(-17.534 - 2.064) x10^-5	0.550
Cingulum hippocampus L	-1.585(-2.57 - -0.6) x10^-7	0.002	-8.949(-29.211 - 11.314) x10^-6	0.422	-4.848(-14.446 - 4.75) x10^-5	0.727
Fornix Stria terminalis R	-0.522(-1.553 - 0.509) x10^-7	0.317	21.515(0.307 - 42.723) x10^-6	0.187	-3.76(-13.806 - 6.287) x10^-5	0.771
Fornix Stria terminalis. L	-0.796(-1.443 - -0.149) x10^-7	0.017	9.377(-3.938 - 22.692) x10^-6	0.452	-6.146(-12.453 - 0.161) x10^-5	0.472
Superior longitudinal fasciculus R	-1.049(-1.6 - -0.499) x10^-7	<0.001	9.38(-1.939 - 20.699) x10^-6	0.428	-4.018(-9.379 - 1.344) x10^-5	0.554
Superior longitudinal fasciculus L	-0.797(-1.385 - -0.208) x10^-7	0.009	12(-0.106 - 24.107) x10^-6	0.278	-2.624(-8.358 - 3.111) x10^-5	0.732
Superior fronto.occipital fasciculus R	-0.857(-1.497 - -0.217) x10^-7	0.009	7.253(-5.907 - 20.414) x10^-6	0.637	0.292(-5.942 - 6.526) x10^-5	0.946
Superior fronto.occipital fasciculus L	-0.927(-1.903 - 0.049) x10^-7	0.062	24.038(3.963 - 44.113) x10^-6	0.084	0.771(-8.738 - 10.281) x10^-5	0.935
Uncinate fasciculus R	-2.247(-2.966 - -1.527) x10^-7	<0.001	-0.185(-14.985 - 14.614) x10^-6	0.711	-5.803(-12.813 - 1.207) x10^-5	0.550
Uncinate fasciculus L	-1.374(-2.04 - -0.708) x10^-7	<0.001	1.919(-11.785 - 15.623) x10^-6	0.906	-5.194(-11.685 - 1.297) x10^-5	0.550
Tapetum R	-1.279(-2.244 - -0.314) x10^-7	0.01	28.317(8.464 - 48.171) x10^-6	0.131	-3.469(-12.874 - 5.935) x10^-5	0.771
Tapetum L	-1.193(-2.467 - 0.081) x10^-7	0.066	31.344(5.142 - 57.547) x10^-6	0.131	-3.639(-16.05 - 8.773) x10^-5	0.817

**Radial Diffusivity**	**Age**		**ASD**		**Sex**	
	**Coefficient (95% CI)**	**p-value**	**Coefficient (95% CI)**	**p-value**	**Coefficient (95% CI)**	**p-value**
Middle cerebellar peduncle	-0.881(-1.278 - -0.484) x10^-7	<0.001	-2.453(-10.612 - 5.707) x10^-6	0.537	-2.943(-6.808 - 0.922) x10^-5	0.419
Pontine crossing tract	-0.749(-1.538 - 0.039) x10^-7	0.062	-6.249(-22.468 - 9.971) x10^-6	0.522	1.976(-5.708 - 9.659) x10^-5	0.772
Genu of corpus callosum	0.378(-0.309 - 1.064) x10^-7	0.278	22.584(8.458 - 36.71) x10^-6	0.004	-1.623(-8.315 - 5.068) x10^-5	0.777
Body of corpus callosum	0.553(-0.39 - 1.496) x10^-7	0.247	30.929(11.531 - 50.327) x10^-6	0.004	5.232(-3.956 - 14.421) x10^-5	0.460
Splenium of corpus callosum	0.176(-0.5 - 0.853) x10^-7	0.606	16.536(2.624 - 30.447) x10^-6	0.044	-1.094(-7.683 - 5.496) x10^-5	0.828
Fornix	10.117(5.667 - 14.568) x10^-7	<0.001	47.046(-44.494 - 138.587) x10^-6	0.341	47.625(4.263 - 90.987) x10^-5	0.419
Corticospinal tract R	-0.972(-1.803 - -0.141) x10^-7	0.022	-9.289(-26.379 - 7.802) x10^-6	0.483	-4.957(-13.053 - 3.138) x10^-5	0.460
Corticospinal tract L	-1.292(-2.288 - -0.296) x10^-7	0.012	-13.466(-33.957 - 7.024) x10^-6	0.374	-3.445(-13.151 - 6.262) x10^-5	0.644
Medial lemniscus R	-1.035(-1.707 - -0.362) x10^-7	0.003	-10.554(-24.388 - 3.279) x10^-6	0.359	-2.794(-9.347 - 3.759) x10^-5	0.563
Medial lemniscus L	-0.941(-1.478 - -0.404) x10^-7	<0.001	-5.085(-16.134 - 5.963) x10^-6	0.424	-3.897(-9.131 - 1.337) x10^-5	0.419
Inferior cerebellar peduncle R	-0.982(-1.476 - -0.489) x10^-7	<0.001	-1.195(-11.349 - 8.959) x10^-6	0.749	-5.34(-10.15 - -0.53) x10^-5	0.419
Inferior cerebellar peduncle L	-0.758(-1.207 - -0.309) x10^-7	0.001	-0.273(-9.5 - 8.954) x10^-6	0.819	-1.969(-6.34 - 2.401) x10^-5	0.543
Superior cerebellar peduncle R	-0.89(-2.284 - 0.504) x10^-7	0.208	0.833(-27.844 - 29.51) x10^-6	0.923	-7.476(-21.06 - 6.108) x10^-5	0.460
Superior cerebellar peduncle L	-0.682(-1.698 - 0.334) x10^-7	0.186	-3.319(-24.213 - 17.574) x10^-6	0.819	-7.137(-17.035 - 2.76) x10^-5	0.419
Cerebral peduncle R	-0.914(-1.843 - 0.014) x10^-7	0.054	-3.014(-22.117 - 16.09) x10^-6	0.757	-0.586(-9.635 - 8.464) x10^-5	0.949
Cerebral peduncle L	-0.753(-1.583 - 0.076) x10^-7	0.074	-0.035(-17.088 - 17.018) x10^-6	0.923	-2.148(-10.226 - 5.93) x10^-5	0.772
Anterior limb of internal capsule R	-0.607(-0.929 - -0.286) x10^-7	<0.001	7.291(0.676 - 13.905) x10^-6	0.341	0.542(-2.591 - 3.675) x10^-5	0.828
Anterior limb of internal capsule L	-0.916(-1.286 - -0.546) x10^-7	<0.001	7.886(0.27 - 15.501) x10^-6	0.374	-0.315(-3.922 - 3.293) x10^-5	0.941
Posterior limb of internal capsule R	-0.953(-1.375 - -0.531) x10^-7	<0.001	5.227(-3.446 - 13.901) x10^-6	0.567	-3.191(-7.3 - 0.917) x10^-5	0.419
Posterior limb of internal capsule L	-1.138(-1.564 - -0.712) x10^-7	<0.001	5.296(-3.466 - 14.059) x10^-6	0.676	-3.149(-7.299 - 1.002) x10^-5	0.419
Retrolenticular part of internal capsule R	-0.777(-1.206 - -0.347) x10^-7	<0.001	7.11(-1.724 - 15.944) x10^-6	0.415	-3.699(-7.884 - 0.486) x10^-5	0.419
Retrolenticular part of internal capsule L	-0.452(-0.891 - -0.013) x10^-7	0.044	3.641(-5.394 - 12.675) x10^-6	0.722	-3.524(-7.803 - 0.756) x10^-5	0.419
Anterior corona radiata R	-0.528(-0.989 - -0.066) x10^-7	0.025	8.386(-1.103 - 17.875) x10^-6	0.341	-2.711(-7.206 - 1.783) x10^-5	0.460
Anterior corona radiata L	-0.452(-1.057 - 0.153) x10^-7	0.141	14.028(1.582 - 26.473) x10^-6	0.132	-3.292(-9.187 - 2.604) x10^-5	0.460
Superior corona radiata R	-0.747(-1.158 - -0.336) x10^-7	<0.001	7.498(-0.953 - 15.95) x10^-6	0.374	-2.2(-6.203 - 1.804) x10^-5	0.460
Superior corona radiata L	-0.697(-1.092 - -0.302) x10^-7	<0.001	11.01(2.888 - 19.132) x10^-6	0.132	-2.36(-6.207 - 1.487) x10^-5	0.460
Posterior corona radiata R	-0.341(-0.877 - 0.195) x10^-7	0.21	16.02(4.989 - 27.052) x10^-6	0.065	-1.137(-6.362 - 4.089) x10^-5	0.800
Posterior corona radiata L	-0.429(-0.918 - 0.061) x10^-7	0.086	13.25(3.174 - 23.327) x10^-6	0.066	-2.644(-7.417 - 2.13) x10^-5	0.460
Posterior thalamic radiation R	-0.193(-0.691 - 0.306) x10^-7	0.444	14.093(3.839 - 24.347) x10^-6	0.066	-4.174(-9.031 - 0.684) x10^-5	0.419
Posterior thalamic radiation L	-0.208(-0.705 - 0.288) x10^-7	0.407	12.121(1.908 - 22.333) x10^-6	0.132	-3.729(-8.566 - 1.109) x10^-5	0.419
Sagittal stratum R	-1.014(-1.595 - -0.432) x10^-7	<0.001	4.839(-7.126 - 16.805) x10^-6	0.827	-2.961(-8.629 - 2.707) x10^-5	0.483
Sagittal stratum L	-0.949(-1.472 - -0.426) x10^-7	<0.001	3.7(-7.058 - 14.458) x10^-6	0.872	-3.666(-8.762 - 1.43) x10^-5	0.419
External capsule R	-0.418(-0.837 - 0.002) x10^-7	0.051	1.727(-6.899 - 10.353) x10^-6	0.916	-0.236(-4.322 - 3.85) x10^-5	0.949
External capsule L	-0.372(-0.743 - -0.002) x10^-7	0.049	2.092(-5.53 - 9.714) x10^-6	0.857	0.053(-3.557 - 3.663) x10^-5	0.998
Cingulum cingulate gyrus R	-1.475(-2.216 - -0.734) x10^-7	<0.001	3.802(-11.445 - 19.049) x10^-6	0.942	-6.169(-13.392 - 1.054) x10^-5	0.419
Cingulum cingulate gyrus L	-0.811(-1.546 - -0.076) x10^-7	0.031	10.405(-4.718 - 25.527) x10^-6	0.374	-2.738(-9.901 - 4.425) x10^-5	0.617
Cingulum hippocampus R	-1.368(-2.216 - -0.52) x10^-7	0.002	-13.958(-31.404 - 3.488) x10^-6	0.318	-8.362(-16.626 - -0.098) x10^-5	0.419
Cingulum hippocampus L	-1.331(-2.101 - -0.561) x10^-7	<0.001	-9.641(-25.474 - 6.193) x10^-6	0.359	-7.326(-14.826 - 0.175) x10^-5	0.419
Fornix Stria terminalis R	-0.234(-1.029 - 0.562) x10^-7	0.561	14.918(-1.44 - 31.275) x10^-6	0.180	-3.518(-11.267 - 4.23) x10^-5	0.543
Fornix Stria terminalis. L	-0.557(-1.166 - 0.051) x10^-7	0.072	6.25(-6.268 - 18.767) x10^-6	0.537	-6.081(-12.01 - -0.151) x10^-5	0.419
Superior longitudinal fasciculus R	-0.795(-1.259 - -0.33) x10^-7	0.001	6.055(-3.506 - 15.615) x10^-6	0.516	-3.36(-7.889 - 1.168) x10^-5	0.419
Superior longitudinal fasciculus L	-0.652(-1.115 - -0.189) x10^-7	0.006	8.562(-0.954 - 18.078) x10^-6	0.341	-2.558(-7.066 - 1.95) x10^-5	0.460

Superior fronto.occipital fasciculus R	-0.427(-1.001 - 0.146) x10^-7	0.142	8.805(-2.985 - 20.595) x10^-6	0.424	1.044(-4.541 - 6.629) x10^-5	0.828
Superior fronto.occipital fasciculus L	-0.642(-1.465 - 0.182) x10^-7	0.125	18.012(1.069 - 34.954) x10^-6	0.132	-0.006(-8.031 - 8.019) x10^-5	0.999
Uncinate fasciculus R	-1.424(-2.051 - -0.798) x10^-7	<0.001	1.475(-11.42 - 14.369) x10^-6	0.857	-6.145(-12.253 - -0.037) x10^-5	0.419
Uncinate fasciculus L	-0.898(-1.493 - -0.303) x10^-7	0.004	7.342(-4.899 - 19.583) x10^-6	0.557	-3.419(-9.217 - 2.38) x10^-5	0.460
Tapetum R	-0.952(-1.73 - -0.174) x10^-7	0.017	18.764(2.759 - 34.769) x10^-6	0.235	-4.679(-12.26 - 2.903) x10^-5	0.460
Tapetum L	-1.051(-2.032 - -0.07) x10^-7	0.036	16.017(-4.156 - 36.189) x10^-6	0.341	-4.631(-14.187 - 4.924) x10^-5	0.523

**Axial Diffusivity**	**Age**		**ASD**		**Sex**	
	**Coefficient (95% CI)**	**p-value**	**Coefficient (95% CI)**	**p-value**	**Coefficient (95% CI)**	**p-value**
Middle cerebellar peduncle	-1.551(-3.904 - 0.801) x10^-7	0.194	24.976(-23.408 - 73.361) x10^-6	0.695	-3.281(-26.201 - 19.638) x10^-5	0.980
Pontine crossing tract	-0.61(-2.458 - 1.237) x10^-7	0.513	-29.339(-67.34 - 8.663) x10^-6	0.348	1.968(-16.033 - 19.969) x10^-5	0.980
Genu of corpus callosum	-1.664(-2.993 - -0.334) x10^-7	0.015	-23.959(-51.302 - 3.385) x10^-6	0.348	1.745(-11.207 - 14.697) x10^-5	0.980
Body of corpus callosum	-1.472(-3.238 - 0.294) x10^-7	0.101	6.592(-29.738 - 42.921) x10^-6	0.695	7.513(-9.696 - 24.722) x10^-5	0.980
Splenium of corpus callosum	-0.714(-2.891 - 1.464) x10^-7	0.517	5.775(-39.01 - 50.561) x10^-6	0.588	2.985(-18.229 - 24.2) x10^-5	0.980
Fornix	14.092(8.001 - 20.183) x10^-7	<0.001	62.598(-62.694 - 187.891) x10^-6	0.431	79.655(20.305 - 139.005) x10^-5	0.216
Corticospinal tract R	-0.202(-3.184 - 2.78) x10^-7	0.893	6.705(-54.636 - 68.045) x10^-6	0.588	3.882(-25.174 - 32.939) x10^-5	0.980
Corticospinal tract L	-1.305(-3.796 - 1.186) x10^-7	0.301	10.389(-40.854 - 61.631) x10^-6	0.695	-0.572(-24.845 - 23.702) x10^-5	0.980
Medial lemniscus R	1.828(0.405 - 3.252) x10^-7	0.012	-34.492(-63.779 - -5.205) x10^-6	0.348	12.097(-1.777 - 25.97) x10^-5	0.696
Medial lemniscus L	0.616(-0.739 - 1.97) x10^-7	0.369	-24.029(-51.893 - 3.834) x10^-6	0.431	3.719(-9.48 - 16.917) x10^-5	0.980
Inferior cerebellar peduncle R	-0.243(-2.093 - 1.606) x10^-7	0.794	0.664(-37.386 - 38.713) x10^-6	0.431	0.652(-17.372 - 18.675) x10^-5	0.980
Inferior cerebellar peduncle L	-0.675(-2.627 - 1.277) x10^-7	0.494	0.168(-39.98 - 40.316) x10^-6	0.431	-1.156(-20.173 - 17.862) x10^-5	0.980
Superior cerebellar peduncle R	0.154(-2.338 - 2.645) x10^-7	0.903	-14.597(-65.84 - 36.646) x10^-6	0.763	-3.768(-28.042 - 20.505) x10^-5	0.980
Superior cerebellar peduncle L	-0.157(-1.944 - 1.63) x10^-7	0.862	-3.48(-40.232 - 33.272) x10^-6	0.975	-8.406(-25.815 - 9.003) x10^-5	0.980
Cerebral peduncle R	-2.546(-4.196 - -0.896) x10^-7	0.003	-15.372(-49.312 - 18.569) x10^-6	0.431	7.809(-8.268 - 23.886) x10^-5	0.980
Cerebral peduncle L	-2.82(-4.48 - -1.161) x10^-7	0.001	-28.099(-62.238 - 6.04) x10^-6	0.348	1.537(-14.635 - 17.708) x10^-5	0.980
Anterior limb of internal capsule R	-1.679(-2.627 - -0.731) x10^-7	<0.001	-14.481(-33.976 - 5.014) x10^-6	0.431	-0.924(-10.158 - 8.311) x10^-5	0.980
Anterior limb of internal capsule L	-0.944(-1.842 - -0.046) x10^-7	0.04	-11.529(-30.008 - 6.95) x10^-6	0.431	9.308(0.554 - 18.061) x10^-5	0.444
Posterior limb of internal capsule R	-1.913(-2.833 - -0.993) x10^-7	<0.001	-14.956(-33.88 - 3.968) x10^-6	0.431	-0.995(-9.959 - 7.969) x10^-5	0.980
Posterior limb of internal capsule L	-1.634(-2.674 - -0.594) x10^-7	0.002	-21.375(-42.774 - 0.024) x10^-6	0.348	1.353(-8.783 - 11.49) x10^-5	0.980
Retrolenticular part of internal capsule R	-1.17(-2.052 - -0.288) x10^-7	0.01	5.226(-12.912 - 23.364) x10^-6	0.739	4.182(-4.41 - 12.773) x10^-5	0.980
Retrolenticular part of internal capsule L	-1.543(-2.404 - -0.683) x10^-7	<0.001	6.855(-10.85 - 24.56) x10^-6	0.799	2.28(-6.106 - 10.667) x10^-5	0.980
Anterior corona radiata R	-1.34(-2.144 - -0.536) x10^-7	0.001	8.893(-7.645 - 25.432) x10^-6	0.588	-3.089(-10.923 - 4.746) x10^-5	0.980
Anterior corona radiata L	-1.433(-2.3 - -0.567) x10^-7	0.001	19.148(1.319 - 36.978) x10^-6	0.431	1.141(-7.305 - 9.586) x10^-5	0.980
Superior corona radiata R	-1.027(-1.814 - -0.239) x10^-7	0.011	9.918(-6.289 - 26.125) x10^-6	0.564	4.763(-2.914 - 12.44) x10^-5	0.980
Superior corona radiata L	-0.932(-1.708 - -0.156) x10^-7	0.019	6.375(-9.589 - 22.339) x10^-6	0.739	7.183(-0.38 - 14.745) x10^-5	0.595
Posterior corona radiata R	-0.837(-1.888 - 0.214) x10^-7	0.117	20.682(-0.93 - 42.294) x10^-6	0.431	-0.564(-10.801 - 9.673) x10^-5	0.980
Posterior corona radiata L	-1.56(-2.479 - -0.642) x10^-7	0.001	17.427(-1.465 - 36.319) x10^-6	0.431	-2.321(-11.27 - 6.628) x10^-5	0.980
Posterior thalamic radiation R	-2.715(-3.743 - -1.687) x10^-7	<0.001	19.367(-1.779 - 40.512) x10^-6	0.431	-14.835(-24.851 - -4.818) x10^-5	0.192
Posterior thalamic radiation L	-2.083(-3.119 - -1.046) x10^-7	<0.001	10.444(-10.885 - 31.773) x10^-6	0.588	-12.582(-22.686 - -2.479) x10^-5	0.240
Sagittal stratum R	-0.734(-1.954 - 0.485) x10^-7	0.235	16.061(-9.022 - 41.144) x10^-6	0.431	1.998(-9.884 - 13.88) x10^-5	0.980
Sagittal stratum L	-0.968(-1.911 - -0.025) x10^-7	0.044	10.571(-8.82 - 29.962) x10^-6	0.473	-3.8(-12.985 - 5.385) x10^-5	0.980
External capsule R	-0.686(-1.346 - -0.026) x10^-7	0.042	-5.515(-19.089 - 8.058) x10^-6	0.588	-0.738(-7.168 - 5.691) x10^-5	0.980
External capsule L	-0.338(-0.932 - 0.257) x10^-7	0.262	-4.205(-16.431 - 8.02) x10^-6	0.564	2.706(-3.085 - 8.498) x10^-5	0.980
Cingulum cingulate gyrus R	-1.671(-2.935 - -0.408) x10^-7	0.01	17.171(-8.828 - 43.17) x10^-6	0.431	-8.723(-21.038 - 3.593) x10^-5	0.980
Cingulum cingulate gyrus L	0.061(-1.342 - 1.464) x10^-7	0.931	13.006(-15.858 - 41.871) x10^-6	0.588	3.622(-10.051 - 17.295) x10^-5	0.980
Cingulum hippocampus R	-1.185(-2.713 - 0.342) x10^-7	0.127	15.063(-16.354 - 46.479) x10^-6	0.485	1.689(-13.193 - 16.571) x10^-5	0.980
Cingulum hippocampus L	-0.775(-2.36 - 0.81) x10^-7	0.334	0.691(-31.911 - 33.293) x10^-6	0.849	7.345(-8.099 - 22.788) x10^-5	0.980
Fornix Stria terminalis R	-0.864(-2.287 - 0.558) x10^-7	0.23	20.672(-8.583 - 49.926) x10^-6	0.431	-0.659(-14.517 - 13.198) x10^-5	0.980
Fornix Stria terminalis. L	-0.728(-1.804 - 0.348) x10^-7	0.182	7.548(-14.584 - 29.679) x10^-6	0.663	-0.133(-10.616 - 10.351) x10^-5	0.980
Superior longitudinal fasciculus R	-0.774(-1.484 - -0.063) x10^-7	0.033	9.739(-4.879 - 24.357) x10^-6	0.431	-2.151(-9.075 - 4.773) x10^-5	0.980
Superior longitudinal fasciculus L	-0.426(-1.185 - 0.333) x10^-7	0.267	9.414(-6.197 - 25.025) x10^-6	0.431	-0.372(-7.767 - 7.023) x10^-5	0.980
Superior fronto.occipital fasciculus R	-1.295(-2.444 - -0.146) x10^-7	0.028	-3.78(-27.409 - 19.85) x10^-6	0.695	-2.235(-13.428 - 8.958) x10^-5	0.980
Superior fronto.occipital fasciculus L	-0.839(-2.023 - 0.345) x10^-7	0.163	18.913(-5.442 - 43.268) x10^-6	0.431	2.265(-9.272 - 13.802) x10^-5	0.980
Uncinate fasciculus R	-2.479(-3.614 - -1.345) x10^-7	<0.001	-2.493(-25.822 - 20.835) x10^-6	0.588	1.256(-9.794 - 12.307) x10^-5	0.980
Uncinate fasciculus L	-1.46(-2.645 - -0.276) x10^-7	0.016	-17.801(-42.162 - 6.559) x10^-6	0.431	-5.46(-16.999 - 6.079) x10^-5	0.980
Tapetum R	-0.979(-4.246 - 2.287) x10^-7	0.553	47.668(-19.524 - 114.859) x10^-6	0.431	5.339(-26.489 - 37.167) x10^-5	0.980
Tapetum L	-0.423(-3.103 - 2.258) x10^-7	0.755	60.409(5.276 - 115.542) x10^-6	0.348	4.512(-21.604 - 30.628) x10^-5	0.980

**Edge Density**	**Age**		**ASD**		**Age**	
	**Coefficient (95% CI)**	**p-value**	**Coefficient (95% CI)**	**p-value**	**Coefficient (95% CI)**	**p-value**
Middle cerebellar peduncle	93.07(-0.94 - 187.07)	0.052	15.73(-3.6 - 35.07)*10^3	0.11	-77.65(-169.24 - 13.94)*10^3	0.752
Genu of corpus callosum	-0.11(-56.53 - 56.31)	0.997	-5.1(-16.7 - 6.51)*10^3	0.385	55.28(0.31 - 110.26)*10^3	0.196
Body of corpus callosum	-11.15(-68.24 - 45.94)	0.699	-6.74(-18.48 - 5)*10^3	0.257	52.47(-3.16 - 108.09)*10^3	0.210
Splenium of corpus callosum	-63.16(-119.36 - -6.97)	0.028	-10.22(-21.77 - 1.34)*10^3	0.083	45.2(-9.55 - 99.95)*10^3	0.623
Fornix	-329.44(-545.48 - -113.4)	0.003	-56.13(-100.56 - -11.69)*10^3	0.014	110.95(-99.55 - 321.45)*10^3	0.262
Inferior cerebellar peduncle R	113.97(48.19 - 179.75)	<0.001	2.65(-10.88 - 16.18)*10^3	0.698	-57.47(-121.57 - 6.62)*10^3	0.403
Inferior cerebellar peduncle L	159.54(95.6 - 223.47)	<0.001	2.79(-10.36 - 15.94)*10^3	0.674	-61.11(-123.41 - 1.18)*10^3	0.475
Superior cerebellar peduncle R	16.22(-9.25 - 41.69)	0.209	0.85(-4.39 - 6.1)*10^3	0.747	-14(-38.77 - 10.77)*10^3	0.433
Superior cerebellar peduncle L	32.65(4.25 - 61.05)	0.025	2.38(-3.46 - 8.22)*10^3	0.421	-23.39(-51.06 - 4.28)*10^3	0.383
Cerebral peduncle R	13.02(-32.58 - 58.62)	0.572	-6.68(-16.06 - 2.7)*10^3	0.16	-16.39(-60.82 - 28.04)*10^3	0.288
Cerebral peduncle L	-15.95(-75.2 - 43.31)	0.594	1.55(-10.64 - 13.74)*10^3	0.801	-1.67(-59.4 - 56.07)*10^3	0.109
Anterior limb of internal capsule R	50.5(-51.56 - 152.55)	0.328	-6.92(-27.91 - 14.07)*10^3	0.514	116.16(16.72 - 215.6)*10^3	0.082
Anterior limb of internal capsule L	37.66(-75.71 - 151.03)	0.511	-18.16(-41.47 - 5.16)*10^3	0.125	158(47.54 - 268.46)*10^3	<0.001
Posterior limb of internal capsule R	-49.75(-139.63 - 40.12)	0.274	-30.32(-48.81 - -11.83)*10^3	0.002	22.34(-65.23 - 109.91)*10^3	0.109
Posterior limb of internal capsule L	-33.49(-161.36 - 94.38)	0.604	-19.85(-46.15 - 6.46)*10^3	0.137	-10.6(-135.19 - 113.98)*10^3	0.062
Retrolenticular part of internal capsule R	-67.38(-280.2 - 145.44)	0.531	-47.8(-91.58 - -4.03)*10^3	0.033	13.76(-193.61 - 221.12)*10^3	0.097
Retrolenticular part of internal capsule L	-31.09(-284.83 - 222.65)	0.808	-56.58(-108.77 - -4.38)*10^3	0.034	65.07(-182.16 - 312.3)*10^3	0.109
Anterior corona radiata R	52.05(-21.09 - 125.2)	0.161	-9.82(-24.87 - 5.23)*10^3	0.198	42.27(-29 - 113.54)*10^3	0.185
Anterior corona radiata L	10.27(-82.78 - 103.33)	0.827	-11.89(-31.03 - 7.25)*10^3	0.22	24.67(-66 - 115.34)*10^3	0.097
Superior corona radiata R	24.1(-62.45 - 110.66)	0.582	-11.86(-29.66 - 5.95)*10^3	0.189	86.38(2.05 - 170.72)*10^3	0.240
Superior corona radiata L	-45.68(-140 - 48.65)	0.339	-14.58(-33.99 - 4.82)*10^3	0.139	61.17(-30.73 - 153.08)*10^3	0.097
Posterior corona radiata R	-9.95(-109.59 - 89.69)	0.843	-0.69(-21.19 - 19.8)*10^3	0.947	56.43(-40.65 - 153.51)*10^3	0.456

Posterior corona radiata L	-111.21(-224.36 - 1.95)	0.054	-0.63(-23.9 - 22.65)*10^3	0.957	54.75(-55.51 - 165)*10^3	0.398
Posterior thalamic radiation R	12(-66.3 - 90.29)	0.762	-31.36(-47.46 - -15.25)*10^3	<0.001	0.69(-75.59 - 76.98)*10^3	0.262
Posterior thalamic radiation L	-31.18(-108.55 - 46.19)	0.426	-17.86(-33.77 - -1.94)*10^3	0.028	17.22(-58.17 - 92.61)*10^3	0.384
Sagittal stratum R	30.65(-110.22 - 171.53)	0.667	-17.06(-46.03 - 11.92)*10^3	0.245	5.05(-132.21 - 142.31)*10^3	0.185
Sagittal stratum L	112.01(-21.51 - 245.54)	0.099	-5.83(-33.3 - 21.63)*10^3	0.674	-64.87(-194.97 - 65.23)*10^3	0.188
External capsule R	-79.5(-242.5 - 83.49)	0.335	-20.15(-53.68 - 13.38)*10^3	0.236	29.71(-129.11 - 188.53)*10^3	0.062
External capsule L	-145.87(-299.96 - 8.23)	0.063	-20.68(-52.37 - 11.02)*10^3	0.198	44.01(-106.14 - 194.15)*10^3	0.068
Cingulum cingulate gyrus R	65.1(-55.42 - 185.61)	0.286	-10.64(-35.43 - 14.15)*10^3	0.396	-66.54(-183.97 - 50.89)*10^3	0.230
Cingulum cingulate gyrus L	46.28(-67.3 - 159.87)	0.42	-17.22(-40.59 - 6.14)*10^3	0.147	-48.72(-159.4 - 61.95)*10^3	<0.001
Cingulum hippocampus R	3.21(-72.35 - 78.77)	0.933	-0.5(-16.04 - 15.04)*10^3	0.949	-31(-104.62 - 42.63)*10^3	0.109
Cingulum hippocampus L	5.84(-61.91 - 73.59)	0.864	-8.27(-22.2 - 5.67)*10^3	0.242	-40.41(-106.42 - 25.6)*10^3	0.196
Fornix Stria terminalis R	-151.05(-249.47 - -52.64)	0.003	-28.81(-49.06 - -8.57)*10^3	0.006	67.44(-28.46 - 163.33)*10^3	0.196
Fornix Stria terminalis. L	-93.99(-199.11 - 11.13)	0.079	-25(-46.62 - -3.38)*10^3	0.024	7.47(-94.95 - 109.9)*10^3	0.098
Superior longitudinal fasciculus R	121.73(18.44 - 225.03)	0.021	-7.48(-28.73 - 13.77)*10^3	0.486	-54.32(-154.97 - 46.33)*10^3	0.154
Superior longitudinal fasciculus L	50.39(-69.75 - 170.53)	0.407	-19.48(-44.2 - 5.23)*10^3	0.121	18.67(-98.39 - 135.72)*10^3	0.097
Superior fronto.occipital fasciculus R	130.82(-55.63 - 317.27)	0.167	-7.32(-45.67 - 31.03)*10^3	0.706	28.02(-153.64 - 209.69)*10^3	0.443
Superior fronto.occipital fasciculus L	-64.12(-288.4 - 160.15)	0.572	-51.41(-97.54 - -5.28)*10^3	0.029	76.76(-141.76 - 295.28)*10^3	0.098
Uncinate fasciculus R	-98.73(-203.24 - 5.78)	0.064	-12.2(-33.7 - 9.29)*10^3	0.262	4.64(-97.19 - 106.47)*10^3	0.230
Uncinate fasciculus L	-304.97(-549.16 - -60.79)	0.015	-6.41(-56.64 - 43.81)*10^3	0.8	-20.97(-258.89 - 216.95)*10^3	0.109
Tapetum R	-57.48(-166.32 - 51.36)	0.297	-16.95(-39.34 - 5.44)*10^3	0.136	4.28(-101.77 - 110.33)*10^3	0.864
Tapetum L	-79.12(-168.44 - 10.19)	0.082	-20.92(-39.29 - -2.55)*10^3	0.026	61.81(-25.22 - 148.83)*10^3	0.807

References

[1] S. P. Haider *et al.*, "PET/CT radiomics signature of human papilloma virus association in oropharyngeal squamous cell carcinoma," *Eur J Nucl Med Mol Imaging,* vol. 47, no. 13, pp. 2978-2991, Dec 2020, doi: 10.1007/s00259-020-04839-2.

[2] J. Friedman, T. Hastie, and R. Tibshirani, "Regularization Paths for Generalized Linear Models via Coordinate Descent," *Journal of Statistical Software,* vol. 33, no. 1, pp. 1-22, 2010, doi: 10.18637/jss.v033.i01.

[3] M. Majka, "naivebayes: High Performance Implementation of the Naive Bayes Algorithm in R," ed: CRAN, 2019.

[4] L. Breiman, "Random Forests," *Machine Learning,* vol. 45, pp. 5-32, 2001, doi: 10.1023/A:1010933404324.

[5] M. Wiener and A. Liaw, "Package 'randomForest' Breiman and Cutler's Random Forests for Classification and Regression," ed. CRAN, 2002.

[6] D. Meyer *et al.*, "Package 'e1071'," ed. CRAN, 2021.

[7] D. Meyer and F. T. Wien, "Support Vector Machines," in *The Interface to libsvm in package e1071*, ed: CRAN, 2015.

[8] T. Chen *et al.*, "Package 'xgboost'," ed. CRAN, 2021.

[9] T. Chen and C. Guestrin, "XGBoost: A Scalable Tree Boosting System," ed: Association for Computing Machinery, 2016.

[10] F. Murtagh and P. Legendre, "Ward's Hierarchical Agglomerative Clustering Method: Which Algorithms Implement Ward's Criterion?," *Journal of Classification,* vol. 31, pp. 274-295, 10 2014, doi: 10.1007/s00357-014-9161-z.

[11] N. De Jay, S. Papillon-Cavanagh, C. Olsen, N. El-Hachem, G. Bontempi, and B. Haibe-Kains, "MRMRe: An R package for parallelized mRMR ensemble feature selection," *Bioinformatics,* vol. 29, pp. 2365-2368, 9 2013, doi: 10.1093/bioinformatics/btt383.

[12] M. B. Kursa, "Package 'praznik'," ed. CRAN, 2020.

[13] C. E. Shannon, "A Mathematical Theory of Communication," *The Bell System Technical Journal,* vol. 27, pp. pp. 379–423, 623–656, 7 1948.

[14] M. Kuhn *et al.*, "Package 'caret'," ed. CRAN, 2021.

[15] F. Song, Z. Guo, and D. Mei, "Feature Selection Using Principal Component Analysis," *International Conference on System Science, Engineering Design and Manufacturing Informatization,* vol. 1, pp. 27-30, 2010, doi: 10.1109/ICSEM.2010.14.
